# A Cluster Randomised Trial on the Impact of Integrating Early Infant HIV Diagnosis with the Expanded Programme on Immunization on Immunization and HIV Testing Rates in Rural Health Facilities in Southern Zambia

**DOI:** 10.1371/journal.pone.0141455

**Published:** 2015-10-29

**Authors:** Paul C. Wang, Albert Mwango, Sarah Moberley, Benjamin J. Brockman, Alison L. Connor, Penelope Kalesha-Masumbu, Simon Mutembo, Maximillian Bweupe, Pascalina Chanda-Kapata, Godfrey Biemba, Davidson H. Hamer, Benjamin Chibuye, Elizabeth McCarthy

**Affiliations:** 1 IDinsight Zambia, IDinsight, Lusaka, Zambia; 2 Directorate of Disease Surveillance and Research, Ministry of Health, Lusaka, Zambia; 3 Applied Analytics Team, Clinton Health Access Initiative, Melbourne, Victoria, Australia; 4 Child Health, Zambian Ministry of Community Development, Maternal and Child Health, Lusaka, Zambia; 5 Country Office, Zambian Centre for Applied Health Research and Development, Lusaka, Zambia; 6 Center for Global Health and Development, Boston University School of Public Health, Boston, Massachusetts, United States of America; 7 Department of Global Health, Boston University School of Public Health, Boston, Massachusetts, United States of America; 8 Country Office, Clinton Health Access Initiative, Lusaka, Zambia; 9 Applied Analytics Team, Clinton Health Access Initiative, Lusaka, Zambia; The George Washington University School of Medicine and Health Sciences, UNITED STATES

## Abstract

**Background:**

We assessed the integration of early infant HIV diagnosis with the expanded programme for immunization in a rural Zambian setting with the aim of determining whether infant and postpartum maternal HIV testing rates would increase without harming immunization uptake.

**Methods:**

In an unblinded, location stratified, cluster randomised controlled trial, 60 facilities in Zambia’s Southern Province were equally allocated to a control group, Simple Intervention group that received a sensitization meeting and the resupply of HIV testing commodities in the event of a stock-out, and a Comprehensive Intervention group that received the Simple Intervention as well as on-site operational support to facilitate the integration of HIV testing services with EPI.

**Findings:**

The average change in number of first dose diphtheria, pertussis, and tetanus vaccine (DPT1) provided per month, per facility was approximately 0.86 doses higher [90% confidence interval (CI) -1.40, 3.12] in Comprehensive Intervention facilities compared to the combined average change in the Simple Intervention and control facilities. The interventions resulted in a 16.6% [90% CI: -7%, 46%, P-value = 0.26] and 10% [90% CI: -10%, 36%, P-value = 0.43] greater change in average monthly infant DBS testing compared to control for the Simple and Comprehensive facilities respectively. We also found 15.76 (90% CI: 7.12, 24.41, P-value < 0.01) and 10.93 (90% CI: 1.52, 20.33, P-value = 0.06) additional total maternal re-tests over baseline for the Simple and Comprehensive Facilities respectively.

**Conclusions:**

This study provides strong evidence to support Zambia’s policy of integration of HIV testing and EPI services. Actions in line with the interventions, including HIV testing material supply reinforcement, can increase HIV testing rates without harming immunization uptake. In response, Zambia’s Ministry of Health issued a memo to remind health facilities to provide HIV testing at under-five clinics and to include under-five HIV testing as part of district performance assessments.

**Trial Registration:**

ClinicalTrials.gov Registration Number: NCT02479659

## Introduction

Early identification of and the provision of antiretroviral therapy (ART) for HIV-positive infants are critical to improving infant survival. In the absence of treatment, 52.5% of HIV-infected children will die by the age of two, with most deaths occurring in the first year [1]. Early initiation of ART (between 6–12 weeks) has been shown to result in a 76% relative reduction in early mortality [2].

However, the provision of early infant diagnosis (EID) and ART services to infants in low-income countries has been challenging, with infants “lost” at each step of the HIV care continuum, including identification of HIV exposure, testing, delivery of results, and initiation of treatment. In Zambia, an estimated 80,000 to 90,000 HIV-positive women give birth each year, but only about 48,000 of those infants were given a DNA PCR test in 2012 [3].

Zambia’s Ministry of Health (MOH) provides the following recommendations for HIV testing of new mothers and newborns:

- All breastfeeding and recently breastfeeding mothers who have an unknown or previously negative HIV-status should receive a repeat HIV antibody test every three months until the infant reaches 18 months of age.
- All HIV-exposed infants should receive a dried blood spot (DBS) HIV test at 6-weeks and 6 months of life [4].

Given the high coverage of the Expanded Programme for Immunization (EPI) and alignment of the schedule with key HIV testing touch-points, integrating HIV testing with EPI services could increase HIV testing coverage and reduce costs by pooling financial and human resources [5]. However, many facilities do not provide any HIV services during EPI activities. Those that do offer HIV testing services during EPI activities largely do so in an ad-hoc and opt-in manner that relies on the initiative of staff to organize services and to determine who is in need of testing.

At the implementation level, HIV testing services may not be offered during EPI activities for a variety of reasons, including concerns of potential harm to immunization uptake, lack of testing supplies, lack of knowledge of existing recommendations, and clinic-level operational constraints.

Evidence supporting the integration of EID with EPI is available from four observational studies conducted in Zimbabwe [6, 7], South Africa [8] and Malawi [9] which indicate integration of HIV-testing for mothers and their babies with routine immunization services is feasible and likely to be effective at improving rates of EID.

In Zimbabwe, EID-EPI integration increased cotrimoxazole initiation (from 182 to 565 infants, 210% increase) for exposed infants and HIV testing (from 74 to 128 infants, 73% increase) compared to the same time in the previous year [6]. In Malawi, EID-EPI integration also resulted in a higher rate of infant testing (84.2% vs. 11.4%, P-value < 0.01) [9].

Other studies have found high levels of acceptance of EID-EPI integration. In KwaZulu Natal, South Africa, 90.4% of mothers agreed to HIV testing when offered during EPI visits in three health facilities. Most mothers that were interviewed stated that they were comfortable with the integration of services [8]. Furthermore, a qualitative study conducted across Kenya, Mali, Ethiopia and Cameroon indicated that the integration of services was acceptable by health care workers and patients [10].

The aim of this study was to use an experimental evaluation methodology to determine whether the formal integration of EID with EPI could increase HIV testing rates without harming immunization uptake so as to ensure existing MOH policy could be fully implemented.

### Ethical approval

This evaluation was approved by the ERES Converge Ethics Review Board in Lusaka, Zambia, on May 23, 2013, and the Boston University Institutional Review Board in Boston, Massachusetts, United States, on July 16, 2013. Formal approvals were also obtained from Zambia’s MOH, Ministry of Community Development, Mother and Child Health, and the District Health Offices of Choma, Livingstone, and Monze. The primary aims of the study relied solely on de-identified administrative data that were provided by study facilities. Therefore, no infants or mothers included in the HIV testing or immunization outcomes were enrolled in the study, and they were not requested to provide informed consent. Women and facility staff who participated in interviews and focus group discussions, however, did complete an informed consent process. They were read informed consent documents in the relevant language. Participants acknowledged confirmation that they had been read the informed consent and that they agreed to participate by signing or providing a thumbprint on consent forms. These procedures were approved by both review boards.

## Materials and Methods

### Study setting

This evaluation took place in Livingstone, Monze, and Choma districts in the Southern Province of Zambia. Zambia’s Southern Province is home to 1.5 million people with 75% of its population living in rural settings and 48% of its population below the age of 15 [11]. In 2007, it was estimated that 14.5% of the women and men age 15–49 who received an HIV test in Southern Province were seropositive [11]. The selection of Southern Province was based on geographic dispersion, urban/rural characteristics, current HIV prevalence rates, and the absence of conflicting research projects.

### Study interventions

This evaluation had three study groups:

- **Control group**: Facilities continued usual care.
- **Simple Intervention group**: HIV testing commodities were replenished directly (outside of the government supply) in the event of a stock-out, and a sensitization meeting with facility staff was held by district health officials to remind providers of current policy on integration of HIV testing and immunization services.
- **Comprehensive Intervention group**: Both aspects of the Simple Intervention were administered as part of the Comprehensive Intervention. Additionally, on-site operational support was provided to facilitate the integration of EID with EPI at the health facility, which included guidance on how to optimize staffing and patient flow. Facility staff were instructed to administer dried blood spot (DBS) tests on all infants with known HIV-infected mothers and HIV antibody tests on all mothers with unknown or previously negative status who were either attending their infant’s first immunization visit or had not been tested in the previous three months. All tests were offered in an opt-out manner. Facility staff were also instructed to provide group counselling to caregivers on the HIV testing service provided and general HIV testing education. These activities were supported by a community awareness campaign, whereby health volunteers conducted sensitization meetings at communities in the facility catchment area and at the beginning of each under-five clinic to inform community members of changes to the under-five clinics.

In all Simple and Comprehensive Intervention facilities, routine testing procedures were followed using the rapid Determine^TM^ screening antibody test and Unigold^TM^ confirmatory test along with all standard counselling messaging per existing practice. Infants of mothers who tested HIV-seropositive then received a DNA PCR test the same day. The intervention was implemented from October 1, 2013–March 31, 2014. Postpartum mothers and infants who received an HIV test, as well as infants who received the first dose of the combined vaccine for diphtheria, pertussis and tetanus (DPT1) during this period were included in the outcome. Since the main quantitative study relied on administrative data, there was no follow-up period.

### Sampling

This cluster randomised controlled evaluation was conducted in sixty government-run health facilities that provide prevention of mother to child transmission (PMTCT) and EID services. These facilities were supported by the Zambian Centre for Applied Health Research and Development (ZCAHRD), an implementing partner that provided training for HIV service provision and staff support, including the training and hiring of one PMTCT lay counsellor per facility. Hospitals and hospital affiliated health centres were excluded from sampling to avoid catchment area overlap with other study facilities. Health facilities without a full time nurse or midwife trained to administer DBS tests were also excluded.

**Fig 1:**
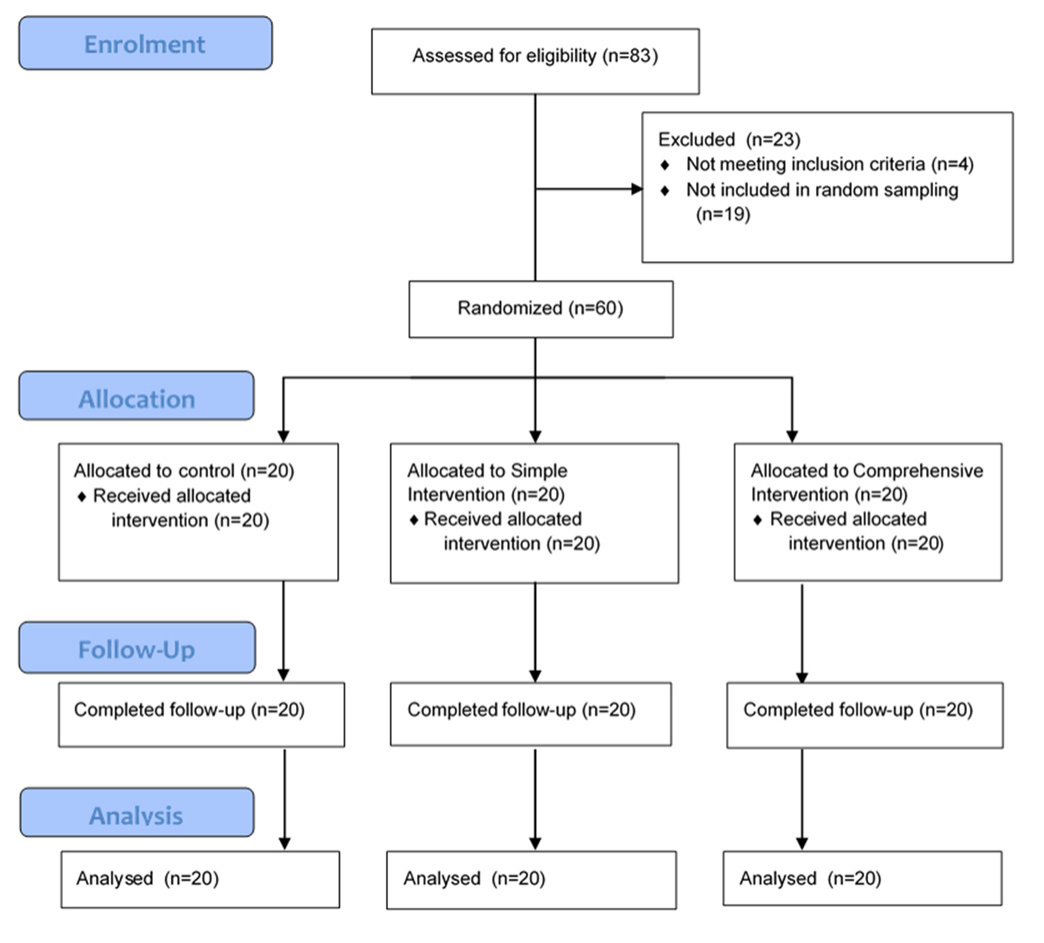
Flowchart of facility inclusion and sampling. Health facilities were used as the unit of sampling, randomization, follow-up and analysis.

All eligible facilities were stratified based on their location (district and urban/ rural) (Fig 1). Within each stratum, facilities were randomly selected for inclusion by the study team and then ranked based on their average number of vaccines provided per month. The top three ranked facilities were randomly allocated into one of the three study arms using a random number generator, followed by the next three ranked facilities, and so on.

### Data collection

Since the main study relied solely on administrative data, there was no participant recruitment and follow-up. Patients were eligible to be included in the outcome measures if they attended an under-five clinic at one of the study facilities during the baseline period (January 2012–August 2013) or during the intervention period (September 2013–March 2014) and received a first dose diphtheria, pertussis, and tetanus vaccine (DPT1) immunization, an infant DBS test, or a maternal postpartum HIV test. Research team members conducted monthly visits to all 60 study facilities to collect data from facility registers, HIV Activity Sheets, and under-five clinic tally sheets. Register data were collected using Open Data Kit (ODK) surveys programmed on Android mobile phones. During these monthly data collection visits, the evaluation team also briefly interviewed facility staff to discuss any staffing changes and to take stock of HIV testing and immunization supplies.

The primary outcomes were the average number of DBS tests performed per facility per month, the average monthly number of maternal postpartum retests performed per facility per month and the average monthly number of DPT1 doses administered per facility per month. Outcomes measurements are described below.

#### Average Monthly Number of DBS Tests

The average number of DBS tests conducted at each facility per month was measured from January 2012 to March 2014 using data from the DBS lab database at University Teaching Hospital in Lusaka. A DBS test was included if the sample arrived at the lab. These data were verified using DBS test figures from facility records. DBS tracking registers were used to verify the first and second DBS tests, and facility lab requisition registers were used to verify the total number of tests conducted.

#### Average Monthly Number of Maternal Retests

The average number of monthly maternal retests was measured from January 2013 to March 2014 using data from the Boston University PMTCT Integration Programme (BUPIP) monthly report. This outcome included all retests that were recorded, regardless of the test result. These data were verified using the facility HIV activity sheets at intervention facilities (additional data were not collected from control facilities). The pre-analysis plan had initially intended for this outcome to be measured as the percentage of mothers attending static under-five clinic who receive maternal retests. However, this was changed to a count of the number of maternal retests administered since all facilities did not disaggregate under-five data by static and outreach services.

#### Average Monthly Number of DPT1 Doses

DPT1 was used as the proxy for immunization uptake since, like EID, it is given to children at six weeks, following the recommended World Health Organization EPI guidelines. The number of DPT1 doses was measured from January 2012 to March 2014 using figures that facilities routinely report to the District Health Officer (DHO). These estimates included doses provided at both outreach and within the facility, since the report does not disaggregate these figures. Figures were compared to the number of DPT1 doses recorded on each under-five tally sheet for a given month. If there was a >10% discrepancy between these data sources, the research team reconciled the data using pre-established data cleaning rules.

#### Covariates

Facilities were determined to be rural if they were ≥10 kilometers from the DHO, based on administrative reports. The average baseline number of mothers that attended antenatal clinic (ANC) and the number of mothers appearing for their first antenatal visit were collected using routine monthly reports from facilities. The number of HIV seropositive mothers anticipated at under-five clinics was calculated by multiplying the average baseline number of mothers attending first ANC visit by the percentage of women attending ANC that were living with HIV, as supplied by the BUPIP database. The actual distance between the facility and the DHO was measured using GPS coordinates.

### Interim analysis

An interim analysis using immunization data from October–December 2013 was planned to test for any serious adverse effects on immunization rates resulting from the Comprehensive Intervention. For the purposes of this analysis, facilities in the Simple Intervention group was combined with the control facilities, since we did not expect the Simple Intervention to have an adverse effect on immunizations. If the mid-term analysis found a decrease in static DPT1 immunizations of 20% or greater among the 20 Comprehensive facilities compared to 40 Control and Simple facilities, and this difference was statistically significantly different from a 0% change at significance level p = 0.05, the research team would stop the intervention, notify the Data and Safety Monitoring Board (DSMB), and provide project data and analysis.

This analysis was reviewed by three independent reviewers. The analysis had to be modified slightly to include all DPT1 immunizations administered, since immunization data were not disaggregated by static versus outreach at all facilities. Four linear regression models with different combinations of covariates were tested. The point estimates for all four models on the coefficient of interest were all approximately zero. The lower bound of the 95% confidence interval for all four models did not exceed -11.5 immunizations, which would represent a 14 percentage point drop–short of the 20% immunization threshold.

### Focus group discussions

Focus groups were conducted between May 11–May 22, 2014 in catchment area communities of eight study facilities in the Simple or Comprehensive Intervention groups to understand reasons why mothers do or do not attend under-five services and perceptions of the changes at under-five clinic regarding HIV testing. These facilities were purposively sampled across intervention arms to achieve a mix of urban and rural settings and large and small facilities. In each location, community health workers identified women whose babies would have been eligible for six week vaccination during the intervention period. After the age of the baby was confirmed, women were invited to participate, and study staff read aloud an informed consent statement. All literate women provided written consent to participate. In cases where a woman was unable to sign her name, her thumbprint was taken instead. This consent procedure was approved by ERES Converge Ethics Review Board and Boston University Institutional Review Board. All focus groups were conducted in the local language of Tonga and responses were coded according to common themes by two separate evaluation staff members.

### Study Registration

This study was not registered prior to the enrolment of participants. This study was completed at the request of the Government of Zambia, and results were intended to be used by the Government of Zambia to inform policy. Making findings public was not discussed at this time, so registration did not happen prior to enrolment. It was registered on June 14, 2015 in the ClinicalTrials.gov registry (NCT02479659). The authors confirm that all ongoing and related trials for this intervention are registered.

### Statistical Methods

#### Power calculation

The per protocol power calculation was based on a cluster randomised design with binary, person-level outcomes, with a minimum detectable effect size of 10% change in average number of DPT1 doses (α = 0.1, power = 0.8). Since it was not feasible to conduct the analysis with individual level outcomes, the analytical approach was therefore adjusted to become a levels-based, difference-in-difference analysis and post-hoc power calculations increased the minimum detectable effect size to 13% for the average number of DPT1 doses. These power calculations did not adjust for stratification or the use of any covariates in the analysis.

#### Statistical analysis

The primary outcomes were a comparison of the average number of DPT1 doses per month and changes in infant and maternal HIV tests conducted between intervention and control facilities. Multivariate linear regression models using a clustered Huber-White sandwich estimator accounting for clustering at the facility level were used to estimate the differences in the change of each outcome between the facilities in each treatment arm and the control arm. Clustered standard errors were estimated using bootstrapping methods. The regression for the DBS testing outcome used a log-transformation to normalize the distribution of the outcome. The isolated effect of each intervention on each of the primary outcomes was estimated by including interaction terms between a factor variable for the intervention groups and the time period (baseline versus intervention period) [12]. Additional covariates were included in the regression models to control for confounders and to increase precision. All regression models were adjusted for district and urban/rural characteristics, since these are the variables on which the initial sample was stratified. The regression model for the difference in the change in average number of DBS tests also adjusted for time (using splines to account for linear trends), a binary indicator for a period of a national stockout, the number of DPT1 doses provided in the same month, and the number of mothers living with HIV anticipated in that month (calculated as the average number of women attending first antenatal care (ANC) visit multiplied by the percentage of women attending first ANC visit who are living with HIV, as supplied by the BUPIP database). The regression model for the average number of maternal retests administered adjusted for district and urban/rural characteristics, time, the monthly average number of mothers going to first ANC, the distance between the facility and the DHO, and the number of DPT1 doses provided in the same month. Finally, the regression model for the average number of DPT1 doses provided each month adjusted for the average number of mothers going to first ANC, time, and the average distance from the DHO. Ninety-percent confidence intervals were stated to be the policy-relevant standard by the Ministry of Health.

Focus group discussions were analysed using a thematic analysis approach.

All statistical analyses were done using Stata version 12 (Stata Corp LP, College Station, Texas, US). Statistical significance was set at the 0.1 level, based on the discussions with the MOH regarding the required power of the study to influence policy.

## Results

Baseline characteristics of monthly average DBS tests, DPT1 doses, first ANC visits, 6-week retests, and total retests appeared similar across randomization groups, with no significant differences between groups. Although the mean baseline number of 6-week retests and total number of retests appeared lower for the Simple Intervention group, the differences were not significant (Table 1).

**Table 1:** Summary and baseline characteristics by intervention arm.

	**Control (N = 20)**		**Simple (N = 20)**		**Comprehensive (N = 20)**		**Total N**
	**N**	**(%)**	**N**	**(%)**	**N**	**(%)**	
***Facility Characteristics***							
No ART	14	(70%)	15	(75%)	15	(75%)	44
Facility-based ART	2	(10%)	3	(15%)	4	(20%)	9
Mobile ART	4	(20%)	2	(10%)	1	(5%)	7
***Strata***							
Choma Rural	8	(40%)	8	(40%)	8	(40%)	24
Choma Urban	2	(10%)	2	(10%)	2	(10%)	6
Livingstone Urban	3	(15%)	3	(15%)	3	(15%)	9
Monze Rural	6	(30%)	6	(30%)	6	(30%)	18
Monze Urban	1	(5%)	1	(5%)	1	(5%)	3
	**Mean**	**(SD)**	**Mean**	**(SD)**	**Mean**	**(SD)**	P-value*
***Baseline Monthly Averages***							
DBS tests	3.97	(7.05)	3.74	(6.98)	4.30	(8.08)	0.89
DPT1 doses	28.67	(23.43)	27.26	(17.42)	26.23	(26.75)	0.99
First ANC Visits	30.71	(25.04)	29.32	(21.44)	29.9	(21.44)	0.94
6 Week Retests	3.29	(3.73)	1.18	(1.81)	3.04	(5.31)	0.14
Total Retests	14.21	(15.64)	6.74	(9.38)	15.16	(27.16)	0.19

* The intervention arms were regressed as indicator variables on each outcome. After the regression, an F-test was used to test for equality between the three evaluation arms. At the time of sampling, all samples with p-values of less than 0.90 for DBS, DPT, and ANC averages were removed from consideration. Since that time, more complete data are available, explaining the 0.89 p-value for the DBS tests at the final sample.

There were a total of 136 occasions where testing commodities were re-stocked at both the Comprehensive and Simple Intervention facilities during the intervention period when stocks were low or had run out (including Determine^TM^ Antibody Tests, Unigold^TM^ confirmatory test and infant dry blood spot tests). However, intervention facility staff reported that any period of stock out was only for one to two days and did not influence their ability to conduct the intervention. Reports from facility staff in the control facilities suggested that stockouts occurred with similar frequency to the restocks in intervention facilities. It was also reported in some instances, the stockouts in control facilities lasted over a month because they were not restocked. We did not collect systematic data on the period of time that commodities stocked-out.

### Impact of interventions on DPT1 doses

The baseline period for this analysis ran from January 1, 2012 to September 30, 2013. The months between August and November 2012 –when all facilities experienced a sharp drop in immunization numbers due to a national stock out–were excluded from the analysis. A total of 10,435 doses of DPT1 were administered across the 60 evaluation facilities during the six-month intervention period between October 1, 2013 and March 31, 2014.

The average number of DPT1 doses was slightly higher during the intervention period across all study arms (6.7%, 3.2% and 7.7% more doses per month over baseline in control, Simple Intervention and Comprehensive Intervention facilities respectively). The facility-based difference between the baseline and intervention periods in the number of monthly DPT1 doses administered was 1.93 (standard deviation (SD) 5.97) in the control arm, compared to 0.86 (SD 4.08) in the Simple Intervention arm and 2.01 (SD 5.56) in the Comprehensive Intervention arm (Table 2).

**Table 2:** Average number of DPT1 doses administered per month by randomisation group.

	Control		Simple		Comprehensive	
	Mean	SD	Mean	SD	Mean	SD
Baseline Period Average	28.67	(23.43)	27.26	(17.42)	26.23	(26.75)
Intervention Period Average	30.60	(23.25)	28.12	(18.00)	28.24	(29.05)
Difference	1.93	(5.97)	0.86	(4.08)	2.01	(5.56)
Proportional change	6.7%		3.2%		7.7%	

Multivariate linear regression estimated that the average change in number of DPT1 doses provided per month, per facility was approximately 0.86 doses higher [90% confidence interval (CI) -1.40, 3.12] in Comprehensive Intervention facilities compared to the combined average change in the Simple Intervention and control facilities (Table 3). This average change was not statistically significant (p-value 0.53). A similar multivariate linear regression was run to estimate the combined impact of the Comprehensive and Simple Interventions versus control on the monthly number of DPT1 doses administered, with an average -0.51 fewer doses administered (90% CI -3.59, 2.56, p value 0.78) (data not shown).

**Table 3:** Multivariate Linear Regression Results for the Montly Average Number of DPT1 Doses Comparing Comprehensive Group to Combined Control & Simple Groups.

Covariates	Coeff	P-value	[90% CI]
***Intervention Arm***			
Control & Simple	Ref		
Comprehensive	-0.868	0.60	[-3.62,1.89]
***Time Period***			
Baseline	Ref		
Endline	1.270	0.10	[0.02,2.52]
***Intervention Impacts*:**			** **
**Comprehensive v Control & Simple**	**0.860**	**0.53**	**[-1.40,3.12]**
***District & Urban/Rural Stratum***			
Urban Choma	Ref		
Rural Choma	-0.527	0.87	[-5.97,4.91]
Urban Livingstone	-1.670	0.43	[-5.16,1.82]
Urban Monze	0.316	0.97	[-13.30,13.93]
Rural Monze	0.429	0.86	[-3.55,4.41]
Average Number of First ANC Visits	0.901	< 0.01	[0.79,1.01]
Distance from DHO	0.011	0.83	[-0.08,0.10]
Constant	0.220	0.93	[-4.15,4.59]

### Impact of interventions on infant DBS testing

The baseline period for the DBS analysis started January 1^st^, 2012 and ended July 31^st^, 2013. During this period, a total of 4,529 DBS samples were collected. Data from August and September 2013 were excluded from the analysis, as this was when the interventions were being piloted.

The average number of infant DBS tests changed by -0.49, 0.43, and 1.19 tests per month over baseline in Control, Simple and Comprehensive Intervention facilities, respectively. There was a 0.92 total difference in average monthly tests for the Simple Intervention facilities compared to control facilities and 1.78 tests for the Comprehensive Intervention compared to Control facilities.

**Fig 2:**
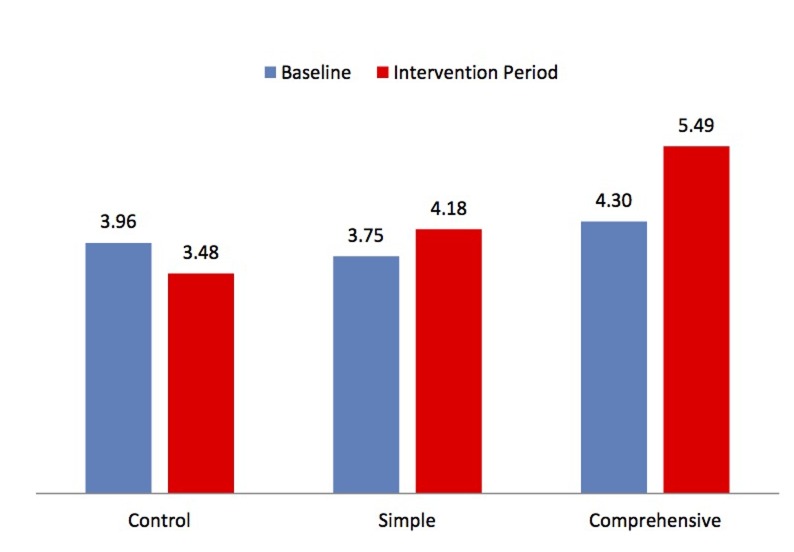
The average number of infant DBS testing during baseline and intervention periods per facility, by intervention. The blue bars represent the average number of infant DBS tests per facility in each study arm at baseline. The red bars represent the corresponding average number of infant DBS tests during the six month intervention period.

According to the multivariate linear regression using a log-transformed outcome, the Simple Intervention resulted in a 16.6% [90% CI: -7%, 46%, P-value = 0.26] greater change in average monthly testing compared to control, and the Comprehensive Intervention resulted in a 10% [90% CI: -10%, 36%, P-value = 0.43] greater change compared to control (Fig 2, Table 4).

**Table 4:** Linear Regression Results of Intervention Arm on Logged Outcome (Number of DBS Tests per Facility per Month).

Covariates	e^B^	P-value	[90% CI]
***Intervention Arm***			
Control	*Ref*		
Simple	0.885	0.32	[0.72,1.08]
Comprehensive	1.005	0.97	[0.83,1.21]
***Time Period***			
Baseline	*Ref*		
Intervention period	0.77	0.10	[0.59,1.00]
***Intervention Impacts*:**			** **
**Simple v Control**	**1.166**	**0.26**	**[0.93,1.46]**
**Comprehensive v Control**	**1.104**	**0.43**	**[0.90,1.36]**
Number of Monthly DPT1 Immunizations	1.006	**0.01**	[1.00,1.01]
***District & Urban/Rural Stratum***			
Urban Choma	*Ref*		
Rural Choma	1.019	0.94	[0.69,1.49]
Urban Livingstone	1.851	**0.05**	[1.12,3.07]
Urban Monze	1.669	0.19	[0.88,3.17]
Rural Monze	1.424	0.13	[0.97,2.08]
Known National Stock out of DBS Kits	0.933	0.49	[0.79,1.10]
Average Number of HIV+ Mothers Anticipated per Month	1.121	**< 0.01**	[1.06,1.19]
Constant	1.093	0.72	[0.73,1.64]

Over time, children were being tested earlier across all facilities, with the average age of a first DBS test dropping from four months to three months of age between January 2012 and January 2014. No significant differences between the study arms were found (data not shown).

### Impact of interventions on maternal re-testing

The baseline period for the maternal HIV retest analysis was from January 1, 2013 to July 31^st^, 2013. In total there were 5,055 postpartum retests done at baseline and 8,712 in the evaluation period, of which 94 were positive (positivity rate of 1.1%).

**Fig 3:**
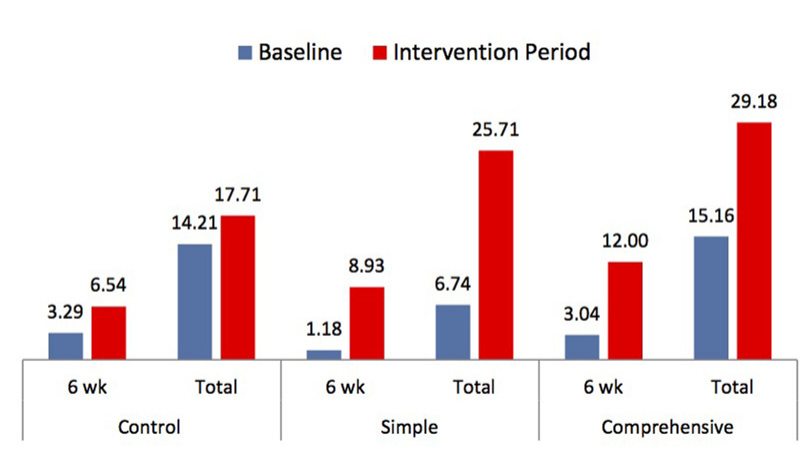
The average number of monthly maternal retests during baseline and intervention per facility, by intervention arm. The bar chart displays the average number of monthly maternal retests both done at the six week under-five visit, as well as the total number of monthly maternal retests during the baseline and the intervention periods across the three intervention arms. The bars in blue represent values at baseline, while bars in red represent values during the six month intervention period.

The average number of monthly maternal HIV tests increased across all facilities (Fig 3). Compared to baseline, there was an average increase of 3.3 (99%), 7.8 (658%) and 9 (295%) 6 week tests and an increase of 3.5 (25%), 19 (281%) and 14 (92%) increase in total retests in control, Simple and Comprehensive Intervention facilities, respectively.

Both interventions resulted in significant increases in the total number of retests (Table 5). According to the multivariate linear regression model, the Simple Intervention resulted in 4.60 additional six-week tests over baseline (90% CI: 2.19, 7.01, P-value< 0.01) compared to the control, and the Comprehensive Intervention resulted in an increase of 5.76 tests (90% CI: 2.63, 8.91, P-value <0.01) compared to the control. The impact of the Simple Intervention on total retests was estimated to be a difference of 15.76 (90% CI: 7.12, 24.41, P-value < 0.01) tests greater than the difference experienced in the Control group. The impact of the Comprehensive Intervention on total retests was estimated to be a difference of 10.93 (90% CI: 1.52, 20.33, P-value = 0.06) tests greater than the difference experienced in the Control group.

**Table 5:** Multivariate Linear Regression Results for 6 Week Retests and Total Number of Retests.

	# of 6 Week Retests			# of Total Retests		
Covariates	Coeff	P-value	[90% CI]	Coeff	P-value	[90% CI]
***Intervention Arm***						
Control	Ref			Ref		
Simple	-2.223	0.02	[-3.77,-0.67]	-7.493	0.12	[-15.33,0.35]
Comprehensive	-0.003	1.00	[-1.92,1.91]	1.555	0.80	[-8.64,11.75]
***Time Period***						
Baseline	Ref			Ref		
Intervention period	5.969	< 0.01	[3.05,8.89]	1.669	0.73	[-6.36,9.69]
***Intervention Impacts*:**						
**Simple v Control**	**4.597**	**< 0.01**	**[2.19,7.01]**	**15.763**	**< 0.01**	**[7.12,24.41]**
**Comprehensive v Control**	**5.769**	**< 0.01**	**[2.63,8.91]**	**10.925**	**0.06**	**[1.52,20.33]**
Number of Monthly DPT1 Immunizations	0.044	0.05	[0.01,0.08]	0.200	0.05	[0.04,0.36]
***District & Urban/Rural Stratum***						
Urban Choma	Ref			Ref		
Rural Choma	0.513	0.77	[-2.38,3.41]	1.479	0.84	[-10.85,13.80]
Urban Livingstone	-4.119	0.01	[-6.55,-1.68]	-6.339	0.31	[-16.62,3.94]
Urban Monze	1.075	0.87	[-9.36,11.51]	19.387	0.52	[-30.33,69.10]
Rural Monze	0.342	0.82	[-2.15,2.83]	2.127	0.69	[-6.80,11.05]
Average Number of 1st ANC Visits per Month	0.098	0.01	[0.04,0.16]	0.323	0.02	[0.09,0.56]
Distance to DHO	-0.041	0.22	[-0.10,0.01]	-0.076	0.44	[-0.24,0.09]
Constant	-3.403	0.33	[-9.16,2.36]	-5.384	0.82	[-44.47,33.70]

### Focus Group Discussions

Sixteen focus group discussions were conducted in the catchment area of eight facilities. Six groups of mothers who had attended outreach services and ten groups of mothers who had attended facility-based services were included.

The majority of women from both groups expressed positive opinions about HIV testing. Women recognized the importance of knowing their status, as that knowledge enabled them to take better care of themselves and of their child. One woman from a facility-based group said, “We want to be retested early so that we know our status rather than waiting until we get too sick.” Another woman from an outreach group indicated, “Others don’t want to be tested, but if you are breastfeeding and retested positive, you are advised on how to take care of your baby and yourself.”

## Discussion

This evaluation provides evidence to support the existing Zambian policy of integration of EPI with EID. Integration of services was feasible, did not result in deleterious effects on immunization, and improved maternal HIV retesting. The study was not designed to detect a difference between the Comprehensive and the Simple Intervention, and a large difference in any of the three primary outcomes was not observed between the intervention groups. This is the first known study that has utilised a randomised approach to examine the impact of the integration of EID and EPI services on rates of vaccination uptake and HIV testing.

### Impact on immunization uptake

Our findings indicate that the interventions did not have a significant impact on DPT1 immunization uptake. Comprehensive Intervention facilities administered an average of 0.86 more doses of DPT1 per month compared to control and Simple Intervention facilities combined. With the lower 90% confidence limit of -1.4 doses, the impact would be less than 5.3% fewer monthly doses. The combined Simple and Comprehensive Intervention facilities had 0.5 fewer DPT1 doses delivered per month compared to the control facilities. With the lower limit of the 90% CI at -3.59 doses per month the impact of the integration of services would be less than a 13% decrease in DPT1 coverage. These estimates are below the 20% threshold that was determined for the a-priori interim analysis to assess for evidence of harm following the integration of services.

Focus group discussions provided additional evidence to support the integration of services. Women generally expressed positive attitudes about testing. No evidence was found to indicate caregivers would be less inclined to attend an under-five clinic to avoid HIV testing.

### Impact on maternal HIV testing

Our findings indicate that both the Comprehensive Intervention and Simple Intervention resulted in a significant increase in retests for mothers, with an average of 4.6 and 5.8 additional six-week tests per month and 15.8 and 11 additional total tests per month for the Simple Intervention and Comprehensive Intervention facilities, respectively. The acceptability of the approach was also supported by the focus group discussions which revealed that many mothers thought about the benefits of HIV testing in terms of their children’s well-being as well as their own health.

The increase in maternal postpartum retests in both the Simple and Comprehensive Intervention groups likely highlights the importance of the restock component of both interventions. In an environment of perpetual under-supply, facility staff may have prioritized antenatal HIV testing (with the higher positivity rate) rather than postpartum testing. By reinforcing the supply of testing commodities, this constraint was removed. This indicates that ensuring a reliable and continuous supply of HIV testing materials may be critical to the success of any PMTCT and EID intervention in a similar setting.

Improved maternal retest rates may also have been due to the systematic approach and consequent ‘normalisation’ of testing services, thus reducing some of the fear and stigma. These results are reinforced by other qualitative studies which have found similar results [13].

### Impact on infant DBS testing

Our findings indicated a small but non-significant increase in the number of DBS tests administered for both the Simple Intervention and Comprehensive Intervention. Given the low maternal positivity rate (1.1%) and the low average number of tests performed per month (due to the low number of HIV-exposed infants) meant that the study was not powered to detect a small increase for this outcome. Post hoc power calculations determined that this evaluation was powered to detect only a very large increase in the number of infant DBS tests (66%).

In addition, it is possible that spill over effects may have caused an under-estimate of the true impact on infant DBS testing. The provision of stock at intervention facilities may have decreased the pressure on emergency orders of DBS kits at the district level. As a result, more of the scarce DBS kits at the district level may have been allocated to control facilities from the district than otherwise would have happened. Also, facility-to-facility transfer of DBS kits may have occurred whereby kits allocated to intervention facilities were ultimately used at control facilities.

### Simple Intervention versus Comprehensive Intervention

Whilst we were not powered to detect a difference between the two intervention arms, both arms showed an increase in maternal re-tests. We are unable to determine the extent to which facilities in the Simple Intervention group actually integrated services following the sensitization meeting. However, it appears that re-supplying testing commodities was a key driver of the impact measured.

### Limitations

There were several limitations of this evaluation. Firstly, the research team supported the intervention sites in integrating services, and it is not clear whether the impact shown in this evaluation would be duplicated if scaled up using routine MOH mechanisms (i.e., the level of support may differ).

Although some intervention facilities did stock out of testing commodities, the risk of stock out was likely to be equal for facilities in both of the intervention arms. In addition, as mentioned above, resupplying intervention facilities directly may have alleviated district-level supply shortages and allowed control facilities to receive more testing supplies than they would have otherwise. As a result, the measured impact of the two interventions on maternal retests and DBS tests conducted may be underestimated.

There are also limitations of the data quality due to the study’s reliance on administrative data. The HIV activity sheet was only used in intervention facilities, limiting our ability to validate the number of retests done in control facilities for these results. The HIV activity sheet would have introduced bias if it was used to fill out the BUPIP data (our primary data source for this outcome). However, discrepancies between the HIV activity sheet and the BUPIP data had a slight tendency toward under-reporting on the BUPIP data. While the inability to verify the postpartum maternal retests in the control facilities may have introduced some bias, it is unlikely that this explains the full effect that was detected, especially given the magnitude of the effect. With this exception, data for all outcomes in the intervention period were validated through other data sources during the intervention period. Such validation was not conducted for the baseline period.

All facilities were supported by the ZCAHRD BUPIP program, which has helped routinize the provision of a broad range of HIV services in these facilities. In addition, the evaluation setting was primarily rural, which was also indicative of the low number of average monthly tests.

### Policy Implications

Our study provides strong evidence to support the integration of HIV testing and EPI services. As a result of this evaluation, Zambia’s MOH has distributed a memo to remind health facilities to provide HIV testing at under-five clinics. In addition, the memo instructs districts to ensure the measurement of performance through the inclusion of under-five HIV testing as part of district performance assessments. This is considered a critical step to scale up the integration of services. In addition, efforts are also being focused on improving the availability of HIV testing commodities through the integration of the EID commodities into the HIV commodities supply chain.

## Conclusion

Taken together, our findings support and extend existing knowledge on the impact of the integration of HIV testing with EPI. We provide the first robust data from a randomised controlled trial that shows integration of services does not have a negative effect on immunization uptake. We support the findings of other studies illustrating an increase in maternal testing and that EID-EPI integration is a feasible and an acceptable approach.

## Supporting Information

S1 Consort Checklist Consort Checklist. (DOCX)

S1 File Analysis Plan. (DOCX)

S1 Protocol Evaluation Protocol Submission for Ethical Review; Lusaka, Zambia. (PDF)

S2 Protocol Evaluation Protocol Submission for Ethical Review; Boston, USA. (DOCX)
