# Supplementary material for: A Cluster Randomised Trial on the Impact of Integrating Early Infant HIV Diagnosis with the Expanded Programme on Immunization on Immunization and HIV Testing Rates in Rural Health Facilities in Southern Zambia
Source: PLoS One. 2015 Oct 29;10(10):e0141455. doi: 10.1371/journal.pone.0141455 (PMC4626083; doi:10.1371/journal.pone.0141455)
Supplement: S1 File — (DOCX) [file pone.0141455.s002.docx]

EID / EPI 3DE Evaluation Analysis Plan

**Evaluation and Analysis Overview**

This analysis plan is for the EID/EPI integration evaluation, part of the 3DE initiative in Zambia. The design is a three armed clustered randomized controlled trial with 20 health facilities in each intervention arm. The arms are detailed below:

1. The **“Control Facilities”** will continue “business as usual” and will only be visited for data collection purposes.
2. The **“Simple Intervention”** targets two potential causes of low Early Infant Diagnosis (EID) rates: supply stock outs and poor understanding of testing requirements and guidelines. Health facilities receiving the Simple Intervention will benefit from 1) a guaranteed supply of antibody and dried blood spot (DBS) testing materials and 2) a short workshop from district MoCDMCH staff to review and emphasize existing MoH EID guidelines.
3. The **“Comprehensive Intervention”** includes the supply and information components of the Simple Intervention, and also introduces 1) an intentional operational optimization and integration of EID testing into routine six-week immunization visits, and 2) an additional component of opt-out rapid HIV testing for all mothers with previously negative or unknown HIV status in order to identify previously unrecognized HIV-exposed infants. Overall goal is to improve HIV services without hurting immunization program rate that is delivered at the same time.

**Analysis Overview**

1. **Health facility data** – all data for the evaluation is administrative data that has been recorded by health facility workers or volunteers at the 60 health facilities.
   1. Data is collected once per month by evaluation team field officers from health facility registers and health facility monthly reports
   2. Data is entered into mobile phones using Open Data Kit
   3. Data for some outcomes can be collected from multiple sources to use for data verification purposes
2. **Note on multiple data sources -** In cases where there is more than one way of measuring an outcome, the pros and cons of different data sources are listed. One data source is listed as the primary data source for the analysis and the other data sources will be used to identify discrepancies for follow-up. In cases where the primary data source is missing and cannot be located with follow-up visits, the secondary data source will be used to validate the primary source and fill in gaps caused by missing or lost data. This will be documented in a separate, commented section of the final analysis .do stata file. For full list of variable names, types, variable codes, and descriptions see the Analysis Plan Codebook (forthcoming).
3. **Summary of analytical approaches**
   1. **Immunization analysis**
      1. **MoH guidelines:** DPT1 immunization delivered at the 6 weeks health visit to infants
      2. **Outcome:** # of DPT1 immunizations delivered, measured at clinic level
      3. **Analysis Approach:** linear regression
   2. **Infant testing analysis (DBS – Dried Blood Spot Testing or EID – Early Infant Diagnosis)**
      1. **MoH guidelines:** DBS tests done at 6 weeks and at 6 months for HIV-exposed infants
      2. **Outcome:** # of DBS tests done at 6 weeks and at 6 months, measured at clinic level
      3. **Analysis Approach:** linear regression
   3. **Mother testing (Rapid Testing)**
      1. **MoH guidelines:** HIV rapid tests done on previous HIV-negative HIV-unknown mothers who bring their infants for under-5 at 6 weeks, 6 months, 9 months, 12 months, and 18 months of age
      2. **Outcome:** % of mothers tested, measured as a rate at the clinic level
         1. Numerator – number of rapid tests done
         2. Denominator – number of infants of relevant age attending under-5 clinic
      3. **Analysis Approach:** Logit regression
   4. **Rate of positive rapid tests**
      1. **MoH guidelines:** Cases where HIV rapid tests (section c) are positive
      2. **Outcome:** % of tests positive
         1. Numerator – number of positive rapid tests
         2. Denominator – number of rapid tests done
      3. **Analysis Approach:** % of tests positive at different ages in each intervention group

**Analysis Plan Section 1: Immunization Uptake**

Introduction:

This analysis will use a randomized difference-in-differences approach with immunization figures aggregated at the clinic level. This will first be used to conduct analysis at the 3 month mark (half way through the evaluation) to determine whether there has been a significant drop in immunizations that warrants altering the evaluation. The same analysis will be replicated at the end of the project.

Goal of Analysis: Measure any drop in immunizations DPT1 between 20 comprehensive facilities and 40 other facilities.

Data sources:

- **Primary data source:** Under-5 Tally Sheets – sheets filled out by health facility staff at time of immunizations
  - **Variables collected:**
    - DPT1 / immunization session – primary outcome variable
    - OPV 1 & PCV1 / immunization session – backup outcome variable in cases where DPT is stocked out
    - Number of infants weighed / immunization session – covariate / data validation variable
  - **Pros:**
    - Updated at time of service delivery
    - Regularly updated
    - Allows for totals from outreach (vaccinations done in communities) and static (vaccinations done at health facility) to be added up separately
  - **Cons:**
    - Only has Under-5 number without any other identifying information
    - Tally sheets are occasionally misplaced by health facility staff
- **Secondary data source:** HIA 2 – monthly report facilities send to district
  - **Variables collected:**
    - DPT1 / month – primary outcome variable back-up / data validation
    - OPV 1 & PCV1 – backup in cases where DPT is stocked out
  - **Pros:**
    - Easily accessible
    - Easy to compare each month to another
    - Can potentially be used to fill in aggregate totals in cases of misplaced forms
    - Can also be used to cross check quality of field officer data collection
  - **Cons:**
    - Only a single number, cannot be disaggregated by person or static / outreach
    - Facilities can be inaccurate at counting tick marks

DPT 1 immunization is the primary immunization indicator.

- **Primary data source:** DPT1
  - Pros: Almost never out of stock, no cases of historical stockouts
  - Cons: None known
- **Secondary data source:** PCV1
  - Pros: Rarely stocks out
  - Cons: Recently introduced (last year), so there are fewer historical records and sometimes appears to be issued to older infants to “catch up” against ministry guidelines
- **Other data source:** OPV1
  - Pros: None
  - Cons: Large stock out problems at beginning of project

Analysis approach:

The immunization regression analysis uses a panel data approach to control for any time effects and take advantage of additional statistical power gained by repeated observations of the same event. To this end, the data is arranged in “long” format with the structure show in Table 3 below. All variables are described in the below section.

**Mock Structure of Panel Data**

| Facility # | month | dpt | Treatment  Group | | Month3 | … | Month36 | Treatment  Month | Other Covariates |
| --- | --- | --- | --- | --- | --- | --- | --- | --- | --- |
| 1 | 1 | 6 | 1 | 0 | | … | 0 | 0 | … |
| … | … | … | … | … | | … | … | … | … |
| 1 | 36 | 9 | 1 | 0 | | … | 1 | 1 | … |
| 2 | 1 | 25 | 0 | 0 | | … | 0 | 0 | … |
| … | … | … | … | … | | … | … | … | … |
| 2 | 36 | 27 | 0 | 0 | | … | 1 | 0 | … |
| … | … | … | … | … | | … | … | … | … |
| 60 | 1 | 112 | 1 | 0 | | … | 0 | 0 | … |
| … | … | … | … | … | | … | … | … | … |
| 60 | 36 | 108 | 1 | 0 | | … | 1 | 1 | … |

The research team will test five different models. All regressions use the standard linear regression command in Stata. Those with separate endline-month covariates use the linear combination command, “lincom”, to test a linear combination of the six end line variables. This command tests for a net intervention effect across the three months of the intervention period (coded “interventionMonth1” through “interventionMonth6”) by combining the coefficients and standard errors to create a joint estimate. All regressions cluster the standard errors at the health facility level using Stata’s cluster option.

The overall approach to the analysis was to start with a basic difference in differences model (Model 1 and II) and then add facility level covariates (Model III) and then test two potential time trends (Model IV and Model V). The difference in differences model controls for both baseline differences between intervention groups (treatmentGroup variable) and end line time trends (variables month34 to month 36) defined more precisely below.

**Variable List**

**avgANC** – the average number of antenatal care first attendance visits by month by facility. This covariate is used as the best estimate of the different maternal and child patient load of each facility and expected number of immunizations per month.

**distanceFromDHO** – the distance, in kilometers, from each health facility to the district health facility. This covariate is used as a proxy for urban / rural divide.

**dpt** – the number of DPT1 immunizations administered by a given facility in a given month. This is the outcome variable.

**facilityCode** – facility code for each health facility in sample.

**interventionTimeTrend** – interaction variable between timeTrend and treatmentGroup variables.

**interventionPeriod =** month34 + month35 + … + month36. One for all endline observations (treatment and control) and 0 for all baseline observations.

**month3 …month36** – dummy variables for each value of time. Month1 and month2 were not included to prevent collinearity with the time variable. Month3 to month33 are baseline months and month34 to month39 are end line months.

**treatmentGroup** – dummy variable for the treatment arm of interest (Comprehensive Intervention)

**treatmentMonth1 =** treat * month34. This equals one for observations in treatment clinics in October.

**treatmentMonth2 =** treat * month35. This equals one for observations in treatment clinics in November.

**…**

**treatmentMonth6 =** treat*month39. This equals one for observations in treatment clinics in March.

**treatmentEndline =** treat*(month34 + month35 + … + 36). This equals one for observations in treatment clinics at any point in the intervention period.

**time** – time variable where month = 1 is the first time period and month = 36 is the last time period.

- Baseline observations are from time = 1 to time = 33 and represent months before the intervention was implemented (pre October 2013)
- End line observations are from time = 34 to time = 39 and represent October 2013 to March 2014.

**timeTrend** – time trend variable for end line variables. TimeTrend = 1 if time = 34, timeTrend = 2 if time = 35, … , and timeTrend = 6 if time = 39

**Model Specifications**

1. **Basic Model I:** No covariates used, control for baseline difference in immunization levels between groups

- dpt = treatmentEndline treatmentGroup interventionPeriod, cluster(facilityCode)
- Treatment effect estimated by the coefficient and p-value of the treatmentEndline variable

1. **Basic Model II:** No covariates used, control for baseline difference in immunization levels between groups and overall time trends in endline time period. This differs from model I in that each month of endline is treated as a separate outcome (rather than part of a pooled outcome). This allows the research team to look for time trends within the endline period

- dpt = treatmentMonth1 … treatmentMonth6 treatmentGroup month34 … month39, cluster(facilityCode)
- Treatment effect estimated as a linear combination: treatmentMonth1 + … + treatmentMonth6

1. **Basic Model + Facility covariates:** Model 2 with facility level variables (avgANC and distanceFromDHO) as covariates

- dpt = treatmentMonth1 treatmentMonth6 treatmentGroup month34 … month39 avgANC distanceFromDHO, cluster(facilityCode)
- Treatment effect estimated as a linear combination: treatmentMonth1 + … + treatmentMonth6

1. **Full Model:** Model 3 + linear time variable

- dpt = treatmentMonth1 … treatmentMonth6 treatmentGroup month34 …month39 avgANC distanceFromDHO time, cluster(facilityCode)
- Treatment effect estimated as a linear combination: treatmentMonth1 + … + treatmentMonth3

1. **Full Model 2:** Model 3 + time dummy variables

- dpt = treatmentMonth1 … treatmentMonth3 treatmentGroup month34 month35 month36 avgANC distanceFromDHO month*, cluster(facilityCode)
- Treatment effect estimated as a linear combination: treatmentMonth1 + … + treatmentMonth6

**Analysis Plan Section 2: DBS Uptake**

Like the immunization analysis above, analysis will follow a randomized difference in difference approach at the clinic level. Again there are multiple data sources to choose from and we will start this section discussing which ones will be used. The analysis for DBS tests is complicated by the fact that each HIV-exposed infant should receive 2 DBS tests (1 at 6 weeks and 1 at 6 months). We will first look at these tests separately and then look at them together.

**Data Sources**

- **Primary source:** DBS Tracking Register
  - **Pros:**
    - Collected in a register so records cannot go missing
    - Typically filled out at time of test
  - **Cons:**
    - Entries may be skipped by poorly run facilities.
- **Secondary source:** DBS Laboratory Requisition Book
  - **Pros:**
    - Needs to be filled out in order for facility to send a sample to lab
    - When records are complete, usually matches DBS tracking register
  - **Cons:**
    - Facilities often run out of pages and make photocopies that are not kept in the book / get lost.
- **Secondary source (to cross check numbers with other sources):** DBS lab data base
  - **Pros:**
    - Measures number of tests that arrive to lab
    - Kept in clean data base in Lusaka
    - Can easily obtain historical records at clinic level
  - **Cons:**
    - Poor data entry for baseline period (new lab tech starting September 2013)
    - Delays in delivery of samples such that not all evaluation samples are processed yet

**Analysis approach**

The data structure and analysis approach for the DBS outcome are very similar to the approach for the immunizations outlined in the section above. The data is arranged in a “long format” with the outcome variable tied to the time period (in this case month) that it is affiliated with. The differences with the immunization analysis are as follows:

- The below regressions are running for the following three outcomes:
  - # of first DBS tests done (~6 weeks of age)
  - # of second DBS tests done (~6months of age)
  - Total # of DBS tests done
- Additional covariates that are relevant specifically to DBS testing may be included
  - Changes to staffing levels during intervention period
  - District level HIV prevalence rates

**Variable List**

The majority of the variables in the below model specifications are the same as those in the immunizations list (pages 4-5) and are not restated here.

**dbs6wks –**the number of first dbs tests administered by a given facility in a given month. This is an outcome variable.

**dbs6mos –** the number of second dbs tests administered by a given facility in a given month. This is an outcome variable.

**dbsTotal** = dbs6wks + dbs6mos – the total number of dbs tests administered by a given facility in a given month. This is an outcome variable

**staffChange –** the number of additional or fewer staff at a facility that month (ie. +1 – one additional staff, -2 – two less staff)

**districtHIV –** the HIV prevalence rate for the district (hard to get at the facility level)

**Model Specifications**

Presented below is the specification for dbsTotal. The model specifications for the other two outcome variables (dbs6wks, dbs6mos) will be identical. The baseline period for dbs data starts in January 2013, which is month25 in the previous data structure. Month25 to month33 is the baseline period and month34 to month 39 is the endline period

1. **Basic Model I:** No covariates used, control for baseline difference in immunization levels between groups

- dbsTotal = treatmentEndline treatmentGroup interventionPeriod, cluster(facilityCode)
- Treatment effect estimated by the coefficient and p-value of the treatment

1. **Basic Model II:** No covariates used, control for baseline difference in immunization levels between groups and overall time trends in endline time period. This differs from model I in that each month of endline is treated as a separate outcome (rather than part of a pooled outcome). This allows the research team to look for time trends within the endline period

- dbsTotal = treatmentMonth1 … treatmentMonth6 treatmentGroup month34 … month39, cluster(facilityCode)
- Treatment effect estimated as a linear combination: treatmentMonth1 + … + treatmentMonth6

1. **Basic Model + Facility covariates:** Model 2 with facility level variables (avgANC, distanceFromDHO, staffChange, districtHIV) as covariates

- dbsTotal = treatmentMonth1 treatmentMonth6 treatmentGroup month34 … month39 avgANC distanceFromDHO staffChange districtHIV, cluster(facilityCode)
- Treatment effect estimated as a linear combination: treatmentMonth1 + … + treatmentMonth6

1. **Full Model:** Model 3 + linear time variable

- dbsTotal = treatmentMonth1 … treatmentMonth6 treatmentGroup month34 …month39 avgANC distanceFromDHO staffChange districtHIV time, cluster(facilityCode)
- Treatment effect estimated as a linear combination: treatmentMonth1 + … + treatmentMonth3

1. **Full Model 2:** Model 3 + time dummy variables

- dbsTotal = treatmentMonth1 … treatmentMonth6 treatmentGroup month25 … month36 avgANC distanceFromDHO staffChange districtHIV, cluster(facilityCode)
- Treatment effect estimated as a linear combination: treatmentMonth1 + … + treatmentMonth6

**Analysis Plan Section 3: HIV Rapid Tests**

Analysis for this section will only be done at the facility using primarily logit regression techniques. The data quality for these outcomes is lower than data in Section 1 and Section 2, although the effect sizes we are attempting to measure are much larger. As a result, the data will be analyzed using different sources to best triangulate the overall result.

**Outcomes:**

- % of 6 week of immunizations client whose mother gets a retest (# of 6 week retests / # of static DPT 1 immunizations)
- % of U5 clients whose mother gets a retest (# of total retests / [# of static DPT1 immunizations * 5]) – Note: Mothers should be tested 5 times in a year and a half: 6 wks, 6 months, 9 months, 12 months, and 18 months
- % of all mother rapid tests that are positive (# of positive retests / # of total mother retests)
- % of mother rapid test that are positive at different ages for infant – 6 weeks, 6 months, 9 months, 12 months, 18 months (# of positive retests / # of total mother retests)

**Data sources:**

- **PRIMARY SOURCE (1 - Intervention)** HIV Activity Sheets
  - **Pros:**
    - Detailed organized information
  - **Cons:**
    - Not used in control facilities (part of the intervention package)
    - Filled out poorly in some health facilities with test results not always recorded although quality and completeness is improving over time
- **Primary Source (2 – Control)** BUPIP Monthly Report Forms
  - **Pros:**
    - Always filled out by all facilities in easy to collect and analyze form
    - Can be compared to HIV activity sheets in the intervention facilities
  - **Cons:**
    - Summarized from poor records in registers that don’t explicitly separate maternal retests from other sources at all facilities
- **Secondary Source** Improvised Registers for Retests
  - Pros:
    - Can be accurate and detailed when used
  - Cons:
    - Not always used
- **Secondary Source** General Counseling Register
  - Pros:
    - Can be accurate and detailed when used for PNC testing
  - Cons:
    - Often does not indicate which retest is being done 6/52, 6/12, 9/12 etc.

**Analysis Approach**

The analysis for rapid tests will 1) compare the retest rates done across intervention arms and 2) examine the positive rates of retests at different ages.

For the first case, the data will be arranged long to capture changes over time that pooled regressions could miss. A logit regression will be used because the outcome variable is a percentage. This analysis will be done with the outcome variable from two different data sources. First, HIV activity sheet data will be used to compare simple and comprehensive intervention arms as this is believed to be higher quality data. Second, BUPIP data will be used to compare control, simple and comprehensive intervention arms. This data is lower quality, but is more widely available. All regressions will be run on data from the 6-week clinic and on data representing the total numbers of rapid tests conducted at under-5 clinic.

For the second case, simple percentages (number of positive tests / number of tests) are calculated with 95% confidence intervals. This is done for the positivity rates for each time period (6 wks, 6 mo, etc) as well as the overall rate.

**Variable List**

Note: the majority of the variables in the below model specifications are the same as those in the immunizations list (pages 4-5) and dbs list (pages 7-8) and are not restated here.

**6wksActivity -** the number of 6 weeks retests tests administered by a given facility in a given month recorded on HIV activity sheets. This is an outcome variable.

**retestsTotalActivity –** the number of all retests tests administered by a given facility in a given month recorded on HIV activity sheets. This is an outcome variable.

**6wksBU -** the number of 6 weeks retests tests administered by a given facility in a given month recorded on HIV activity sheets. This is an outcome variable.

**retestsTotalBU -** the number of all retests tests administered by a given facility in a given month recorded by the BUPIP forms. This is an outcome variable.

**Model Specifications**

Presented below is the specification for TotalBU. The model specifications for the other outcome variables (6wksActivity, retestsTotalActivity, 6wksBU) will be identical. The baseline period for HIV testing data starts in January 2013, which is month25 in the previous data structure. Month25 to month33 is the baseline period and month34 to month 39 is the endline period

1. **Basic Model I:** No covariates used, control for baseline difference in immunization levels between groups

- retestsTotalBU = treatmentEndline treatmentGroup interventionPeriod, cluster(facilityCode)
- Treatment effect estimated by the coefficient and p-value of the treatmentEndline variable

1. **Basic Model II:** No covariates used, control for baseline difference in immunization levels between groups and overall time trends in endline time period. This differs from model I in that each month of endline is treated as a separate outcome (rather than part of a pooled outcome). This allows the research team to look for time trends within the endline period

- retestsTotalBU = treatmentMonth1 … treatmentMonth6 treatmentGroup month34 … month39, cluster(facilityCode)
- Treatment effect estimated as a linear combination: treatmentMonth1 + … + treatmentMonth6

1. **Basic Model + Facility covariates:** Model 2 with facility level variables (avgANC, distanceFromDHO, staffChange, districtHIV) as covariates

- retestsTotalBU = treatmentMonth1 treatmentMonth6 treatmentGroup month34 … month39 avgANC distanceFromDHO staffChange districtHIV, cluster(facilityCode)
- Treatment effect estimated as a linear combination: treatmentMonth1 + … + treatmentMonth6

1. **Full Model:** Model 3 + linear time variable

- retestsTotalBU = treatmentMonth1 … treatmentMonth6 treatmentGroup month34 …month39 avgANC distanceFromDHO staffChange districtHIV time, cluster(facilityCode)
- Treatment effect estimated as a linear combination: treatmentMonth1 + … + treatmentMonth3

1. **Full Model 2:** Model 3 + time dummy variables

- retestsTotalBU = treatmentMonth1 … treatmentMonth6 treatmentGroup month25 … month36 avgANC distanceFromDHO staffChange districtHIV, cluster(facilityCode)
- Treatment effect estimated as a linear combination: treatmentMonth1 + … + treatmentMonth6

IDinsight is consulting with our technical advisors to finalize the list of covariates to include in each of the analyses, and how to specify them. We will consider theory, existing evidence from the literature, and field observation to guide which variables are most important to control for. After finalizing this pre-analysis plan, we could possibly consider alternate specifications, but only if these different specifications do not change the results, but instead increase precision of the impact estimate. In such cases any final reports will note that the specification was not given in the pre-analysis plan.
