# Supplementary material for: A Cluster Randomised Trial on the Impact of Integrating Early Infant HIV Diagnosis with the Expanded Programme on Immunization on Immunization and HIV Testing Rates in Rural Health Facilities in Southern Zambia
Source: PLoS One. 2015 Oct 29;10(10):e0141455. doi: 10.1371/journal.pone.0141455 (PMC4626083; doi:10.1371/journal.pone.0141455)
Supplement: S1 Protocol — (PDF) [file pone.0141455.s003.pdf]

Measuring the impacts of health facility reinforcement and EID and EPI service integration on testing and immunization services in Southern Province, Zambia

CONFIDENTIAL

EVALUATION PROTOCOL SUBMISSION FOR ETHICAL REVIEW

Date: 9th May 2013

Principal Investigator: Paul Wang, Founding Partner, IDinsight - Zambia

Co-Investigators:

- Dr. Max Bweupe, Deputy Director Public Health and Research, Zambia Ministry of Health
- Dr. Pascalina Chanda-Kapata, Principal Surveillance and Research Officer, Zambia Ministry of Health
- Dr. Albert Mwango, National ART Coordinator, Zambia Ministry of Health
- Dr. Penelope Kalesha, Child Health Specialist, Zambia Ministry of Community Development, Mother and Child Health
- Dr. Simon Mutembo, Clinical Care Officer, Provincial Health Office, Southern Province, Zambia Ministry of Health
- Dr. Godfrey Biemba, Country Director, Zambia Centre for Applied Health Research and Development
- Dr. Davidson Hamer, Director of Research and Evaluation, Zambia Centre for Applied Health Research and Development
- Elizabeth McCarthy, Director - Applied Analytics Team, Clinton Health Access Initiative
- Bwalya Kangwa, Program Manager – Demand Driven Evaluations for Decisions, Clinton Health Access Initiative

Executive Summary

Zambia has made significant progress in expanding HIV testing services and treatment within the mother-child "cascade" which includes identifying and treating HIV-positive mothers, HIV-exposed infants and HIV-positive infants. Early infant HIV diagnosis (EID) services have been implemented in over 800 health facilities nationwide and three DNA PCR laboratories have been established in Ndola and Lusaka. However, despite recent progress, there still exist gaps in the identification of HIV-exposed and HIV-infected infants and linkage to care. Zambia's Ministry of Health (MoH) guidelines dictate that known HIV-exposed babies should be tested twice for HIV status after birth – once at six weeks and again at six months using a DNA PCR test. However, although an estimated 80,000-90,000 HIV-positive women give birth in Zambia each year, only 45,000 – 48,000 infant DNA PCR tests (out of the recommended 160,000-180,000 tests) are conducted each year.[iii] Thus, improving EID testing rates represents a critical step in improving Zambia's performance along the mother-child HIV testing and treatment cascade.

Senior officials from Zambia's MoH and Ministry of Community Development, Mother and Child Health (MoCDMCH) have requested this evaluation to assess two interventions designed to improve EID testing rates:

1. The **"Simple Intervention"** targets two potential causes of low EID rates: supply stock outs and poor understanding of testing requirements and guidelines. Health facilities receiving the Simple Intervention will benefit from 1) a guaranteed supply of antibody and dried blood spot (DBS) DNA PCR testing materials and 2) a short workshop from district MoCDMCH staff to review and emphasize existing MoH EID guidelines.

2. The **"Comprehensive Intervention"** includes the supply and information components of the Simple Intervention, and also introduces 1) an intentional operational optimization and integration of EID testing into routine six-week immunization visits, and 2) an additional component of opt-out rapid HIV testing for all mothers with previously negative or unknown HIV status in order to identify previously unrecognized HIV-exposed infants.

A cluster randomized evaluation design – randomized at the health facility level – will be utilized to identify the impact of the Simple and Comprehensive Interventions on HIV and immunization indicators. Relevant baseline and outcome data on the number of HIV antibody tests, DNA PCR tests and immunizations will be collected from the facility registers by trained enumerators. Additionally, exit interviews will be conducted for a sub-sample of women in evaluation health facilities. A secondary outcome of the Comprehensive Intervention is that it will provide an estimate of the incidence of HIV among women who previously tested negative for HIV during pregnancy.

This combination of quantitative and qualitative investigations is designed to provide insight for policy makers into the impact of these activities as well as general attitudes of respondents on the interventions.

While this study is designed primarily to inform Zambia's EID policies and guidelines to improve health and well-being throughout Zambia, it will also contribute to the global knowledge base regarding the integration of HIV services with other popular services and related topics.

Table of Contents

Executive Summary 2
Table of Acronyms 4
CHAPTER 1: BACKGROUND, SIGNIFICANCE, AND OBJECTIVES 5
1.1 Background and Introduction 5
1.2 Study Objectives and Context 5
1.3 Study Significance 6
1.4 Study Aims 7
1.5 Specific Study Objectives 7
CHAPTER 2: LITERATURE REVIEW 9
2.1 Literature Review 9
CHAPTER 3: STUDY DESCRIPTION AND METHODS 11
3.1 Description of Interventions 11
3.2 Study Design and Methods 11
3.3 Data Sources and Validation 17
3.4 Study Period 18
3.5 Outcomes and Expected Results 18
3.6 Ethical Issues 19
3.7 Discussion 20
3.8 Limitations 21
3.9 Dissemination of Findings 21
3.10 Budget 22
CHAPTER 4: APPENDICES 23
4.1A Appendix 1: Information Sheet 23
4.1B Information Sheet – Tonga Translation 23
4.2A Appendix 2: Informed Consent Forms 24
4.2B Informed Consent Form – Tonga Translation 25
4.3 Appendix 3: Authorization Letter to Work in Study Sites 26
4.4 Appendix 4: Study Sample Health Facilities 2633
4.5 Appendix 5: Surveys 34
4.6 Appendix 6: Investigator CVs 48
4.7 Appendix 7: References 98

Table of Acronyms

ANC – Antenatal Care

ART – Antiretroviral Treatment

CHAI – Clinton Health Access Initiative

CHW – Community Health Worker

DBS – Dried Blood Spot

DHO – District Health Office

DMO – District Medical Office

DNA PCR Test – DNA Polymerase Chain Reaction Test for HIV

DPT – Diphtheria, Pertussis (Whooping Cough), and Tetanus Vaccine

EID – Early Infant Diagnosis

EPI – Expanded Programme on Immunization

eMTCT – Eliminating Mother to Child Transmission of HIV

MCH – Mother and Child Health

MoCDMCH – Ministry of Community Development, Mother and Child Health

MoH – Ministry of Health

OPV – Oral Polio Vaccine

PITC – Provider Initiated Testing and Counseling

PMTCT – Preventing Mother to Child Transmission of HIV

PNC – Postnatal Care

SMAG – Safe Motherhood Action Group

U5 – Under-5 Children's Health Appointments

ZCAHRD – Zambia Center for Applied Health Research and Development

3DE – Demand Driven Evaluations for Decisions Initiative

CHAPTER 1: BACKGROUND, SIGNIFICANCE, AND OBJECTIVES

1.1 Background and Introduction

Although progress has been made across Sub-Saharan Africa in increasing access to "prevention of mother to child transmission of HIV" (PMTCT) services, significant challenges remain. In 2011, an estimated 330,000 infants were born with HIV worldwide.[iii] For 21 priority countries in Sub-Saharan Africa, PMTCT service coverage rates were only 61% before pregnancy and 28% during breastfeeding.[iv] Similarly, only 28% of HIV exposed infants received an HIV test within the first two months of life.[v] Zambia is no exception, with only 27.4% of HIV-exposed infants tested within the first two months of life.[vi]

Early identification of HIV-positive infants is critical in improving their chances of survival. Without identification and treatment, it is estimated that over 50% will not survive through their second year.[vii] For HIV-positive infants who are identified early and start treatment before the 12-week mark, up to 75% of these deaths can be avoided.[viii]

Zambia has made strong progress in expanding PMTCT services over the last three years, with 94% of women undergoing HIV testing at Antenatal Care (ANC) in 2010 and 86% of HIV-positive women receiving efficacious antiretroviral for PMTCT in 2011.[ix] Unfortunately, these antenatal PMTCT successes have not carried over into post-natal EID rates. Current MoH guidelines stipulate that each HIV-exposed infant should be tested twice using the DNA PCR test, first at 6 weeks after birth and again at 6 months after birth. Because an estimated 80,000-90,000 HIV-positive women give birth in Zambia each year, 160,000-180,000 DNA PCR tests should be processed annually if MoH guidelines are followed. However, in 2011, only 45,000 DNA PCR tests were processed –25% of the national target.[xxi]

In Zambia, uptake of routine under-five immunizations is high, with immunization coverage for all vaccines estimated to exceed 80%.[xii] Routine immunization, following the recommended World Health Organization (WHO) Expanded Programme on Immunizations (EPI) guidelines, is scheduled at birth, six weeks, ten weeks, fourteen weeks and nine months, with different vaccines administered at each time point. The high coverage of DTP1 vaccinations, estimated at 87% in 2011, suggests that the 6-week visit is particularly well attended by mother-baby pairs. These high immunization rates have prompted Zambian health officials to explore the possibility of utilizing routine immunization as an opportunity to boost Zambia's EID rates.

Zambia's MoH and international partners have invested in improving the infrastructure and systems necessary to increase EID testing services in recent years. EID is now available in over 800 health facilities in all 10 provinces. There are currently two DNA PCR labs in Lusaka, one in Ndola, and additional labs scheduled to open in Chipata and Livingstone. The Zambia Centre for Applied Health Research and Development (ZCAHRD), the implementing partner for this evaluation, is also scaling up an SMS-based test results reporting platform that halves the time required to return EID test results to health facilities. Once operational, these additional DNA PCR labs and SMS services will reduce the transport burden for time-sensitive lab results and increase the nation's overall HIV testing capacity. Given Zambia's increasing HIV laboratory infrastructure, it is an appropriate moment to explore opportunities to increase the nation's overall EID testing rates.

1.2 Study Objectives and Context

The primary objectives of the study are to assess the impact of:

1. Study interventions on EID testing rates of HIV-exposed infants
2. Study interventions on the number of women identified to be HIV-positive
3. The Comprehensive Intervention on infant immunization uptake

Secondary objective include:

1. Estimating the incidence of HIV among women who previously tested negative for HIV during pregnancy
2. Evaluating the cost-effectiveness of the Simple Intervention versus the Comprehensive Intervention.

The primary goal of this study is to evaluate different measures that might improve EID testing rates and identification of HIV-positive mothers and babies without negatively affecting uptake of immunization services. Identifying such measures has recently become highly relevant due to the Zambian government's recent announcement to support a strategy of universal HIV testing and implementation of Option B+ nationwide as a strategy to eliminate mother-to-child transmission of HIV.[1] [xiii] Critical to the success of Option B+ is the need to make sure that a high percentage of women are tested for HIV and placed on treatment if discovered to be HIV-positive. For this reason, the study intervention is also designed to increase the number of new mothers that are tested for HIV.

MoH officials have identified routine under-five immunizations in health facilities as an opportunity to boost EID activities. The routine immunization schedule – where infants are scheduled to attend health facilities at six weeks, ten weeks, fourteen weeks, and nine months – aligns well with the DBS testing schedule. Ideally, DBS tests should be conducted at six weeks after birth. Then the results should be transported to the laboratory, analyzed, and returned to the health facility three to six weeks later. This time frame allows subsequent immunization visits to be used as times to communicate test results and refer for treatment if necessary.

Finally, WHO guidelines recommend that all HIV-exposed infants should receive cotrimoxazole prophylaxis, an antimicrobial agent, 4-6 weeks after birth, and continue that treatment until HIV infection can be excluded.[xiv] As part of Zambia's minimum HIV-exposed infant care and treatment package, providers are trained to initiate HIV-exposed infants on cotrimoxazole prophylaxis from 6 weeks of age onwards. However, Zambia's MoH 2010 PMTCT report stated that only 40% of HIV-exposed infants were being properly initiated on cotrimoxazole prophylaxis. Possible reasons for this include: poor recording and documentation of the prophylaxis, lack of proper identification of HIV-exposed infants, and frequent stock outs of the drug. Integrating HIV services more closely with routine immunizations represents an additional opportunity to identify many more HIV-exposed infants and initiate them on cotrimoxazole prophylaxis.[2]

1.3 Study Significance

This evaluation will be the second evaluation launched under the Demand Driven Evaluations for Decisions (3DE) initiative, a three-year partnership between the Clinton Health Access Initiative (CHAI), IDinsight, Zambia's MoH, and Zambia's MoCDMCH. By using rigorous impact evaluations in a demand-driven and efficient manner, 3DE seeks to generate reliable impact evidence to catalyze at-scale implementation of cost-effective policies.

The evaluation will help Zambia's MoH and MoCDMCH determine if the Simple Intervention or Comprehensive Intervention can cost-effectively improve HIV testing rates and the identification of HIV-positive and HIV-exposed mothers and babies without harming under-five immunization uptake. Furthermore, the evaluation will illuminate the operational requirements of these activities, including requirements for funding, staff, training, communication, equipment and logistics. The results of this evaluation will inform the potential scale up of either intervention as well as broad policy decisions on the possibility of integrating HIV services more closely with other existing patient touch points.

---

1 Option B+ is a single, universal ART regimen given to all HIV-infected pregnant or breastfeeding women for life, in hopes of simplifying service delivery and reducing mother-to-child transmission of HIV in the current and future pregnancies.

Finally, the results will contribute new findings to a limited global evidence base. There are few studies that have measured the impact on immunizations caused by the integration of HIV services despite the fact that several countries are moving towards this approach. Likewise, there are few studies that have examined the benefits of another round of universal HIV testing of infants to identify more HIV+ mothers and HIV-exposed babies.

1.4 Study Aims

The aims of the study are described below:

1. Estimate the impact of both the Simple Intervention and Comprehensive Intervention on the following HIV-related outcomes:
    a. Percentage of mothers with unknown or previously negative HIV status tested for HIV-infection (antibody test)[2]
    b. Percentage of HIV-positive women newly identified[3]
    c. Percentage of HIV-exposed infants tested for HIV status (DNA PCR test)[4]

2. Estimate the impacts of the Comprehensive Intervention on the following immunization-related outcomes:
    a. Percentage[5] of infants receiving first-visit immunizations
    b. Percentage of infants receiving second-visit immunizations[6]

3. Assess the cost, staffing and testing requirements of the Simple Intervention and Comprehensive Intervention.

1.5 Specific Study Objectives

Specific research question	Key activities
What is the impact of the Simple and Comprehensive Interventions on HIV testing parameters?	Compare program and comparison sites on the following measures:<br>• Total number of mother-infant pairs tested for HIV-infection (antibody test)<br>• Total number of infants tested for HIV status (DNA PCR test)<br>• Total number of HIV-positive women identified
What is the impact of the intervention on the percentage of infants receiving first-visit and second-visit EPI immunizations?	Compare program and comparison sites on the percentage of infants receiving DTP1, OPV1, DPT2 and OPV2
What is the cost of implementing the Simple and Comprehensive Interventions?	Build a costing model to examine the cost-benefit of implementing both interventions

[2] Denominator is the number of mothers with unknown or prior negative HIV status presenting at the health facility for their infant's routine six week immunizations
[3] Denominator is the number of women presenting at the health facility for their infant's routine six week immunizations
[4] Denominator is the number of known HIV-exposed infants presenting at the health facility for routine six week immunizations
[5] For each health facility, the denominator for the percentage calculation will be # of pregnant women attending ANC. This figure can subsequently be converted to an estimate of # of women giving birth in a given health facilities catchment area. Importantly, the primary indicator is the *percentage change* of the indicator – thus, this metric will be usable even in the rare / unlikely case that the # infants immunized exceed the # infants presenting at least once for ANC.
[6] First immunizations refer to the 6-week visit for under-five health facility where children receive the following vaccinations: OPV1 and DPT1-HepB-HiB. The Second immunization refers to the 10-week visit to under-five health facility for OPV2 and DPT2 vaccines.

What staff capacity is required at the facility level for the Simple and Comprehensive Interventions?<br><br>What HIV testing capacity is required to scale the Simple and Comprehensive Interventions across Zambia?	relative to improved morbidity and mortality<br><br>Build a model to assess the staff and HIV test capacity required to implement the intervention nation-wide

CHAPTER 2: LITERATURE REVIEW

2.1 Literature Review

Research indicates that integrating universal opt-out mother and infant HIV testing with routine immunizations could be an effective approach to improve mother and infant diagnosis. However, this specific intervention has never been evaluated using an experimental design, and could fill a number of gaps remaining in the global literature. Thus, the proposed evaluation has the potential to make important contributions to both Zambia's policy landscape and the global knowledge base on HIV testing and health service integration at the point of service delivery.

Below is a discussion on related research on routine HIV testing and health service integration.

Integrating Health Services with Immunizations

Wallace et al (2012A) conducted a systematic literature review on the integration of immunization services with other maternal and child health services. They found that integration had a positive impact on the uptake of all newly integrated services, although the coverage rates of non-immunization services did not achieve the same high rate of coverage as immunization. The study identified the use of immunization visits as a possible venue for HIV testing to evaluate the performance of PMTCT services. Important barriers to integration mentioned included adding additional time to the visit length for each mother.[xv]

Partapuri et al (2012) conducted a literature review on the possibility of integrating additional maternal and child services into immunization outreach campaigns. Interventions included in the review were: ANC, deworming, growth monitoring, bed nets, Integrated Management of Childhood Illness (IMCI), nutrition, and hygiene. No HIV related interventions were included in the review. The reviewers found that integration is most effective when interventions can be feasibly integrated at the outreach level, coordination can be conducted at all program levels, joint training and supervision of health workers and programs is conducted, community based organizations are engaged, and monitoring and evaluation systems provide timely feedback. The researchers focused on the importance in this setting of strong engagement with community health volunteers in promoting the benefits of integration. [xvi]

HIV Testing at Time of Immunizations

Rollins et al. (2009) ran a study at three health facilities in KwaZulu Natal, South Africa, where all mothers who brought infants in for immunizations were also offered HIV testing of infants. Infants were first tested for the presence of HIV antibodies to determine HIV exposure, and if antibodies were present, then they were tested for HIV DNA to determine HIV infection. They found that universal HIV infant testing at immunization health facilities was feasible to identify and refer HIV-infected infants. Of the 646 mothers who brought their infants for immunizations, 90.4% agreed to the HIV testing and 56.8% of those mothers returned for results.[xvii]

Sinunu et al. (2011) ran a study in Malawi to determine the mother-to-child transmission rate in the country ahead of the adoption of Option B+. The research team utilized a random selection process to select a sample of mother-infant pairs attending immunizations to test for HIV-exposure. They found that 9.8% of mother's tested positive who had never tested before, 6.9% of women who tested negative before pregnancy were now HIV-positive, and 4% of women who tested negative since pregnancy were now HIV-positive. The overall prevalence rate for mothers was 14.4% with 8.4% of positive mothers passing HIV onto their infant.[xviii]

Provider Initiated Testing and Counseling (PITC)

A study in Lilongwe, Malawi, used a pre- post comparison to find that implementation of provider-initiated HIV testing and counseling, and integration of ART services in the pediatric ward, was associated with an

increase in uptake of testing services and number of patients initiated on ART. The proportion of children and adults initiating ART each quarter increased from 26% to 53%, and 20% to 52%, respectively.[xix]

In Zambia, a study at primary care outpatient health facilities in Lusaka, Zambia, found that introducing routine PITC significantly increased the uptake and acceptability of HIV testing. After the addition of PITC, the nine health facilities in the sample group tested over twice as many patients as before. Over time, the percentage of individuals who accepted testing rose, indicating that introducing PITC helped to decrease the stigma surrounding HIV testing.[xx]

Medical Benefits of Early Identification and Treatments of HIV-Positive Children

A randomized trial conducted by the National Institutes of Health found benefits to early infant initiation of ART, instead of waiting to initiate treatment until disease symptoms are seen. This is especially important since HIV disease progression occurs more rapidly in infants than in adults. In the study, early identification of HIV status and early initiation of antiretroviral therapy reduced early infant mortality by 76%.[xxi]

A study of infected and uninfected infants born to HIV-positive mothers found that babies that were identified early and introduced to treatment had significantly better mortality outcomes than those who did not immediately go on treatment. At 1 year of age, 35.2% of infected infants died without treatment and 52.5% had died by 2 years of age without treatment.[xxii]

Contribution of Evaluation to Literature

This proposed study will contribute to the literature in several important ways. First, it will assess the extent to which integration of universal HIV testing with routine under-five immunizations, similar to current practice with ANC, can boost HIV testing rates without negatively impacting uptake of immunization. Secondly, it would inform Zambia and other high HIV prevalence countries whether either intervention could be a cost effective approach to identifying new HIV positive infants and mothers. Finally, this study will provide insight into the feasibility of integrating HIV into other public health services.

CHAPTER 3: STUDY DESCRIPTION AND METHODS

3.1 Description of Interventions

This section describes the two interventions that will be tested in this evaluation, hereafter referred to as the "Simple Intervention" and the "Comprehensive Intervention."

The **Simple Intervention** targets two potential causes of low EID rates: (1) supply stock outs and (2) poor understanding of existing MoH testing requirements and guidelines. This intervention will have two components:

1. **Supply Reinforcement** – The research team will work with facility level staff to ensure that orders for necessary HIV testing supplies are placed on time and in sufficient quantity. In the event of a generalized stock out of HIV testing supplies at the province or district level, the evaluation team will provide facilities with an additional outside supply to allow them to continue testing operations.
2. **Guidelines Reinforcement** – The research team will arrange facility visits by district health office officials to meet with relevant health staff, remind them of exiting MoH HIV testing guidelines, and remind them that improving EID testing is a Ministry priority.

The **Comprehensive Intervention** integrates universal, opt-out HIV screening and Early Infant Diagnosis of HIV (EID) services with Expanded Program on Immunization (EPI) Services in Zambia. This intervention will include:

1. Simultaneous provision of services to a mother-infant pair by health care workers without interruption between services
2. Opt out testing of mothers with unknown or prior negative status

In addition, the Comprehensive Intervention will—in an identical manner to the Simple Intervention—reinforce the supply of HIV testing equipment at facility level and reinforce guidelines among health professionals.

3.2 Study Design and Methods

This study is designed to measure the impact of the Simple Intervention and Comprehensive Intervention on the number of women and infants tested and identified as HIV-positive as well as the percentage uptake of certain immunizations. Statistical power calculations are based on the immunization uptake outcome variable, as this is the indicator with the smallest effect size required to be policy-relevant. The study will test the following null hypotheses:

1) Infants are not less likely to attend immunization clinics for DPT1, OPV1, DPT2 and OPV2 immunizations in Comprehensive Intervention health facilities versus the combination of the Simple Intervention and Comparison health facilities

2) Rapid HIV antibody tests are not more likely to be administered in:
   1. Simple Intervention facilities versus Comparison facilities
   2. Comprehensive Intervention facilities versus Simple Intervention facilities

3) DNA PCR tests are not more likely to be administered in:
   1. Simple Intervention facilities versus Comparison facilities
   2. Comprehensive Intervention facilities versus Simple Intervention facilities

4) Mothers are not more likely to be identified as HIV-positive in:

1. Simple Intervention facilities versus Comparison facilities
2. Comprehensive Intervention facilities versus Simple Intervention facilities

The study will be designed as a cluster randomized controlled trial randomized at the health facility level. Program and comparison health facilities will be selected via random selection methodology, to ensure that the only differences between the samples in the three groups are the components of the intervention.

Sample frame
This study will take place in the Southern Province of Zambia in health facilities supported by ZCAHRD that are providing PMTCT and EID services at facility level. The sample frame comprises of 83 health facilities in Choma, Livingstone, and Monze districts. These districts were selected out the 10 districts in Southern Province based on leadership, geographic dispersion, urban / rural characteristics, current HIV prevalence rates, and absence of conflicting research projects. Hospitals and facilities without adequate skilled staff were excluded from the sample frame. The selection of Southern Province was made in conjunction with MoH officials who communicated that lessons learned from an evaluation in Southern Province could be generalized to other provinces in Zambia for purposes of scale up.

Study sample
It has been determined that a sample of 60 facilities (20 facilities for each evaluation group) – selected out of the sample frame of 75 facilities – is required for the study. The sample will be stratified by an urban/rural divide and district, with a third of the sites randomly chosen as comparison facilities, a third randomly chosen as Simple Intervention facilities, and the final third randomly chosen as Comprehensive Intervention facilities. Thus, the unit of randomization will be the health facility with all infants attending the given health facility for immunizations during the period of the evaluation will be the cluster. Random assignment and post randomization checks will ensure that on average, observable and unobservable characteristics of the program and comparison groups are statistically similar. Observable characteristics that will be checked for balance across evaluation arms include baseline HIV testing rates, baseline immunization rates, number of ART sites, and catchment population sizes. In doing so, any difference in outcomes between program and comparison group can be causally attributed to the intervention.

The power calculation for each outcome is outlined below:

Effect of Comprehensive Intervention on immunization rates
- **Level of outcome:** DPT 1 immunization
- **Sample:**
  - o 20 Comprehensive Intervention facilities compared to 40 Simple Intervention and Comparison facilities
  - o 98 new infants per facility
- **Detectable percentage point decrease in immunization uptake:** 10%
- **95% plausibility interval:** Expected probability that an infant receives DPT1 immunization is 87% with bounds around this estimate from 55 – 95%
- **Significance level:** 5%
- **Power:** 86%[7]

Effect of Simple Intervention on rapid HIV antibody testing rates (Simple versus Comparison facilities)
- **Level of outcome:** Rapid HIV antibody tests administered
- **Sample:**
  - o 20 Simple Intervention facilities compared to 20 Comparison facilities
  - o 98 new infants per facility

[7] Analysis will include the use of historical immunization data as covariates which will increase power to a level greater than 80%. For the sample frame, at least 60 to 70% of the variation in immunization rates on a month to month basis can be explained by the previous year of data.

- **Detectable percentage point increase in antibody tests:** 5%
- **95% plausibility interval:** Expected probability that a mother/infant pair receives a rapid HIV antibody test is 5% with bounds around this estimate from 1 - 10%
- **Significance level:** 5%
- **Power:** 87%

Effect of Comprehensive Intervention on rapid HIV antibody testing rates (Comprehensive versus Simple facilities)

- **Level of outcome:** Rapid HIV antibody tests administered
- **Sample:**
  - 20 Comprehensive Intervention facilities compared to 20 Simple Intervention facilities.
  - 98 new infants per facility
- **Detectable percentage point increase in antibody tests:** 20%
- **95% plausibility interval:** Expected probability that a mother/infant pair receives a rapid HIV antibody test is 15% with bounds around this estimate from 1 - 30%[8]
- **Significance level:** 5%
- **Power:** 93%

Effect of Simple Intervention on DBS PCR testing rates (Simple versus Comparison facilities)

- **Level of outcome:** DBS PCR tests administered
- **Sample:**
  - 20 Simple Intervention facilities compared to 20 Comparison facilities
  - 98 new infants per facility
- **Detectable percentage point increase in DBS PCR tests:** 4%
- **95% plausibility interval:** Expected probability that an infant pair receives a DBS PCR test is 5%[9] with bounds around this estimate from 1 - 10%
- **Significance level:** 5%
- **Power:** 75%[10]

Effect of Comprehensive Intervention on DBS PCR test rates (Comprehensive versus Simple facilities)

- **Level of outcome:** DBS PCR tests administered
- **Sample:**
  - 20 Comprehensive Intervention facilities compared to 20 Simple Intervention facilities
  - 98 new infants per facility
- **Detectable percentage point increase in DBS PCR tests:** 4%
- **95% plausibility interval:** Expected probability that a mother/infant pair receives a DBS PCR test is 9% with bounds around this estimate from 5 - 14%[11]
- **Significance level:** 5%
- **Power:** 82%

Effect of Simple Intervention on percentage of new mothers identified as HIV-positive (Simple versus Comparison facilities)

- **Level of outcome:** HIV-positive mothers identified
- **Sample:**

---

[8] This assumes that the simple intervention will have the effect assumed in the previous power calculation because this calculation compares comprehensive clinics to simple clinics.

[9] This assumes that of the approximately 45,000 babies tested currently for a DNA PCR, around two-thirds are first test PCR (done at around the 6-week time frame) and the remaining 1/3 are retests. This means that 30,000 out 600,000 babies born each year receive a PCR test at 6 weeks - around 5%.

[10] As in the case of the immunization data, covariates will be used to increase power for this outcome

[11] This assumes that the simple intervention will have the effect assumed in the previous power calculation because this calculation compares comprehensive clinics to simple clinics.

    - 20 Simple Intervention facilities compared to 20 Comparison facilities.
    - 98 mother / infants pairs per facility
- **Detectable percentage point increase in HIV-positive mothers identified:** 0.8%
- **95% plausibility interval:** Expected probability that a mother is identified as HIV-positive is 0.2% with bounds around this estimate from 0.1 – 1.0%
- **Significance level:** 5%
- **Power:** 76%

Effect of Comprehensive Intervention on percentage of new mothers identified as HIV-positive (Comprehensive versus Simple facilities)

- **Level of outcome:** HIV-positive mothers identified
- **Sample:**
    - 20 Comprehensive Intervention facilities compared to 20 Simple Intervention facilities
    - 98 mother/infant pairs per facility
- **Detectable percentage point increase in HIV-positive mothers identified:** 1.5%
- **95% plausibility interval:** Expected probability that a mother is identified as HIV-positive is 1.0% with bounds around this estimate from 0.5% – 2.0%
- **Significance level:** 5%
- **Power:** 87%

These power calculations represent lower bounds, as covariate analysis will further increase the actual power of the evaluation. We can assume that because all research questions are sufficiently powered to detect effects between either the comprehensive and simple facilities or the simple facilities and the comparison facilities, all research questions are sufficiently powered to evaluate effects between the comprehensive and comparison clinics in the case where the simple clinics show no effect.

Intervention implementation

The implementation of the Simple Intervention and the Comprehensive Intervention will consist of health staff training, engagement with district health officials and medical commodity suppliers, intervention communication, and data collection.

All facilities will receive basic training on proper patient documentation across the different registers used to record EID, EPI, and U5 visit information

Simple Intervention implementation

- **Testing supply reinforcement:** The research team will work with health facility staff to review the order history of EID testing supply and ensure sufficient stocks are ordered in a timely fashion through normal MoH supply chains. The research team will also provide contact information to the district health office and health facility in case there is a broad stock-out of EID testing supplies. If this occurs, the research team will arrange the delivery of testing supplies to program facilities affected by stock outs from a separately purchased buffer stock for the purposes of the evaluation. Leftover testing supplies will be donated to the participating district offices at the conclusion of the evaluation. Medical Stores Limited will be informed that some facilities selected for the evaluation may be requesting increased testing supplies for the 6 months of the evaluation. The testing supplies reinforcement will be closely coordinated with MoH to ensure that proper MoH procedures are followed.

- **Guidelines reinforcement:** The research team will work with district medical offices to reinforce testing guidelines through the following activities:

- Organizing a meeting between facility staff and district DMO or district MCH coordinators that emphasizes the importance of adhering to testing guidelines, reminds the staff of all times tests should be done, and emphasizes that improving EID testing rates are a ministry priority.
- Introducing a month-by-month tracking sheet with annual targets for number of EID tests conducted, similar to sheets currently used for immunizations, ANC visits, and in-facility deliveries.

Comprehensive Intervention Implementation
All components of the Simple Intervention will also be implemented in the Comprehensive Intervention health facilities with the following additions:

- **Communication of the intervention:** The research team will provide health facility staff with a brief message describing aspects of the Comprehensive Intervention to be relayed to mothers at different facility touch points. These touch points will include ANC appointments, Safe Motherhood Action Group outreach (SMAG), in-facility delivery, and post-delivery baby registration at post-natal care visits. Additionally, at six week immunization visits, mothers and caregivers will receive group counseling on opt-out HIV screening service and the importance of regular HIV screening for mother and child health.

- **Implementation of universal, opt-out screening of HIV**: The research team will hold a consultation meeting with each Comprehensive Intervention health facility and a representative from the District Health Office to discuss the updated testing algorithm, which proceeds as follows:

    - The health facility worker will examine the HIV status on the U5 or ANC card for all infants attending their first U5 visit

        - If marked **Confirmed Exposed (CE)**, the health care worker will conduct only the DNA PCR test on the baby.

        - If marked, **Mother Status Unknown (MSU)** or **Confirmed Not Exposed (CNE)** the mother or caregiver will be asked if the mother has ever tested HIV-positive. If yes, the health care worker will conduct the DNA PCR on the baby. If no, the mother will be offered an HIV antibody test in an opt-out manner to assess her HIV status. All normal antibody testing procedures will be followed using the Determinate Antibody Tests and Unigold confirmatory test along with all standard counseling messaging. All babies of mothers who test HIV-positive will then receive a DNA PCR test.

- **Operations optimization and integration of testing services:** The research team will work with the district staff and facility to optimize the patient flow at under-five facilities to include new testing procedures and more closely integrate EID and EPI services. The overall integration structure utilizes the six week immunization visit to identify HIV-exposed babies and conduct DBS testing for exposed babies and the ten week or fourteen week immunization visit to deliver DBS test results. At the six week immunization visit, integration will consist of optimization of staff tasks, patient flow, and data collection processes to deliver EID and EPI services in alignment with MoH guidelines. Such operational optimization will enable EID services to be integrated with EPI services in a way that does not adversely affect staff workloads and patient waiting times.

Piloting the Comprehensive Intervention
All Comprehensive Intervention health facilities will pilot this approach for two weeks under the supervision of the research team and project enumerators to troubleshoot difficulties before data collection begins.

Analysis
Impacts of the Simple and Comprehensive Interventions on EID testing rates, HIV-positive mothers and infants identified, and immunization data will be estimated by fitting multilevel linear probability and logit[12] regressions that compare outcomes between program and comparison facilities while controlling for important covariates, such as historical EID and EPI rates, district, catchment population, facility staff, geography and patient characteristics. Data analysis will be conducted using Stata (version 12).

The at-scale costs of both inventions will be modeled using external inputs as well as cost estimates from the evaluation intervention. Moreover, operational best practices of this intervention will be assessed to determine the feasibility of scaling up either set of intervention nationwide.

Technical study risks and potential biases
The discussion below describes the approach towards potential research design effects that could pose threats to the integrity of the results of this evaluation.

Important steps will be taken to minimize the risk of Hawthorne effects, the process by which a subject of a study changes their behavior due to the knowledge that they are being surveyed and measured. For this evaluation, this is principally a problem for the EID and HIV testing outcomes of interest. Health facility staff may put extra effort into HIV testing in response to this project in addition to the intervention. To reduce this risk, the research team will work through Ministry of Health channels and District health officials as much as possible to more closely mimic typical managerial attention. This will better approximate Ministry oversight which will continue after the evaluation as compared to a pure research project. Analysis will also be done to see how consistent testing rates are across the duration of the experiment. The outcomes relevant to immunization uptake should not be affected by Hawthorne effects as they center on patient, rather than facility staff, behavior.

The study analysis will account for the risk of spillovers affecting the results of immunization uptake outcome variable between program and comparison facilities. Only one-third of facilities in the evaluation will be Comprehensive Intervention facilities which serve as the program group for the immunization uptake variable whereas the remaining two-thirds of the facilities will serve as a comparison group for this variable. There is a risk that, if the communication for the EID/EPI integration intervention is not properly targeted, mothers in comparison catchment areas may also receive the message. To minimize this risk, all messages will be highly associated to specific facilities via ANC, SMAGs, in-facility delivery, and PNC touch points, which are all facility-specific channels. However, the potential for word of mouth spread of information and communication spillovers remains. Therefore, the research team will analyze the immunization rates in the comparison sites against historical immunization data for these sites for significant changes over time. Finally, exit interviews will be conducted with comparison and program facility officials to check for observed changes in immunization attendance or rumors they may have heard from community members during the evaluation.

An additional spillover risk exists whereby mothers that ordinarily attend Comprehensive Intervention health facilities switch to other health facilities as a result of the Comprehensive Intervention. The study may collect immunization data from a subset of patients from health facilities neighboring Comprehensive Intervention facilities to estimate the magnitude of such potential shifts. Study sampling that maximizes the distance between study facilities will reduce the likelihood that comparison facility immunization rates are artificially increased due to new patients. Comprehensive Intervention facilities may still experience

[12] A logit regression utilizes a model designed for binary variables that uses the logistic function to ensure values between zero and one

artificially low immunization rates due to transferring patients. Because estimating the proportion of patients that switch facilities that would remain at their typical catchment facility if the Comprehensive Intervention were universally implemented at every health facility would be extremely difficult, it is most appropriate to consider low immunization rates due to transferring patients as an impact of the Comprehensive Intervention although this could overestimate the effect the Comprehensive Intervention would have in a scaled-up environment.

There is a low risk of John Henry effect, the process in an evaluation by which the comparison group realizes that it is a comparison group and changes its behavior in response. Patients are unlikely to realize they are in comparison catchment areas and change their behavior in any way other than outlined in the spillover section. There exists a risk that comparison facilities could take steps to improve EID services that they would not have done in the absence of the evaluation. The research team will attempt to measure this by comparing EID testing rates from before the intervention with testing rates during the evaluation.

There is a low risk of any survey effects for this evaluation. No surveys with durations of over 30 minutes at a time are expected. Because the main outcomes are directly observed, not asked of the respondents, any survey effects are likely to be minimal.

Site Attrition
All sites included in the randomization will have appropriate infrastructure and staffing levels to participate in the study. Thus, it is unlikely that facilities will drop out from the study due to inability to implement the intervention. Because unexpected changes to staffing levels may still lead to attrition, additional facilities have been included in the sample size calculations to ensure adequate statistical power in the event of such occurrence.

Patient attrition
Patient-level attrition could manifest itself due to incorrect facility register recordings. This will be minimized through training and regular monitoring of facility health staff on proper register data collection. Respondent attrition is possible for those who do not consent to exit interviews. However, these activities are not central to the core analysis.

Bounds analysis will be used to account for any attrition in the ultimate analysis.

Impact heterogeneity
Facility registers provide important baseline data on HIV testing, EID, and EPI services and other patient characteristics. Additional baseline information on facility staff, equipment and buildings will be collected. This information will allow the study team to calculate whether the intervention had a differential impact across important variables.

3.3 Data Sources and Validation

The primary source of study data will be the following facility registers: PMTCT Register, Safe Motherhood Register, U5 Register, Exposed Infant Register, DBS Register and any tracking sheets used as intermediaries for these registers. These registers include data on the patient name, ID#'s (Safe Motherhood Number and other tracking IDs), HIV testing dates (Antibody and DBS tests), Immunization provision dates, and DBS results provision dates. To establish facility level HIV testing and immunization rates, historical data from Quarter 1 of 2012 through Quarter 1 of 2013 will be collected, as well as register data recorded during the evaluation period of this research project.

The accuracy of facility register data will be validated by surveying up to 10% of patients in their communities to confirm key information, such as name, date of facility visit, facility name, and key facility

activities. If the patient is in a program facility catchment area, a limited number of additional questions on service delivery experience, patient wait time, and HIV stigma may be asked.

To further verify facility records the research team will also collect data from the DNA PCR lab at the University Teaching Hospital in Lusaka on DBS samples submitted by facility to cross check facility-based records. Likewise, data collected from the "Project Mwana" system, a SMS-based system used by the MoH to deliver DNA PCR results to health facilities, will be used to cross check facility-based records. Finally, up to six villages/neighborhoods per study facility will be selected to survey every woman in the village who delivered a baby in the past six months. Village surveys will occur in parallel with the evaluation activities, and local chiefs and headmen will be contacted to coordinate this activity.   With each mother, a short survey will be conducted to assess:

- Which women attended their 6 week EPI immunization visit in a facility
- What services women attending their 6 week EPI immunization visit in a facility received
- Information from their child's under-five card

This information will be cross-verified with clinic records to further assess completeness and accuracy of facility records.

Finally, focus group discussions with facility staff and communities may be conducted to shed light on perceptions and attitudes towards facility services and interventions. See Annex 5 for English and Tonga versions of each of these surveys and focus groups questions.

3.4 Study Period

This study will take place between May 2012 and January 2013. All data collected for this analysis will come from this study period. Relevant MoH and MoCDMCH officials will be updated on a monthly basis by the research team.

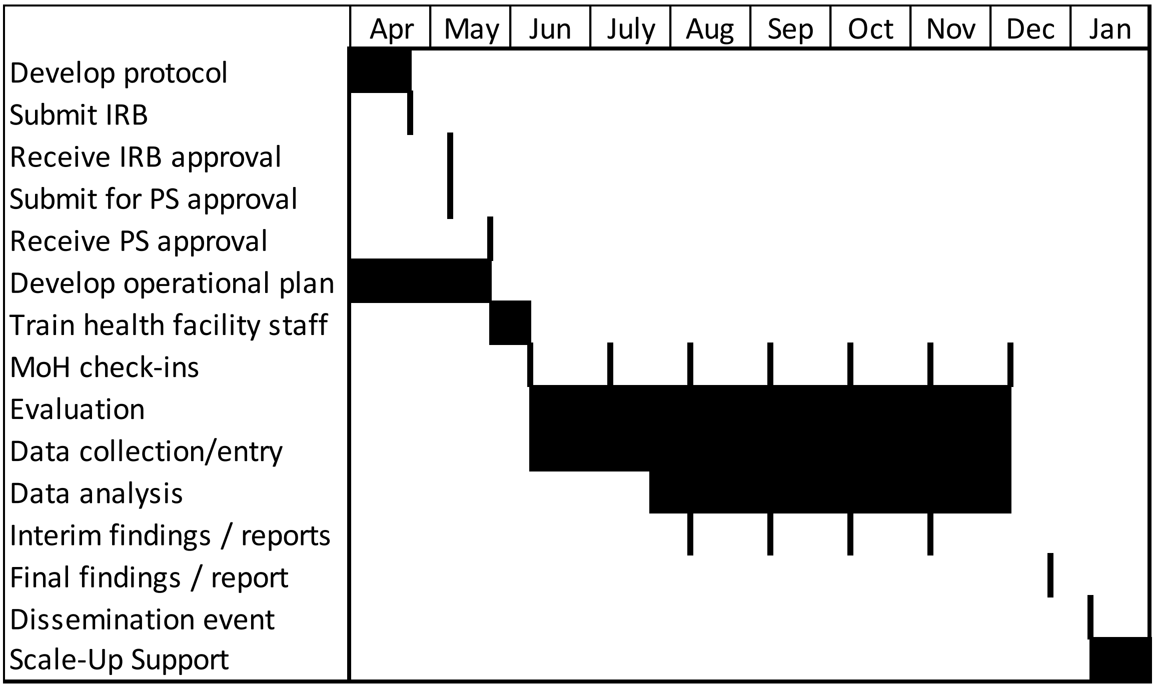

3.5 Outcomes and Expected Results

The primary outcomes of the research questions are:

1. *HIV service delivery rates:* the difference in the following indicators will be compared between the comparison and program groups
    a. Percentage of Mother-infant pairs tested for HIV exposure (antibody test)
    b. Percentage of infants tested for HIV status (DNA PCR test)
    c. Percentage of HIV-positive women identified
2. *Immunization rates:* the difference in the percentage of infants receiving first-visit and second-visit immunizations will be compared between the Comprehensive Intervention groups and the other two evaluation groups.
3. Operational considerations
    a. Assess the cost, staffing and testing requirements of the Simple and Comprehensive Interventions per additional HIV-positive infant and mother identified
    b. Project at-scale cost of the interventions that show a positive impact

3.6 Ethical Issues

We will seek approval from the ERES Converge Ethics Review Board in Lusaka, Zambia and the Boston University IRB. Study staff will comply with the relevant proposal submission policies of ERES Converge in order to gain full ethical approval to conduct the proposed study. After ethical clearance, the proposal will also be submitted to MoH for final permission to conduct the study.

Risks to participants

The proposed evaluation is likely to expose individuals to a minimal level of risk. This section will split risks considered into three sections: risks involving patient attendance, risks involving patient's health facility experience, and risks involving the management of personal data.

Risks involving patient attendance to under-five health facilities are restricted to the Comprehensive Intervention evaluation group. This evaluation will be measuring whether fewer mothers will attend immunization services due to stigma issues surrounding HIV-testing or inconvenience caused by longer appointment times. This represents one of the primary outcomes measured in the study and thus will be monitored very closely. To minimize any potential adverse effects to uptake of routine immunizations, the Comprehensive Intervention will be discontinued if at any time after two months of data collection Comprehensive Intervention facilities are confirmed to have an immunization rate more than 20 percentage points lower than comparison and Simple Intervention Facilities.[13]

For Comprehensive Intervention facilities, the patient experience for mothers will change as a result of the administration of the rapid HIV antibody test for mothers not already confirmed to be HIV-positive. This change in testing procedure will be communicated to mothers during the group counseling session so they are fully informed if they would like to opt-out from the HIV testing. Health facility staff will conduct standard counseling protocols if a mother does allow the rapid HIV antibody test to be conducted. The counseling protocol will be done in private, and contain information about the implications of the test and further options. In this way, mothers will be fully informed throughout of the HIV testing procedures and possibilities during the facility visit.

Risks involving data management and patient privacy apply to all study facilities. All test strips and dried blood samples will only be identified with an identification number, ensuring the privacy of all patients. All Ministry of Health HIV testing guidelines will be complied with, and all data will be collected by trained enumerators using paper surveys or electronic devices. Hard copy surveys will be kept in a locked storage

[13] A linear regression measuring the effect of the Comprehensive Intervention on immunization uptake will be used to assess any decrease in immunizations. "Confirmed" indicates a drop of 20 percentage points is observed at a 95% confidence level.

room accessible only by study staff. All electronic data will be split into two separate files. Names and all other identifying information will be in one file and remaining data with unique individual identifications numbers will be in the second file. These files will be stored on two locked computers with password protected hard drives to ensure the confidentiality of the data. All data collected via electronic device by the enumerators will be transferred as soon as logistics permit to the research manager's hard drive and subsequently erased from the enumerator's digital device. Only 3DE staff conducting the study will have access to identifiable data. The Principal Investigator, Paul Wang, and Research manager, Ben Brockman, will use STATA (version 12) to analyze the data. Primary data analysis will include quality control checks, such as checks for missing data and data entry checks. Data analysis will take place in Zambia.

Potential benefits of the proposed evaluation

The evaluation will directly benefit mother-infant pairs who are newly identified as HIV-positive or HIV-exposed during the study period and referred for treatment. If the interventions evaluated are found to be cost-effective, at-scale implementation will increase the number of mothers and infants identified as HIV-positive and the number of mothers and infants referred for treatment. This would be expected to have a positive impact on infant mortality and life expectancy. An increased identification of HIV-positive mothers and infant exposure risk will allow for more women to be put on lifesaving antiretroviral treatment and will contribute to the elimination of mother-to-child transmission of HIV. Selection of health facilities for the program and comparison groups will be done in an equitable, randomized manner. An additional likely outcome is that HIV-related stigmas could be reduced since HIV-positive mothers will no longer be specifically selected for extra activities.

Informed Consent

Informed consent will be sought from all study participants before any survey is implemented for study purposes. For any such activity, health facility and study staff will read aloud to participants the attached informed consent statement, which is in English and has been translated into the relevant local languages. The consent statement introduces the broad concept being studied, assures that confidentiality will be maintained, and makes clear that participants have a choice about whether to participate and that they may withdraw at any time. Informed consent will not be sought to copy routinely collected patient data recorded on health facility registers or for additional testing during under-five facility visits. In line with the 2008 Zambia Ministry of Health Guidelines on PMTCT, women who are offered opt-out HIV testing do not need to sign an informed consent form; they are only required to be fully informed of the test.[xxiii]

3.7 Discussion

Zambia's Ministry of Health (MoH) leadership has expressed strong interest in increasing EID testing rates and in evaluating the integration of HIV services more closely with U5 services. As previously noted, several countries in Sub-Saharan Africa have formally or informally moved to integrate EID testing in under five health facility visits. Additionally, similar approaches have been the subject of research projects in Tanzania and South Africa; however, neither study was randomized to rigorously evaluate the effect on immunization rates or other important outcomes. This evaluation will fill a significant gap in knowledge in Zambia and in the broader literature on an important topic.

This randomized controlled trial will assess the impact of the Simple Intervention and Comprehensive Intervention on HIV testing rates, HIV-positive identification rates, and immunization uptake rates. The evaluation will provide MoH and MoCDMCH leadership with rigorous evidence to decide whether either set of interventions is worth scaling up and will provide additional policy-relevant insights in several related fields: 1) the feasibility of more closely integrating HIV services with other health services, 2) best practices and resources required to integrate HIV services with other health services and 3) the current efficacy of the PMTCT cascade in a high-prevalence rate of the country. The research questions and design of this study have been developed through consultation with key stakeholders in MoCDMCH, MoH, CHAI, IDinsight, and

BU/ZCAHRD to ensure the highest level of rigor and policy relevance. All relevant partners will be informed of study results.

3.8 Limitations

It is unlikely that this study's population will be perfectly statistically representative of the potential scale-up population. This evaluation focuses on rural, peri-urban, and urban areas of Choma, Livingstone, and Monze districts in the Southern Province of Zambia. Statistical power calculations have been conducted to determine effect sizes across the overall sample. Therefore, the research team may only be able to draw indicative results on differences in some of the outcomes variables in urban areas as compared to rural areas. The study will be conducted in medium to high HIV prevalence areas that may differ from low prevalence areas.

The study will only test one approach to reinforcing testing guidelines and one approach to integrating services more closely. It is possible that other approaches to these broad objectives could have different impacts on the outcome variables of interest.

This study may estimate spillover effects due to patients transferring from Comprehensive Intervention to comparison facilities but will not attempt to estimate the proportion of transferred patients that would have remained at their original facility if the Comprehensive Intervention were universally implemented in all facilities. As a result, impacts on immunization rates are likely to be overestimated, and impacts on HIV-related indicators likely to be underestimated. Both of these biases are in the conservative direction. This is deemed acceptable given the policy-focus of this evaluation and that any study conclusions should account for the worst possible policy scenario – which the approach to spillovers should enable.

Finally, due to logistical considerations, the study does not attempt to integrate EID services with outreach EPI services, where health staff members travel to communities to administer routine under-five immunizations. Likewise, this will shade the impacts on immunization rates and HIV-related indicators conservatively, which is ideal to draw policy implications from the study.

3.9 Dissemination of Findings

The outcomes and analysis, along with a final recommendation, will be compiled into a study report and policy brief for the MoH and MoCDMCH. If this intervention is scaled up across the country, CHAI and IDinsight will continue to work with the partners to monitor the project's status. Any findings from this longer-term monitoring period will be added to the policy brief. Contingent on MoH and MoCDMCH approval, findings will be shared with the Zambian and global health community to learn how EID testing rates can be increased and how HIV services can be better integrated with immunization services.

3.10 Budget

Evaluation Budget (KR)	
**Items**	**Amount**
**Field Staff & transport**	
Field Staff salary & per diem	278,000
Transport	180,000
**Field Staff & transport subtotal**	**458,000**
**Intervention**	
HIV testing backup supply	46,000
Training / engaging health facilities	88,000
**Intervention subtotal**	**134,000**
**Research**	
Field staff training	10,000
ERES ethics review	2,625
Data collection instruments	9,500
**Research subtotal**	**22,125**
**GRAND TOTAL (KR)**	**614,125**

CHAPTER 4: APPENDICES

4.1A Appendix 1: Information Sheet

For participants to take away

You are invited to participate in a study to improve health facility services and reduce HIV-related mother and infant deaths in the community. Participation in this project is completely voluntary. This survey will ask you general questions about your use of medical services during pregnancy and the first year of life of your child or children.

The health services you receive will not be affected by whether or not you decide to participate in the study. You have been chosen to take part in this study because, as a Zambian new mother living in this community, we feel that your experiences may be valuable to our study. You may stop your participation in the study at any time.

You may stop me at any time to ask questions about participation or to stop your participation completely.

Your participation in this study will be confidential. Your name and other identifying information will be accessible only to the researchers and will never appear in any sort of report that might be published. You will not be paid for your participation.

You may contact the Principal Investigator, Paul Wang of IDinsight at 0973 588277 with any problems or questions you may have. You may also contact the Secretary of the ERES Ethics Committee at 0955 155633 or at 33 Joseph Mwilwa Road, Rhodespark, Lusaka.

4.1B Information Sheet – Tonga Translation

For participants to take away

Mwatambwa kuchibbadela kubandika malwazi agumya Sikalileke, Kutegwa tuchesye lufu kubatumbu abana mukabunga. Kujanika muchiyo echi kulyaba.

Muzobuzigwa Mibuzyoyalugwasyo omupegwakuchibbadela chiindi mwanoli ada na nseba. Amwanaakwanisya mwaka omwe.

Munakutambula lugwasyo mwanobandika kuchibbadela. Mwanakubanika kunyina kusungilizya pe. Mwasalwa kuti tubandike andinywe nkaambo muli muchisi cha Zambia luhibo lyenu lulagwasa kabangu aka.

Inga mwandilekezya humbwa chiindi kubuzya mibuzyo olo kulekelalimwi

Mubandi wenu mukabunga aka unoli wamaseseke. Izina iyanu, anzotubandika zilahibwa biyo andiswe. Takumubandi nouyotolwa antangalala pe. Tatukomupa mali iliyonse akubandika andimwe

Inga mwatuma kuli Principal Investigator, Paul Wang of IDinsight ali 0973 588277 namula amapenzi olo mibuzyo. Inga mwatuma kuli ba secretary baku ERES Ethics Committee ali 0955 155633 olo ku 33 Joseph Mwilwa Road, Rhodespark, Lusaka

4.2A Appendix 2: Informed Consent Forms

Informed Consent Form (English)

You are invited to participate in a study to improve health facility services and reduce HIV-related mother and infant deaths in the community. Participation in this project is completely voluntary. This survey will ask you general questions about your use of medical services during pregnancy and the first year of life of your child or children.

The health services you receive will not be affected whether or not you decide to participate in the study. You have been chosen to take part in this study because, as a Zambian new mother living in this community, we feel that your experiences may be valuable to our study. You may stop your participation in the study at any time.

You may stop me at any time to ask questions about participation or to stop your participation completely.

Your participation in this study will be confidential. Your name and other identifying information will be accessible only to the researchers and will never appear in any sort of report that might be published. You will not be paid for your participation.

You may contact the Principal Investigator, Paul Wang of IDinsight at 0973 588277 with any problems or questions you may have. You may also contact the Secretary of the ERES Ethics Committee at 0955 155633 or at 33 Joseph Mwilwa Road, Rhodespark, Lusaka.

Do you have any other questions?

If you think I have addressed all your questions about this project, please tell me, do you agree to participate?

Yes [ ]

No [ ]

Signature (written or thumb print): _________________________

Date: ________________

4.2B Informed Consent Form - Tonga

Mwatambwa kuchibbadela kubandika malwazi agumya Sikalileke, Kutegwa tuchesye lufu kubatumbu abana mukabunga. Kujanika muchiyo echi kulyaba.

Munakutambula lugwasyo mwanobandika kuchibbadela. Mwanakubanika kunyina kusungilizya pe. Mwasalwa kuti tubandike andinywe nkaambo muli muchisi cha Zambia luhibo lyenu lulagwasa kabangu aka.

Inga mwandilekezya humbwa chiindi kubuzya mibuzyo olo kulekelalimwi

Mubandi wenu mukabunga aka unoli wamaseseke. Izina iyanu, anzotubandika zilahibwa biyo andiswe. Takumubandi nouyotolwa antangalala pe. Tatukomupa mali iliyonse akubandika andimwe

Inga mwatuma kuli Principal Investigator, Paul Wang of IDinsight ali 0973 588277 namula amapenzi olo mibuzyo. Inga mwatuma kuli ba secretary baku ERES Ethics Committee ali 0955 155633 olo ku 33 Joseph Mwilwa Road, Rhodespark, Lusaka

Kuli mabuzyo ngomu gisi?

Na muyeya kuti ndawhila mabuzyo yenu ya kabunga aka, mundambila na mwazumina kuti tubandike andimwe?

Inhya [ ] pepe [ ]

Kamusaina / Mudinde awa: ____

Kamusaina olo Mudinde awa: ____

4.3 Appendix 3: Authorization Letter to Work in Study Sites

All Correspondence should be addressed to the Permanent Secretary
Telephone: +260 211 253040/5
Fax : +260 211 253344

In reply please quote:

No.............................

REPUBLIC OF ZAMBIA

MINISTRY OF HEALTH

NDEKE HOUSE
P. O. BOX 30205
LUSAKA

MH/101/17/6

5th September 2012

TO WHOM IT MAY CONCERN

RE: Demand-Driven Evaluations for Decisions (3DE) Initiative - Introduction

Reference is made to the above subject matter.

I am writing to introduce to you the Demand-Driven Evaluations for Decisions (3DE) initiative aiming to support the Government of the Republic of Zambia with evidence-based decision making in the health sector by using rigorous impact evaluations in a demand-driven, rapid and efficient way. The Clinton Health Access Initiative (CHAI) and IDinsight are partnering to pilot the 3DE Initiative with the Ministry of Health. The 3DE initiative offers the opportunity to generate reliable evidence that responds to national health priorities and needs, and enhance the use of research findings for policy and decision making.

The Ministry of Health with the support of the 3DE team is in the process of specifying relevant and useful impact evaluation questions in alignment with the Priority Research Question List 2011-2013. I encourage you to engage with the 3DE team to discuss and refine questions and ideas about potential impactful intervention that may be appropriate for evaluation through this initiative.

For more information about the 3DE initiative, please see the attached introduction note. Tom Pellens, 3DE senior research associate, will reach out to you to start conversations about possible collaboration. Please contact the Director Public Health and Research for any clarifications.

Yours Sincerely,

Dr. P. Mwaba
PERMANENT SECRETARY

Telephone: (260) 211 235341
Fax (260) 211 235342

In reply please quote:

No.:..............................
MCDMCH/14/15/5

REPUBLIC OF ZAMBIA

MINISTRY OF COMMUNITY DEVELOPMENT, MOTHER AND CHILD HEALTH

3rd December, 2012

OFFICE OF THE PERMANENT SECRETARY
COMMUNITY HOUSE
SADZU ROAD
PRIVATE BAG W 252
LUSAKA

To ....

....

....

Dear Sir/Madam,

RE: Introduction of Demand-Driven Evaluations for Decisions (3DE) Initiative – support for field visits

The Ministry of Community Development, Mother and Child Health in collaboration with the Clinton Health Access Initiative (CHAI) and IDinsight are in the process of implementing the Demand-Driven Evaluations for Decisions (3DE) program. The 3DE program aims to support the Government of the Republic of Zambia with evidence-based decision making in the health sector by using rigorous impact evaluations in a demand-driven, rapid and efficient way. The 3DE programme offers the opportunity to generate reliable evidence that responds to national health priorities and needs, and enhance the use of research findings for policy and decision making.

The team is in the process of specifying relevant and useful impact evaluation questions as well as innovative interventions that can improve the cost effectiveness of health care delivery. In light of this, the 3DE team will visit some districts in order to fully understand the MNCH programmes.

Please provide the 3DE team with the necessary support and cooperation during their visits.

Sincerely Yours,

Professor Elwyn Chomba
Permanent Secretary
MINISTRY ORF COMMU NITY DEVELOPMENT, MOTHER AND CHILD

CC: Permanent Secretary, Ministry of Health

All correspondence should be addressed

to the Provincial Health Director

Telephone 324435/323516
Fax: 323391

In reply please quote:

No.

REPUBLIC OF ZAMBIA

MINISTRY OF HEALTH

PROVINCIAL HEALTH OFFICE
P.O BOX 60206
LIVINGSTONE

11th February, 2013.

The District Medical Officers-Livingstone, Choma,
Mazabuka and Monze

RE: <u>INTRODUCTION OF DEMAND-DRIVEN EVALUATIONS FOR DECISIONS (3DE) INITIATIVE-SUPPORTS FOR FIELD VISITS</u>

I refer to the above subject matter.

The Ministry of Community Development, Mother and Child Health in collaboration with the Clinton Health Access Initiative (CHAI) and IDinsight are in the process of implementing the Demand- Driven Evaluations for Decisions (3DE) program aims to support the Government of the Republic of Zambia with evidence-based decision making in the health sector by using rigorous impact evaluations in a demand-driven, rapid and efficient way. The 3DE programme offers the opportunity to generate reliable evidence that responds to national health priorities and needs, and enhance the use of research findings for policy and decision making.

The team is in the process of specifying relevant and useful impact evaluation questions as well as innovative interventions that can improve the cost effectiveness of health care delivery. In light of this, the 3DE team will visit your districts in order to fully understand the MNCH programmes.

Please provide the 3DE team with the necessary support and cooperation during their visits.

Yours faithfully,

Dr. L. Alisheke,
Provincial Medical Officer
SOUTHERN PROVINCE

All correspondences should be addressed
to the District Community Medical Officer
Tel: 03-220370/220532
Fax: 03-220324
Email: chomadho@yahoo.com

In reply please quote
No...........................

REPUBLIC OF ZAMBIA

MINISTRY OF COMMUNITY DEVELOPMENT MOTHER & CHILD HEALTH

Office of the District Community Medical Officer
Choma District Health Management Team
P. O. BOX 630741
CHOMA

TO: ALL HEALTH CENTRE INCHARGES

FROM: DISTRICT COMMUNITY MEDICAL OFFICER

DATE: 6TH MARCH, 2013.

RE: **INTRODUCTION OF DEMAND –DRIVEN EVALUATIONS FOR DECISIONS (3DE) INITIATIVE- SUPPORT FOR FILED VISITS.**

The above mentioned subject matter refers.

This serves to introduce to you the team of Field Evaluators as per attached letters from Ministry of Health and Ministry of Community Development Mother & Child Health.

Please work with them accordingly and assist with data requested.

Attached also is a questionaire.

Do not hesitate to get in touch with DCMO in case of any questions.

Yours faithfully,
Choma District Community Medical Office

Dr. R.F. Mkandawire
DISTRICT COMMUNITY MEDICAL OFFICER

facilityCode	district	facilityName
801010	Choma	Batoka
801032	Choma	Chilalantambo
801099	Choma	Choma HAHC
801051	Choma	Chuumbabeenzu
801052	Choma	Demu
801048	Choma	Harmony
801013	Choma	Jembo
801014	Choma	Kamwanu
801015	Choma	Kanchomba
801044	Choma	Kasikili
801016	Choma	Kasiya
801040	Choma	Kazimaulu
801041	Choma	Macha HAHC
801019	Choma	Mangunza
801020	Choma	Mapanza
801021	Choma	Masuku Mission
801037	Choma	Masuku Terminal
801022	Choma	Mbabala
801023	Choma	Mochipapa
801024	Choma	Moyo
801025	Choma	Muzoka
801034	Choma	Nakeempa
801039	Choma	Nalube
801017	Choma	Ndondi
801035	Choma	Njase
801054	Choma	Pangwe
801026	Choma	Pemba Main
801036	Choma	Pemba Sub
801027	Choma	Popota
801011	Choma	Prisons Choma
801038	Choma	Railway Surgery Choma
801028	Choma	Shampande
801047	Choma	Siamuleya
801045	Choma	Sibanyati
801030	Choma	Sikalongo
801043	Choma	Simakutu
801029	Choma	Simaubi
801049	Choma	Simukanka

Livingstone District Health Office

INTERNAL MEMO

To : CENTRE INCHARGES

From : DCMO

Date : 26/03/13

SUBJECT : EID / EPI RESEARCH

Kindly assist our partners from CHAI and IDinsight with their study. They are assisting the Government on a study to assess the Demand Driven Evaluations for Decisions ((3DE) Initiative. This will assist our Ministry to come up with evidence based decision making.

REPUBLIC OF ZAMBIA MINISTRY OF HEALTH DISTRICT HEALTH 26 MAR 2013 DISTRICT MEDICAL OFFICER P.O. BOX 60796 LIVINGSTONE

G. Sibusenga
SCCO
for/**DISTRICT MEDICAL OFFICER**

All correspondence should be addressed to
The District Medical Officer
032-5011/250734/250724/250798
Fax: 032-50700
Email: monzedho@Zamtel.zm

In reply please quote
No......................

REPUBLIC OF ZAMBIA
MINISTRY OF COMMUNITY DEVELOPMENT, MOTHER AND CHILD HEALTH

Monze District Community Medical Office
P.O. Box 660144,
MONZE

19th March 2013

The Health Centre in charge

- ALL PMTCT SITES

Dear Sir/Madam

RE: INTRODUCTORY LETTER – 3DE RESEARCH TEAM

Please allow the 3DF research team to meet with your facility staff and collect basic data.

The 3DE team is working with the Ministry of Health MCH to conduct policy relevant research.

Your usual cooperation will highly be appreciated.

Yours faithfully

DR. SIALUBANJE C.
DISTRICT MEDICAL OFFICER

4.4 Appendix 4: Study Sample Health Facilities

The study sub-sample will be selected from the below facilities based on distance from the district health office, urban/rural location, population, and baseline immunization rates.

Choma:

Chilalantambo, Chuumbabeenzu, Demu, Harmony, Jembo, Kamwanu, Kanchomba, Kasikili, Kasiya, Kazimaulu, Mangunza, Mapanza, Masuku Mission, Masuku Terminal, Mbabala, Mochipapa, Moyo, Muzoka, Nakeempa, Nalube, Ndondi, Njase, Pangwe, Pemba Main, Pemba Sub, Popota, Prisons, Railway Surgery, Shampande, Siamuleya, Sibanyati, Sikalongo, Simakutu, Simaubi, Simooya, Simukanka, ZNS

Livingstone:

Airport, Boma, Dalice, Dambwa North, Hillcrest, Kasiya, Libes, Libuyu, Linda, Mahatma Ghandi, Maramba, Mosi Oa Tunya, Namatama, Police, Prisons, Victoria Falls

Monze:

Banakaila, Bweengwa, Charles Lwanga, Chisekesi, Hakunkula, Hamangaba, Hamapande, Kanundwa, Katimba, Kaumba, Keemba, Luyaba, Malundu, Manungu, Monze Urban, Moomba, Moonzwe, Nadongo, Nampeyo, Njola Mwanza, Nteme, Rusangu, Siatontola, St Marys, ZCA

4.5 Appendix 5: Surveys

This appendix includes 4 surveys:

Focus Group #1: Mothers who have recently attended Under-5 clinics

Focus Group #2: Health Facility Workers Participating in the Evaluation

Interview Scripts for Exit Interviews for Mothers Done at Health Facilities

Interview Script for Data Verification for Mothers Done in Local Communities

Focus group participants will be appropriately compensated for their participation.

Note: All survey portions that will be conducted in Tonga have been translated and included below.

Focus Group #1: Mothers who have recently attended Under-5 clinics

These focus groups will be conducted in Tonga.

Focus Group Leader: ________________ Date: _________________ Location: ____________

Estimated Time of Session: 30 to 45 minutes

Instructions: Ask the group the following list of questions, noting their responses in the open space. If the same response is expressed multiple times, note the frequency of the given response.

What are your thoughts about under-five clinic visits? Why do you attend Under-five clinic visits for your infant? What services do you think are most important for your infant to receive?<br><br>Sena muyeye buti ali chipimo chabana? Ninzi chomuinkila kuchipimo cha mwana wenu? Ndugwasilizyo buti lupati ndomuyeya mwana wenu inga wa kutambula?
Think about your most recent under-five clinic visit. How was the overall clinic experience? What services did you receive?<br><br>Amuyeye lweendo lwa ino ino lwa ku chipimo? Ino lwakali buti? ndugwasyo buti ndomwa tambulwa?

Were there HIV services discussed or offered at that Under-5 clinic visit? If so, which services? How do you feel about HIV testing and other services being offered at Under-5 clinic visits?<br><br>Ino kwakali ihyakwaamba ihya maluwazi ya sikalike? Kuti yakaliko, ino hyabuti? ino Munvwa buti amakani ya sikalileke ku chipimo?
What is done well at under-5 clinics? What could be improved?<br><br>Chiinzi cho ba chita kabotu ku chipimo? Chiinzi chobanga ba botya?

2) Focus Group #2: Health Facility Workers Participating in the Evaluation (Comparison, Simple Intervention, and Comprehensive Intervention Arms). Some questions will only be relevant for health care professionals that were in certain arms of the evaluation, which is specified after each question.

These focus groups will be conducted in English.

Focus Group Leader: ________________ Date: _________________ Location: _________________

Estimated Time of Session: 30 to 45 minutes

Instructions: Ask the group the following list of questions, noting their responses in the open space. If the same response is expressed multiple times, note the frequency of the given response.

1) What are the biggest challenges you face providing under-5 clinic services? How could these be fixed? (All clinic arms)
2) What are the biggest challenges in delivering HIV-services at under-5 clinic? How could these be fixed? (All clinic arms)
3) What are the most popular services among mothers who bring their children to under-5? Which do they care the least about? (All clinic arms)

4) How do mothers react to the offering of HIV-services at under-5? Do they react different at under-5 clinics compared to other times you interact with them such as ANC, regular health visits, etc.?
5) This project added a few items to under-5 clinics, which of the following did you think were useful? Which were not useful? Why?<br><br>Guarantee of test-kit supplies and ordering support<br>DBS Monthly Target Tracking Sheet<br>Updated Under-5 Tally Sheets<br>Meeting with DHO official on improving HIV testing services at under-5 clinics<br>(Only for simple and comprehensive intervention arms)
6) This project also adjusted the testing algorithm for mothers and infants at under-5 clinics. How well do you think this worked? How much extra work was created? How much time did this add to each under-5 session you completed? (Only comprehensive intervention facilities)

7) This project also changed the clinic flow used for under-5 clinics. How well do you think this worked? Did these changes allow you to do your job better or worse? If you had the choice, would you continue with these changes or return to how it was previously organized? (Only comprehensive intervention facilities)

3) Interview Scripts for Exit Interviews for Mothers Done at Health Facilities

English version. These interviews will be conducted in Tonga

SECTION 1: SURVEY SETUP AND DATA ENTRY		
Before starting the interviw, fill out the information below.		
1.1	District Name:	
1.2	Health Facility Name	
1.3	Date of Interview (DD/MM/YYYY):	|___|___|/|___|___|/|___|___|___|___|
1.4	Time Start Interview (24-hr clock; HH:MM):	|___|___|:|___|___|
1.5	Time End Interview (24-hr clock; HH:MM):	|___|___|:|___|___|
1.6	Smartphone – GPS Coordinates (Decimals)	N / S |___|___|.|___|___|___|___|___|___|___|___| E/W |___|___|.|___|___|___|___|___|___|___|___|
1.7	Surveyor name:	
1.8	Surveyor ID:	|___|___|___|___|
1.9	Respondent Unique ID	|___|___|___|___|
At the time of data entry, please complete the information below.		
1.1	Data Entry Person Name:	
1.11	Data Entry Person ID:	
Read the respondent the informed consent form.		
1.12	Consent Given?	[1] Yes [0] No *End survey*
1.13	Full Respondent Family and Given Name:	
1.14	Unique ID from randomized list	|___|___|___|___|___|___|
1.15	Contact telephone number	

SECTION 2: PERCEPTIONS OF IMMUNIZATION VISIT		
2.1	What services did you and your infant expect to receive at the U5 visit today? **(Do not prompt with list)**	____ Nutrition Counseling<br>____ Vitamin A<br>____ Immunizations<br>____ Family Planning<br>____ HIV Services<br>____________________ Other
2.2	What services did you and your infant receive today? **(Do not prompt with list)**	____ Nutrition Counseling<br>____ Vitamin A<br>____ Immunizations<br>____ Family Planning<br>____ HIV Services<br>____________________ Other

2.3	Were you or your infant offered an HIV test today?	[1] Yes<br>[0] No --> skip to 2.7

2.4	Did you know before you came to your visit today that you were going to be offered an HIV test for you or your baby?	[1] Yes<br>[0] No --> skip to 2.7
2.5	Where did you hear that you were going to be offered an HIV test? **(Do not prompt with list)**	____ ANC<br>____ SMAGs<br>____ In-facility delivery/PNC visit<br>____ Heard from other mothers that came for U5 clinic<br>_______________ Other
2.6	Did knowing that you or your infant would be offered an HIV test make you feel less likely, more likely, or just as likely to come to this appointment? **(Prompt with list of options)**	[0] Less likely --> Skip to 2.8<br>[1] More likely --> Skip to 2.8<br>[2] Just as likely --> Skip to 2.8
2.7	If you had known that you or your infant would be offered an HIV test, would you have been less likely to come, more likely to come, or just as likely to come to the visit? **(Prompt with list of options)**	[0] Less likely<br>[1] More likely<br>[2] Just as likely
2.8	Compared to other U5 visits, did this visit take a shorter amount of time, a longer amount of time, or about the same amount of time compared to other visits? **(Prompt with list of options)**	[0] Shorter<br>[1] Longer<br>[2] About the same

Interview Scripts for Exit Interviews for Mothers Done at Health Facilities.

Relevant sections translated into Tonga

SECTION 1: SURVEY SETUP AND DATA ENTRY		
Before starting the interview, fill out the information below.		
1.1	District Name:	
1.2	Health Facility Name	
1.3	Date of Interview (DD/MM/YYYY):	|___|___|/|___|___|/|___|___|___|___|
1.4	Time Start Interview (24-hr clock; HH:MM):	|___|___|:|___|___|
1.5	Time End Interview (24-hr clock; HH:MM):	|___|___|:|___|___|
1.6	Smartphone – GPS Coordinates (Decimals)	N / S |___|___|.|___|___|___|___|___|___|___|___| E/W |___|___|.|___|___|___|___|___|___|___|___|
1.7	Surveyor name:	
1.8	Surveyor ID:	|___|___|___|___|
1.9	Respondent Unique ID	|___|___|___|___|
At the time of data entry, please complete the information below.		
1.1	Data Entry Person Name:	
1.11	Data Entry Person ID:	
Read the respondent the informed consent form.		
1.12	Consent Given?	[1] Yes [0] No à *End survey*
1.13	Full Respondent Family and Given Name:	
1.14	Unique ID from randomized list	|___|___|___|___|___|___|
1.15	Contact telephone number	

SECTION 2: PERCEPTIONS OF IMMUNIZATION VISIT		
2.1	ngugwasyonzi ndoli kuyeya kuti mweebo amwana wenu ulatambula sunu?	____ Kwiya zyakulya<br>____ Vitamin A<br>____ Bukwabilizi<br>____ kutantanya bana<br>____ lugwasyo iwa sikalileka<br>____________________ Azimwi
2.2	Ndugwasyonzi yebo amwana ndotambula sunu?	____ Kwiya zyakulya<br>____ Vitamin A<br>____ Bukwabilizi<br>____ kutantanya bana<br>____ lugwasyo iwa sikalileka<br>____________________ Azimwi
2.3	ino mwa pimwa amwana wenu ali sikalileka sunu?	[1] inhya [2] pepe--> kamuya ku 2.7

2.4	ino mwakalihi nemutaninga kubola sunu kuti inwebo amwana wenu mula pimwa asikalileka	[1]inhya [2] pepe-->kamuya ku 2.7
2.5	Nkuli nkomwakamvwa kuti muyo pimwa bulwazi bwasikalileke?	____ Kuchipimo<br>____ SMAGs<br>____ kuchibadela chakutumbuka / Olo kuchipimo chacna<br>____ Ndakamvwa kulibamwi bamakaintu bakaboola kuchipimo chabana<br>_______________ Kulibamwi
2.6	sena newakahiba kuti nwebo anwana wenu muyo pimwa sikalileka mwakmvwa mbuli kutayanda kubola na mwakayandisya ku bola olo kunyina chomwakanmva?	[0] kutayanda kubola --> kamunka ku 2.8<br>[1] kuyandisya --> kamunka ku 2.8<br>[2] kunyina chomwakanmvwa --> Skip to 2.8
2.7	ino na mwakalihibide kuti nwebo amwana wenu muyo pimwa sikalileka nanga mwakanvwa mbuli kutayanda kubola na ninga mwakayandisya kubola olo na kunyina chomwakanmvwa?	[0] kutayanda kubola --> kamunka ku 2.8<br>[1] kuyandisya --> kamunka ku 2.8<br>[2] kunyina chomwakanmvwa --> Skip to 2.8
2.8	mwalangisya lweendo oyu lwa chipimo, watola chiindi chi shonto na chilanfu na chimwe biyo mbuli ayambi ma lweendo ya chipimo?	[0] chi shonto<br>[1] chilamfu<br>[2] chimwe biyo

4) Interview Script for Data Verification for Mothers Done in Local Communities

English version. These interviews will be conducted in Tonga

SECTION 1: SURVEY SETUP AND DATA ENTRY		
Before starting the interview, fill out the information below.		
1.1	District Name:	
1.2	Village / neighborhood name	
1.3	Catchment Health Facility Name	
1.4	Date of Interview (DD/MM/YYYY):	|___|___|/|___|___|/|___|___|___|___|
1.5	Time Start Interview (24-hr clock; HH:MM):	|___|___|:|___|___|
1.6	Time End Interview (24-hr clock; HH:MM):	|___|___|:|___|___|
1.7	Smartphone – GPS Coordinates (Decimals)	N / S |___|___|.|___|___|___|___|___|___|___|___| E/W |___|___|.|___|___|___|___|___|___|___|___|
1.8	Surveyor name:	
1.9	Surveyor ID:	|___|___|___|___|
1.10	Respondent Unique ID	|___|___|___|___|
At the time of data entry, please complete the information below.		
1.11	Data Entry Person Name:	
1.12	Data Entry Person ID:	
Read the respondent the informed consent form.		
1.13	Consent Given?	[1] Yes [0] No ▯ *End survey*
1.14	Full Respondent Family and Given Name:	
1.15	Unique ID from randomized list	|___|___|___|___|___|___|
1.16	Contact telephone number	

SECTION 2: Health Facility Confirmation		
2.1	Has your infant received their first round of immunizations?	[1] Yes [0] No -> End Survey
2.2	Did your infant receive their first round of immunizations at a health facility or outreach?	[1] Yes [2] Outreach--> End Survey
2.3	What was the name of that health facility?	(Choose from list of health facilities in district)
2.4	Do you remember what day you went to the health facility for the first round of immunizations?	[1] Yes [0] No --> Q2.6
2.5	What day did you go to the health facility for the first round of immunizations?	Day (Sun-Sat):________ Date: _________
2.6	Roughly how long ago did you go to the health facility for the first round of immunizations?	___ Days ago OR ___ weeks ago OR "don't remember"

SECTION 3: Data Verification
PROMPT: Can you please show me the Under-5 card for your youngest child?

PROCESS: Record the following data points from the U5 card

3.1	Picture taken?	[1] Yes [0] No -> End Survey
3.2	Name of Health Facility	______________________________
3.3	Under-5 Number	|___|___|___|___|___|___|
3.4	Date for DPT1 and OPV1 (DD:MM:YYYY)	|___|___|/|___|___|/|___|___|___|___|
3.5	PMTCT Status	[1] CE [2] MSU [3] CNE
3.6	Date of 1st PCR test (DD:MM:YYYY)<br>Fill 00/00/0000 if no test complete	|___|___|/|___|___|/|___|___|___|___|
3.7	SKIP if NO PCR Test DONE<br>What was the result of the 1st PCR Test	[1] R [2] NR [3] I

Interview Script for Data Verification for Mothers Done in Local Communities

Relevant sections translated into Tonga

**SECTION 1: SURVEY SETUP AND DATA ENTRY**		
Before starting the interviw, fill out the information below.		
1.1	District Name:	
1.2	Village / neighborhood name	
1.3	Catchment Health Facility Name	
1.4	Date of Interview (DD/MM/YYYY):	|___|___|/|___|___|/|___|___|___|___|
1.5	Time Start Interview (24-hr clock; HH:MM):	|___|___|:|___|___|
1.6	Time End Interview (24-hr clock; HH:MM):	|___|___|:|___|___|
1.7	Smartphone – GPS Coordinates (Decimals)	N / S |___|___|.|___|___|___|___|___|___|___|___|___| E/W |___|___|.|___|___|___|___|___|___|___|___|___|
1.8	Surveyor name:	
1.9	Surveyor ID:	|___|___|___|___|
1.10	Respondent Unique ID	|___|___|___|___|
At the time of data entry, please complete the information below.		
1.11	Data Entry Person Name:	
1.12	Data Entry Person ID:	
Read the respondent the informed consent form.		
1.13	Consent Given?	[1] Yes [0] No → *End survey*
1.14	Full Respondent Family and Given Name:	
1.15	Unique ID from randomized list	|___|___|___|___|___|___|
1.16	Contact telephone number	

**SECTION 2: Health Facility Confirmation**		
2.1	sena mwanawanu wakayasawa nyeleti yabakwabilizi bwakutanguna?	[1]inhya [2]pepe--> kamulekehya mpomunyawa
2.2	sena mwanawanu inyeleti yakuwabilizi yabukwabilizi bwakusanguna wakatambida kuchi bbadela na ku nkomponi kufufwafi ako mukala	
2.3	yakali nzi ihina ya chibbadela?	(kamusala amihina aya ya chibadela amu district)
2.4	sena mulayeya ibuzuba nemwaka unka ku chibbadela kuyaswa nyeleti yabukwabilizi yakusangunina?	[1] inhya [0] pepe → **Q2.6**
2.5	mbuzuba nzi nemwaka unka ku chibabdela kuyaswa nyeleti yakukwabilizi yakusangunina?	buzuba(munsodo kusika mu nsabata)
2.6	kwainda chilanfu buti mwana nakayaswa inyeleti yabukwabilizi bwakuasanguna?	kawainda mazuba ali…………. Kwainda nsondo ili……..<br>Olo seyeyede

**SECTION 3: Data Verification**<br>**PROMPT: kamundilangihya under 5 card yamwana wenu mushotso ali bana benu bonse**<br><br>**PROCESS: Record the following data points from the U5 card**		
3.1	Picture taken?	[1] Yes [0] No -> End Survey
3.2	Name of Health Facility	______________________________
3.3	Under-5 Number	|___|___|___|___|___|___|
3.4	Date for DPT1 and OPV1 (DD:MM:YYYY)	|___|___|/|___|___|/|___|___|___|___|
3.5	PMTCT Status	[1] CE [2] MSU [3] CNE
3.6	Date of 1st PCR test (DD:MM:YYYY)<br>Fill 00/00/0000 if no test complete	|___|___|/|___|___|/|___|___|___|___|
3.7	SKIP if NO PCR Test DONE<br><br>What was the result of the 1st PCR Test	[1] R [2] NR [3] I

4.6 Appendix 6: Investigator CVs

PAUL CHEE-SOONG WANG

37J Lake Road, Lusaka, Zambia
Paul.Wang@IDinsight.org
+260 (0)973 588277

Profile

Permanent resident of Zambia and applied researcher that has implemented evaluations of 12 programs in 6 countries in Africa and Asia

Experience

2011-present **IDINSIGHT** — **ZAMBIA**

Founding Partner and Principal Investigator

International development NGO that conducts rigorous field experiments to support decision-making at NGOs, foundations, governments and socially-impactful businesses

- Built executive team that has collectively overseen over 20 field experiments in Africa and Asia
- Spearheaded development of a 3-year Demand Driven Evaluations for Decisions initiative in Africa with the Clinton Health Access Initiative
- Launched health, sanitation and governance evaluations in India, Cambodia and Zambia

2009-present **TAMTAM (Together Against Malaria, Tunapenda Afya na Maisha)** — **USA & AFRICA**

Director, President

NGO that distributes free bed nets and conducts operational research in malaria prevention

- Designed and implemented 3 randomized evaluations of bed net distribution approaches in Uganda and Ghana
- Developed relationships with policymakers at national malaria control programs and USAID
- >10,000 bed nets distributed in 4 countries including partnership with Partners in Health Malawi

Summer 2008 **TECHNOSERVE** — **SWAZILAND**

Volunteer Consultant

International development NGO focused on business solutions to rural poverty

- Designed self-sustaining micro-finance model and initiated pilot with local finance organization

2006-2008 **MCKINSEY AND COMPANY** — **SOUTH AFRICA**

Business Analyst

- *Global public health:* Facilitated discussions with public health leaders to improve institutional decision making processes of global public health initiatives
- *Health care:* Created a market sizing model for an emerging health care market
- Other projects in logistics, energy and basic materials in USA and South Africa

2004-2006 **ABDUL LATIF JAMEEL POVERTY ACTION LAB (J-PAL)** — **KENYA**

Senior Evaluation Consultant, Evaluation Consultant

MIT organization that serves as a focal point for development research based on randomized trials

- Coordinated randomized evaluations of 6 health and education interventions

- J-PAL's lead representative in Kenya and oversaw an office of more than fifty staff
- Supported the formation of a new organization, Innovations for Poverty Action – Kenya (IPAK), and transitioned several J-PAL projects to IPAK

Education

2008-2011 **HARVARD KENNEDY SCHOOL** **USA**
Master in Public Administration/International Development
Carl & Lily Pforzheimer Foundation Fellow

2008-2011 **HARVARD BUSINESS SCHOOL** **USA**
Master in Business Administration
Social Enterprise Initiative Summer Fellow

2000-2004 **HARVARD COLLEGE** **USA**
Bachelor of Arts, *magna cum laude*, in Economics

Skills Proficient in STATA (statistical package)
Personal Enjoy all sports (especially soccer), camping, hiking and traveling

MINISTRY OF HEALTH HEADQUARTERS, P.O.BOX 30205,• LUSAKA, ZAMBIA
TEL +260-211-253040 MOBILE PHONE 260-977-775089 • FAX +260-211-253344 •
MBWEUPE@MOH.GOV.ZM BWEUPEM2001@YAHOO.COM

DR. MAXIMILLIAN BWEUPE

(For ease of reference Google ***max Bweupe*** *and* ***Maximillian Bweupe****)*

WORK EXPERIENCE

September 2011 – Present Ministry of Health HQ Lusaka, Zambia

Deputy Director Public Health & Research (Reproductive, Child Health & Nutrition)

And Acting Director Public Health & Research

- Providing overall Management, Leadership and Responsibility for Diseases of Public Health Concern in Zambia, including Communicable, Non-communicable, Neglected Tropical Diseases, Mental Health, Health Promotion and Nutrition. The Major communicable diseases under the Directorate are HIV, TB and Malaria while the Non-communicable Diseases are mostly diseases of lifestyle including hypertension, diabetes mellitus, obesity and cancers

February 2008-2011

Prevention of Mother to Child Transmission of HIV (PMTCT) Specialist and Head of Unit

- Responsible for the Planning, implementation, monitoring and evaluation of PMTCT in Zambia.
- Team leader and National Focal Point for policies, protocols and guidelines for the implementation of PMTCT In the whole country
- Responsible to the Permanent Secretary and Director of Public Health & Research for the effective , efficient and impact driven provision of services in Zambia, with a goal to eliminate transmission of HIV to Children by 2015
- Co-Chair of the broad-based National Technical Working Group for PMTCT and Paediatric HIV
- Chief Editor and Development Team Leader – National PMTCT Training Reference Manuals for Health Workers

February 2004- February 2008 Central Board of Health Lusaka

National Coordinator-Prevention of Mother-to-Child Transmission of HIV (PMTCT)

- Responsible for the Planning, implementation, monitoring and evaluation of PMTCT in Zambia.
- Team leader and National Focal Point for policies, protocols and guidelines for the implementation of PMTCT In the whole country
- Deputizing the Head-Reproductive Health

January 2001-February 2004 University Teaching Hospital Lusaka

Registrar in Obstetrics and Gynaecology

- Conducting of In-patient and Outpatient Care to patients attending the UTH Obstetrics and Gynaecology services
- Conducting Specialist Clinics in Obstetrics and Gynaecology, as well as follow up care
- Leading a team of Resident Medical Doctors in conducting routine

admission, on-call and operation theatre surgical treatment.

- Responsible to the Unit Consultant for smooth operations of the specialized Obstetrics and Gynaecology services

EDUCATION

2008- 2012 University of the Western Cape Republic of South Africa
Master of Public Health

2007-2008 JICA/JOICEFP Tokyo, Japan
Fellow in Reproductive Health

2000-2002 University of Zambia Lusaka, Zambia
Master of Medicine (Obstetrics & Gynaecology) Part I

1994-1996 University of Zambia Lusaka, Zambia
Bachelor of Medicine and Surgery

1989-1994 University of Zambia Lusaka, Zambia
Bachelor of Science (Human Biology)

PEER REVIEWED ARTICLES & RESEARCH CO-AUTHORED

- Infant Feeding Options, other Nonchemoprophylactic Factors and Mother-to-Child Transmission of HIV in Zambia. *Journal of The International Association of Physicians in AIDS Care* (Chicago) March 2011.
- Reducing Paediatric HIV Infection: Estimating Mother-to-Child Transmission rates in a Program Setting in Zambia. *Journal of Acquired Immune Deficiency Syndrome*. August 2010
- Antiretroviral Therapy in Antenatal Care to Increase Treatment Initiation in HIV-Infected Women: a Stepped-Wedge Evaluation. *AIDS* Jan 2010

RELATED INTERESTS AND ACTIVITIES

- UNAIDS Certified Evaluator of HIV Programs (2011)
- Member of the International AIDS Society (Past 5 years)
- Past Chair- Zambia Medical Association Public Health Board (2007-2009)
- Invited Non-Abstract Key Speaker, *International AIDS Conference*. Toronto (2006) and Mexico City (2008). Ministry of Health National Delegate to Vienna (2010).
- Member- Expert Consultation on the Integration of HIV Interventions into Maternal, Newborn and Child Health. Geneva. 2006
- Course Director – WHO Integrated Infant and Young Child feeding Training Course (2008)

MINISTRY OF HEALTH, HEADQUARTERS • BOX 30205 • LUSAKA •
PHONE 260-977-879101 •E-MAIL pascychanda@yahoo.com

PASCALINA CHANDA-KAPATA, BSC, MPH, Ph.D

PERSONAL DETAILS

D.O.B	25th June 1977
NRC#	304056/61/1
Marital status	Married
Languages	English, Bemba

ACHIEVEMENTS

I have successfully managed the human and financial resources which have been affiliated with the various programmes I have worked on over my professional career. I have managed to contribute to health systems strengthening, human and infrastructure capacity building and evidence based decision making.

I have spearheaded the development and institutionalisation of the health research system in Zambia; provided oversight for the development of the first ever legal framework for conducting health research involving live human participants; have successfully hosted the national health research conferences; managed the inception and administration of the National Health Research Ethics and Advisory Committees and developed a system for coordination of all health research being conducted in the country.

I have served as a board member for the National Science and Technology Council.

I successfully developed the malaria research programme at the National Malaria Control Centre and expanded collaborations and grant portifolio; Mentored students in research and scientific publication of research findings, capacity development for district personnel in research skills and data management; effectively managed the anti-malarial drug resistance and compliance monitoring system; maintained and expanded the network for malaria researchers leading to increased malaria research outputs in the country.

I have published close to 30 articles of special papers in peer reviewed journals of which I have been first author in 13 articles. I have made several scientific presentations at international and local meetings, symposiums and conferences.

I have also served as invited reviewer for public health related journals.I am involved with various networks of partnerships and are collaborating with other professionals working in various aspects of global health and health system strengthening.

EDUCATION

2008 – 2011	University of Camerino, Italy.<br>*Doctorate in Environmental Sciences and Public Health: Programme on Malaria amd Human Development*
2005 - 2006	University of Cape Town, South Africa.<br>*Masters of Public Health in Health Economics*
2003	Boston University School of Public Health, USA.<br>*Post Graduate Certificate in International Health*

1996 – 2001 University of Zambia, Zambia
Bachelor of Science in Parasitology and Entomology

1990 – 1994 Fatima Girls' Secondary School, Ndola, Zambia.
GCSE (Secondary School Certificate)

PROFESSIONAL EXPERIENCE

Nov 2008 to Date Ministry of Health Headquarters Lusaka

Principal Surveillance and Research Officer

Strategic planning and coordinating health research and surveillance systems.

Resource mobilisation and grant writing

Coordinating research or scientific meetings

Providing technical advise on information requirements on matters related to communicable and non-communicable diseases

Conducting research and surveillance on priority public health diseases and conditions.

Reviewing proposals for health research to ensure conformity with national standards and priorities

Providing technical support to stakeholders in health research including bioethics.

Coordinating the National Health Research Ethics and Advisory activities.

Developing a legal framework for conducting health research involving live human participants

Developing guidelines for regulation of exportation and importation of biological materials for research purposes

Coordinating the dissemination of health research information

Participating in local and international health conferences

Compiling ministerial and department briefs; quarterly reports and annual reports.

Planning and managing health research funds and personnel.

Supervisions local and international students attached to the research unit.

Jan 2012 to date Cavendish University Zambia

Part-time Lecturer for MPH students, Module Leader for Fundamentals of Global Health and Infectious Diseases Courses.

- Developed the modules for Global Health and Infectious Diseases courses for MPH students.
- Teaching and continous assessment in the two courses for both full time and part-time students.
- Grant writing

April 2011 to date Page Press, Italy

Editorial Board Member for Malaria Reports Journal

Peer review of scientific journals for publication

June 2007 to June 2010 National Science and Technology Council Zambia

Board Member

To advise government on science and technology activities in the country

To administer the Science and Technology Development Fund

Monitoring research activities

2004 to Nov 2008 National Malaria Control Centre Lusaka

Operational Research Officer

Strategic planning and costing

Economic evaluations

Preparing, quarterly and annual progress reports

Planning and conducting malaria operational research in the country

Coordinating and documenting partner malaria research activities

Conducting antimalarial drug efficacy, compliance and safety studies

Developing performance monitoring indicators for malaria control

Monitoring and evaluation

Insecticide resistance monitoring

Planning and conducting specialized surveys

Reviewing research proposals for funding

Grant applications, costing and programming

Supervising local and international students researching on malaria; managing a capacity building malaria grant for MPH students

Providing technical support to districts on malaria research

Dissemination of research findings at local and international forums.

2003 to 2004 National Malaria Control Centre Lusaka

Scientific and Communications Officer

Organising regional meetings for Southern Africa Malaria Managers and the Malaria in Pregnancy for East and Southern Africa.

Adapting the standard WHO protocol for drug efficacy monitoring to suit the Zambia situation for monitoring of Artemisinin-based combination therapies.

Worked on various applied research projects on malaria.

Appointed member of the antimicrobial resistance monitoring working group.

2002 – 2003 University of Zambia Lusaka

Tutor

Conducted laboratory sessions and tutorials for first and second year biological science students.

Provided progress reports through continuous assessment tests.

2001–2002 National Malaria Control Centre Lusaka

Research Assistant

Developing of proposals for malaria vector susceptibility projects

Collection of historical data for mapping of Malaria Risk in Africa (MARA) project.

Geo located sites where malaria research has been conducted in Zambia using maps and Geo Data CDs.

Compiling data on health facilities in Zambia.

Conducted a field trial on the efficacy of Larvex® 100 for larvae control.

Training rural health workers in a malariology course in conjuction with Christian Children Fund (CCF).

CONSULTANCY EXPERIENCE

November –December 2011–Technical Consultant -Capacity Building in Research Ethics, Guidelines and Standards of Operation, National HIV/AIDS/STI/TB Council, Lusaka, Zambia.

Jan 2010 to date Technical Consultant – Zambia Chlorohexidine Trial, Centre for Global Health and Development, Boston University School of Public Health.

Oct 2008 to April 2009 Lead Consultant – Impact Evaluation of the Global Funds for Malaria, Churches Health Association of Zambia, Lusaka.

Dec 2008 – Jan 2009 Health Economics Consultant - Diagnostic costing study, TDRC WHO grant.

May 2007 CEGAA Associate – UNAIDS Expenditure Tracking Assessment.

2006 - 2007 Health Economist Consultant – HEPNET, User Fees Impact Study.

February 2007 Resource Consultant – General Nursing Council, Zambia.

September 2005 Research Consultant – HLSP Institute, UK.

2003 Research Consultant–NetMark Research Department, Academy for Education Development.

HONORS/AWARDS RECEIVED

2012: Grand Challenges Canada Rising Star in Global Health, Grant # 0135-01

2011: Invited Speaker, Montreal Global Health Meeting held in November 2011,Canada.

2008-2010 WHO/TDR Implementation Research Grant, Grant #A70170

June 2007 to June 2010 **Board Member of The Council** for National Science and Technology in Zambia. Additionally member of the Science and Technology Development Fund Committee.

2005 Swedish International Development Agency (SIDA) - University of Cape Town , Health Economics Unit, MPH (Health Economics) **Scholarship**, RSA.

2003 Centre for International Health and Development, Boston University School of Public Health **Training Award**. For post graduate certificate in international health.

1996 Government of Republic of Zambia Bursaries Committee **Training Grant**, Lusaka, Zambia

PUBLICATIONS

1. Chanda E, Coleman M, Kleinschmidt I, Hemingway J, Hamainza B, Masaninga F, Chanda-Kapata P, Baboo KS, Dürrheim DN, Coleman M. Impact assessment of malaria vector control using routine surveillance data in Zambia: implications for monitoring and evaluation. Malar J. 2012 Dec 29;11(1):437.
2. Kapata N, **Chanda-Kapata P,** O'Grady J, Bates M, Mwaba P, Janssen S, Marais B, Cobelens F, Grobusch M, Zumla A.Trends in Childhood Tuberculosis in Zambia: A Situation Analysis. J Trop Pediatr. 2012 Dec 12.
3. Freddie Masaninga*, Emmanuel Chanda, **Pascalina Chanda-Kapata**, Busiku Hamainza, Hieronymo T Masendu,Mulakwa Kamuliwo, Wambinji Kapelwa, John Chimumbwa, John Govere, Ibrahima Soce Fall, Olusegun Babaniyi1 Review of the malaria epidemiology and trends in Zambia. *Asian Pacific Journal of Tropical Biomedicine* (2012)1-5.
4. Kapata N, **Chanda-Kapata P**, Grobusch MP, O'Grady J, Bates M, Mwaba P, Zumla A.Leprosy trends in Zambia 1991-2009**.** Trop Med Int Health. 2012 Jul 29. doi: 10.1111/j.1365-3156.2012.03050.x.

5. **Chanda-Kapata** P, Campbell S, Zarowsky C.Developing a national health research system: participatory approaches to legislative, institutional and networking dimensions in Zambia. *Health Res Policy Syst.* 2012 Jun 6;10(1):17

6. Kapata N, **Chanda-Kapata P**, Grobusch MP, O'Grady J, Schwank S, Bates M, Jansenn S, Mwinga A, Cobelens F, Mwaba P, Zumla A. Scale-up of TB and HIV programme collaborative activities in Zambia - a 10-year review. Trop Med Int Health. 2012 Apr 5. doi: 10.1111/j.1365-3156.2012.02981.x

7. Kapata N, **Chanda-Kapata P**, O'Grady J, Schwank S, Bates M, Mukonka V, Zumla A, Mwaba P. Trends of Zambia's tuberculosis burden over the past two decades. *Trop Med Intl Health.* 2011 Jul 29. doi: 10.1111/j.1365-3156.2011.02849.x

8. **Chanda P**, Hamainza B, Moonga HB, Chalwe V, Pagnoni F. Community case management of malaria using ACT and RDT in two districts in Zambia: achieving high adherence to test results using community health workers. Malar J. 2011 Jun 9;10:158.

9. **Chanda P**, Hamainza B, Moonga HB, Chalwe V, Banda P, Pagnoni F.Relative costs and effectiveness of treating uncomplicated malaria in two rural districts in Zambia: implications for nationwide scale-up of home-based management. Malar J. 2011 Jun 9;10:159.

10. **Chanda P**. The quality of malaria case management and the perceived value of malaria risk reduction in four districts in Zambia. Ph.D Thesis. University of Camerino, June 2011.

11. Barnes KI, **Chanda P**, Ab Barnabas G. Impact of the large-scale deployment of artemether/lumefantrine on the malaria disease burden in Africa: case studies of South Africa, Zambia and Ethiopia.Malaria Journal 2009;8 Supll 1:S8.

12. Pearce RJ, Pota H, Evehe MS, Bâ el-H, Mombo-Ngoma G, Malisa AL, Ord R, Inojosa W, Matondo A, Diallo DA, Mbacham W, van den Broek IV, Swarthout TD, Getachew A, Dejene S, Grobusch MP, Njie F, Dunyo S, Kweku M, Owusu-Agyei S, Chandramohan D, Bonnet M, Guthmann JP, Clarke S, Barnes KI, Streat E, Katokele ST, Uusiku P, Agboghoroma CO, Elegba OY, Cissé B, A-Elbasit IE, Giha HA, Kachur SP, Lynch C, Rwakimari JB, **Chanda P**, Hawela M, Sharp B, Naidoo I, Roper C. Multiple origins and regional dispersal of resistant dhps in African Plasmodium falciparum malaria. PLoS Med. 2009 Apr 14;6(4):e1000055. Epub 2009 Apr 14.

13. **Chanda P**, Castillo-Riquelme M, Masiye F. Cost-effectiveness analysis of the available strategies for diagnosing malaria in outpatient clinics in Zambia. Cost Eff Resour Alloc. 2009 Apr 8;7:5.

14. **Chanda P**, Hamainza B, Mulenga S, Chalwe V, Msiska C, Chizema-Kawesha E. Early results of integrated malaria control and implications for the management of fever in under-five children at a peripheral health facility: a case study of Chongwe rural health centre in Zambia. Malar J. 2009 Mar 17;8:49.

15. Masiye F, Chitah BM, **Chanda P** and Simeo F. 2008. Removal of user fees at Primary Health Care facilities in Zambia: A study of effects on utilisation and quality of care. EQUINET Discussion Paper series 57. EQUINET, UCT HEU: Harare.

16. Sipililanyambe, N., Simon JL, **Chanda P**, Olumese P, Snow RW, Hamer DH. 2008. From chloroquine to artemether-lumefantrine: the process of drug policy change in Zambia. Malar J 7:25.

17. Davidson H. Hamer, Micky Ndhlovu, Dejan Zurovac, MD, Matthew Fox, Kojo Yeboah-Antwi, **Pascalina Chanda**, Naawa Sipilinyambe, Jonathon L. Simon, Robert W. Snow. 2007. Improved Diagnostic Testing and Malaria Treatment Practices in Zambia. JAMA 297:2227-2231.

18. Zurovac D, Ndhlovu M, Sipilanyambe N, **Chanda P**, Hamer DH, Simon JL, Snow RW. 2007. Paediatric malaria case management with artemether-lumefantrine in Zambia: a repeat cross sectional study. Malar J 6(1):31.

19. **Chanda P**, Felix Masiye, Bona M Chitah, Naawa Sipilanyambe, Moonga Hawela, Patrick Banda, Tuoyo Okorosobo. A cost-effectiveness analysis of artemether lumefantrine for treatment of uncomplicated malaria in Zambia. Malaria Journal 2007, 6:21. doi:10.1186/1475-2875-6-21.

20. **Chanda P,** Chanda E, MB Hawela MB, Chizema –Kawesha E. Baseline parasitological assessments and accuracy of rapid diagnostic tests in districts implementing indoor residual spraying for malaria prevention in Zambia. Medical Journal of Zambia 2007 ,34:2

21. Chanda E, **Chanda P**, Namafente O, Kandyata A, Chizema –Kawesha E. Laboratory and Simulation Field Trials Comparative Efficacy of Balicillus thuringensis var. israelensis and Abate ® against Anopheles gambiae s. l larvae (Diptera Culicidae). Medical Journal of Zambia 2007 ,34:2
22. **Chanda P**, Hamainza B, Hawela MB, Mharakurwa S, Shinondo C, Rope C, Pota H

    The frequency of Plasmodium falciparum dihydrofolate reductase and dihhydropteroate synthase resistance markers in six districts in Zambia. Medical Journal of Zambia 2007 ,34:2
23. Hamainza B, **Chanda P**, Hawela M, Chizema-Kawesha E. Assessment of Patient Compliance to the use of Artemether – Lumefantrine for the treatment of uncomplicated Malaria in Zambia Medical Journal of Zambia 2007 ,34:2; 62-70
24. **Chanda P**, HB Moonga, E Chanda, Elizabeth Chizema – Kawesha.The Capacity of Community Health Workers and Caretakers in rendering Home Management of Malaria in Zambia. Medical Journal of Zambia 2007 ,34:2
25. Hamainza B, **Chanda P**, Hawela M, Chizema-Kawesha E Therapeutic efficacy of Artemether-lumefantrine and Sulphadoxine –pyrimethamine for the treatment of uncomplicated plasmodium falciparum Malaria in Zambian Children. Medical Journal of Zambia 2007 ,34:2; 81-
26. **Chanda P**, Chipeta J, Chimutete M, Kango M , Ndhlovu M, Msiska C, Kabalo A , Kennedy AC, Wamulume P. An Assessment of management of severe malaria in Zambia health facilities. Medical Journal of Zambia 2007 ,34:2; 92-97
27. **Chanda P**. 2006. Cost and Cost-effectiveness Analysis of the Available Strategies for Diagnosing Malaria in Outpatient Clinics in Zambia. Dissertation as partial fulfilment for Masters in Health Economics. University of Cape Town.
28. **Chanda P**, Hawela M, Sipilanyambe, N, Kango M. 2006. Assesment of the therapeutic efficacy of a paediatric formulation of artemether-lumefantrine (Coartesiane®) for the treatment of uncomplicated Plasmodium falciparum malaria in children in Zambia. Malar J 5(1):75.
29. Mudondo C, **Chanda P**, Ndhlovu M, Wamulume P. 2005. Artemisini-based combination therapy: From policy change to implementation. Country Report. Accessible at http://www.who.int
30. **Chanda P**, Sikaala C, Kapelwa W, Nkunika S, MacDonald M, Thea DM, MacLeod WB, Sipilanyambe N, Hamer DH.2004. Decreasing efficacy of sulphadoxine-pyrimethamine (SP) in Zambian children. In Proceedings of the 53rd Annual Meeting of the American Society of Tropical Medicine and Hygiene, 7th –11th November 2004, Miami, FL. Abstract 708.
31. **Chanda P**, Sikaala CH, Kapelwa W, Moonga H, Njunju E, Macdonald M, Thea D, Hamer DH, Sipilanyambe N.2004. Assesment of the therapeutic efficacy of artemether-lumefantrine (Coartem®) and sulphadoxine-pyrimethamine (SP)-artesunate in Zambian children. In Proceedings of the 53rd Annual Meeting of the Society of Tropical Medicine and Hygiene. Miami, FL. Abstract 213.
32. **Chanda P**, Hazemba O, d'Allesandro U, Sipilanyambe N.2004. Compliance with artemether-lumefantirne (Coartem®) for the treatment of uncomplicated Plasmodium falciparum malaria in Zambia. In Proceedings of the American Society of Tropical Medicine and Hygiene 53rd Annual Meeting, 7th –11th November 2004, Miami, FL. Abstract 943
33. **Chanda P**. 2004. A community based assessment of the safety of Coartem® in five districts in Zambia. NMCC/CBoH Report. Lusaka. Zambia.
34. National Malaria Control Centre. 2004. District Planning Guidelines for Malaria Control. NMCC/CBoH Document. Lusaka. Zambia.

REFERENCES

Dr Felix Masiye, PhD
Department of Economics
University of Zambia
LUSAKA
fmasiye@yahoo.com

Dr Peter Mwaba

Permanent Secretary
Ministry of Health
LUSAKA
pbmwaba2000@yahoo.com

Mr. Busiku Hamainza
Principal Operations Research Officer
National Malaria Control Centre
LUSAKA
bossbusk@gmail.com

PROFESSIONAL AFFILIATIONS

- Member of the Health care financing technical working group
- Member of the National Health Research Ethics Committee
- Member of the National Health Research Advisory Committee
- Technical Subcommittee member for research in traditional medicines.
- Member of the Technical Working Group on Gender Mainstreaming in the Health Sector
- Invited reviewer for various health related scientific journals
- International Bureau of Epilepsy (IBE) member of the Research Taskforce.
- Member of the SADC Research Constituency

RESEARCH FUNDING HISTORY

2012-2014	National TB Prevalence Survey, MOH/USAID/CDC, USD4.6 Million
Oct 2012-Oct 2013	Grand Challenges Canada, Rising Star in Global Health Grant #0135-01, CAD$113,000.
Jan 2012- February 2013	World Bank, Malaria Booster Project, USD106444.
2010	IDRC/Health Research Capacity Strengthening Learning Grant, USD5,000.
2009	MACEPA/PATH Capacity building funding for Dissertation Research grant, USD10,000
2008-2010	WHO/UNDP/WORLD BANK/TDR Implementation Research Grant # A70170, USD75,000.

Mwango Albert, Dr. BSc HB, MBChB, MPH **April 18th 2013**

CURRICULUM VITAE

DATE OF BIRTH: 25th March 1974
NATIONALITY: Zambian
NATIONAL REGISTRATION No.: 318530/67/1
PASSPORT No.: ZP000189
E-MAIL: mwangoaj@yahoo.co.uk; albert.mwango@moh.gov.zm
Cell (Zambia): +260-950-230-522

QUALIFICATIONS

MASTERS OF PUBLIC HEALTH (MPH), VANDERBILT UNIVERSITY SCHOOL OF MEDICINE, NASHVILLE TN, USA

Period: September 2010 to May 11 2012.

BACHELOR OF MEDICINE AND BACHELOR OF SURGERY (MBChB), SCHOOL OF MEDICINE UNIVERSITY OF ZAMBIA

BACHELOR OF HUMAN BIOLOGY (BSc HB), SCHOOL OF MEDICINE UNIVERSITY OF ZAMBIA

Period: February 1995 to January 2002.

EDUCATION

IGCSE 'O' Level Certificate, 1992, Simba International School Ndola, Zambia

Subjects: Mathematics, French, English, Business Studies, Biology, Physics, Chemistry, and Computer Studies.

CAREER PLAN

I am employed as the National Antiretroviral Programme Coordinator and HIV/STI specialist for Zambia at the Ministry of Health. I intend to follow this up with a PhD in Global Health with a focus on Health Informatics and HIV medicine.

WORK EXPERIENCE

CURRENT JOB EXPERIENCE

Ministry of Health (MOH) and Central Board of Heath (CBOH), Zambia
Title: *National Antiretroviral Programme Coordinator*

Responsible to: Director Clinical Care & Diagnostic Services
Duties: Planning and coordinating the implementation of the ARV programme in the country; assisting in formulating and interpreting policy guidelines, developing strategies for strengthening systems in the administration of antiretroviral drugs; monitoring and evaluating antiretroviral therapy programme implementation; facilitating training of health personnel.

Period: May 24th 2004 – 23rd May 2006 (CBOH); 24th May 2006 to date (MOH)

PREVIOUS JOB EXPERIENCE

1. Ndola Central Hospital
Title: *Senior Resident Medical Officer and ART Centre Coordinator*

Responsible to: Director Clinical Services
Duties: Manning Emergency (Casualty) Room and OPD activities; Antiretroviral Centre Committee member and; Antiretroviral Therapy clinic coordinator and medical officer in the introductory public sector pilot programme for Antiretroviral Therapy at Ndola Central Hospital. During which 850 patients were commenced and monitored on ART.

Number of Subordinates: ten (10) Clinical officers
Period: April 28, 2003 – May 23, 2004

2. Ndola Central Hospital
Title: *Junior Resident Medical Officer*

Responsible to: Director Clinical Services/Heads of Department
Duties: Rotations in the Departments of Medicine (3 months), Paediatrics (3 months), Obstetrics and Gynaecology (7 months)

Number of Subordinates: six (06)
Period: February 2002- to April 28, 2003

WORKSHOPS AND COURSES

Regional consultative meetings on development of strategies for implementing antiretroviral therapy programmes in resource poor settings; strategic meetings for developing proposals for Global Fund, World Bank (ZANARA), USG PEPFAR, Italian Fund/WHO, UNITAID/UNICEF, UNITAID/Clinton Foundation; trainings for clinicians, pharmacovigilance, programme monitoring and evaluation and nutrition interventions in HIV/AIDS. Providing expert consultative input to WHO Headquarters strategies; UAB Summer Institute of Public Health; UAB Scientific Papers Writing course;

CONSULTANT SERVICES

1. “Zambia Contextualization of HIV and AIDS Treatment Literacy Toolkit for Communities”; Employer Name: Southern Africa HIV/AIDS Information Dissemination Service, April 1, 2008 – May 10, 2008

2. “Review and provision of expert advice on content for print, radio and television materials on HIV and AIDS in Children and Alcohol Abuse”; Employer Name: Zambia Centre for Communication Programmes; August 16th – December 2008

3. “Adaptation of standard operating Procedures for Antiretroviral therapy and Adherence counselling for use in public institutions in Zambia”; Employer Name: Family Health International; September 16-27th 2007

4. “Review of Antiretroviral therapy Available in Zambia 2007”; Employer Name: Refugee Legal Centre, United Kingdom; September 17-18th 2007

5. “Country Assessment of Zambia Diflucan Partnership Programme”; Employer Name: Johns Hopkins Programme for Information and education for Gynaecology and Obstetrics Corporation; September 14 2006– December 31st, 2006

6. “Zambia Contextualization of HIV and AIDS Treatment Literacy Toolkit for Women”; Employer Name: Southern Africa HIV/AIDS Information Dissemination Service, November 20, 2006 – November 30, 2006

7. “Evaluation of the Zambia National Antiretroviral Therapy Implementation process (2004-2005) and development of a three year implementation plan (2006-2008)”; Employer Name: Abt Associates; January 30, 2006 - April 30, 2006.

8. “Policy and Practice Harmonisation review of Kwatu Radio Drama” ; Employer Name: Zambia Centre for Communication Programmes; June 23, 2005 – July 8, 2005.

ONGOING STUDIES OR PROJECTS

1. Lamivudine (3TC) and Emtricitabine (FTC) in ARV Treatment Regimens in Resource-Limited Settings observational study; Sponsors Ministry of Health Zambia, United States Centers for Disease Control and Prevention (CDC)
2. Scaling Up TB Prevention, Screening, diagnosis and Care in Zambia, Implementing the WHO 3 I's in Zambia; Sponsors United States Centers for Disease Control and Prevention (CDC)
3. Evaluation of an Integrated Community-Based and Clinical HIV/AIDS Program in Sinazongwe District, Zambia; Sponsor: United States Centers for Disease Control and Prevention (CDC)

ABSTRACTS (* Oral Presentation)

1. Eric Fleutelot and **Albert Mwango**. *IAS TasP regional consultation report*. 3rd International HIV Workshop on Treatment as Prevention. Vancouver, BC, Canada. April 22-25, 2013. Late Breaker "ACCEPTED".

2. Izukanji Sikazwe, **Albert Mwango**, Kenichi Komada, Shinsuke Miyano, Callie Scott, Gardner Syakantu. *A cost analysis of the expansion of antiretroviral treatment to the rural health centre level through mobile HIV services in Zambia*. 7th IAS Conference on HIV Pathogenesis, Treatment and Prevention 30 June -3 July 2013, Kuala Lumpur, Malaysia. "ACCEPTED".

3. **Albert Mwango**, Izukanji Sikazwe, Kenichi Komada, Shinsuke Miyano, Gardner Syakantu. *Outcomes of national expansion program for antiretroviral treatment to rural health centre level through mobile HIV services in Zambia*. 7th IAS Conference on HIV Pathogenesis, Treatment and Prevention 30 June -3 July 2013, Kuala Lumpur, Malaysia. "ACCEPTED".

4. Lloyd Mulenga, **Albert Mwango**, Patrick Musonda, Mary-Ann Davies, Aggrey Mweemba, Alexandra Calmy, Jeffrey Stringer, Olivia Keiser, Benjamin Chi, Gilles Wandeler. Renal Function and Outcomes of Tenofovir-Containing ART in Zambia. *20thConference on Retroviruses and Opportunistic Infections, Atlanta, USA*. March 3-6, 2013; Paper 816 (M-142).

5. Michelle Li, Brad Guffey, Patrick Musonda, Izukanji Sikazwe, **Albert Mwango**, Benjamin Chi, Jeffrey Stringer. ART initiation substantially improves retention among patients enrolling in care in Lusaka, Zambia. *20thConference on Retroviruses and Opportunistic Infections, Atlanta, USA*. March 3-6, 2013; Paper 93.

6. Albert Mwango, Benjamin H Chi, Douglas C Heimburger, Sten H Vermund. Program retention efficiency in the antiretroviral therapy program in Zambia: A retrospective cohort study. *Sothern African HIV clinicians Society Conference 2012.*

7. C. Bositis, D. Patel, S. Lakhi, **A. Mwango**, K. Bowa, M. Hossain, S. Schneider, R. Sheneberger. Scaling up local human resource capacity: experience of the Zambian HIV residency program. : IAS 2010 Abstract no. WEPE0862 "

8. B. Chi, A. Westfall, M. Fox, S. Phiri, H. Prozesky, L. Fairall, **A. Mwango**, A. Boulle, M. Egger, J. Stringer, M. Brinkhof, O. Keiser. Empirically defining lost to follow-up for antiretroviral therapy programs in Southern Africa. : IAS 2010: Abstract no. THPE0422 "

9. * **Mwango A**, Giganti M, Mulenga L, Reid S, Chisembele-Taylor A, Chintu N, Chi B, Stringer E, Stringer J. First-line tenofovir ART in Zambia. *16thConference on Retroviruses and Opportunistic Infections, Montreal, Canada*. February 8-11, 2009; Abstract 142.

10. Stringer J, Mulenga L, Giganti M, Reid S, Chisembele-Taylor A, Chintu N, Chi B, Stringer E, **Mwango A**. Effectiveness of generic vs. proprietary first-line ARV regimens in a primary health care setting: Lusaka, Zambia. *16th Conference on Retroviruses and Opportunistic Infections, Montreal, Canada*. February 8-11, 2009; Abstract 611.

11. L. Luwatula, R. Hughes, E. Sinyinza, **A. Mwango**. Strengthening pre-service education in Zambia for the provision of HIV/AIDS services and care. : *AIDS 2008 - XVII International AIDS Conference*: Abstract no. MOPE0848"

12. Sinkala M, Levy J, Zulu I, **Mwango A**, Stringer E, Chi B, Reid S, Ellerbrock T, Bulterys M, Stringer J. Rapid scale-up of antiretroviral services in Zambia: 1-year clinical and immunological outcomes. *13th Conference on Retroviruses and Opportunistic Infections in Denver, CO*,USA. February 5-8, 2006; Abstract 64.

13. L. Luwatula, R. Hughes, E. Sinyinza, **A. Mwango**. Addressing human resources: results of a crash program to train students in Zambia before graduation. : *AIDS 2006 - XVI International AIDS Conference*: Abstract no. CDD1280

PUBLICATIONS (Co-Author)

1. Marseille E, Giganti MJ, **Mwango A**, Chisembele-Taylor A, Mulenga L, Over M, Kahn JG, Stringer JS. *Taking ART to scale: determinants of the cost and cost-effectiveness of antiretroviral therapy in 45 clinical sites in Zambia*. Source Health Strategies International, Oakland, CA, USA. emarseille@comcast.net

2. Giganti MJ, Limbada M, **Mwango A**, Moyo C, Mulenga LB, Guffey MB, Mulenga PL, Bolton-Moore C, Stringer JS, Chi BH. *Six-month hemoglobin concentration and its association with subsequent mortality among adults on antiretroviral therapy in Lusaka, Zambia*. J Acquir Immune Defic Syndr. 2012 Sep 1;61(1):120-3. PubMed PMID: 22659648.

3. Koethe JR, Blevins M, Bosire C, Nyirenda C, Kabagambe EK, **Mwango A**, Kasongo W, Zulu I, Shepherd BE, Heimburger DC. *Self-reported dietary intake and appetite predict early treatment outcome among low-BMI adults initiating HIV treatment in sub-Saharan Africa.* Public Health Nutr. 2012 Jun 13:1-10. [Epub ahead of print] PubMed PMID: 22691872.

4. Stringer JS, **Mwango A**, Giganti MJ, Mulenga L, Levy JW, Stringer EM, Mulenga P, Saag MS, Musonda P, Williams FB, Reid SE, Chi BH. *Effectiveness of generic and proprietary first-line anti-retroviral regimens in a primary health care setting in Lusaka, Zambia: a cohort study*. Int J Epidemiol. 2012 Apr;41(2):448-59. PubMed PMID: 22493326; PubMed Central PMCID: PMC3324461.

5. Chi BH, **Mwango A**, Giganti MJ, Sikazwe I, Moyo C, Schuttner L, Mulenga LB, Bolton-Moore C, Chintu NT, Sheneberger R, Stringer EM, Stringer JS. *Comparative outcomes of tenofovir-based and zidovudine-based antiretroviral therapy regimens in Lusaka, Zambia.* J Acquir Immune Defic Syndr. 2011 Dec 15;58(5):475-81. PubMed PMID: 21857354; PubMed Central PMCID: PMC3215810.

6. Chi BH, Yiannoutsos CT, Westfall AO, Newman JE, Zhou J, Cesar C, Brinkhof MW, **Mwango A**, Balestre E, Carriquiry G, Sirisanthana T, Mukumbi H, Martin JN, Grimsrud A, Bacon M, Thiebaut R; International Epidemiologic Databases to Evaluate AIDS Collaboration. *Universal definition of loss to follow-up in HIV treatment programs: a statistical analysis of 111 facilities in Africa, Asia, and Latin America.* PLoS Med. 2011 Oct;8(10):e1001111. Epub 2011 Oct 25. PubMed PMID: 22039357; PubMed Central PMCID: PMC3201937.

7. Koethe JR, Blevins M, Nyirenda C, Kabagambe EK, Shepherd BE, Wester CW, Zulu I, Chiasera JM, Mulenga LB, **Mwango A**, Heimburger DC. *Nutrition and inflammation serum biomarkers are associated with 12-week mortality among malnourished adults initiating antiretroviral therapy in Zambia.* J Int AIDS Soc. 2011 Apr 10;14:19. PubMed PMID: 21477359; PubMed Central PMCID: PMC3094357.

8. Schöni-Affolter F, Keiser O, **Mwango A**, Stringer J, Ledergerber B, Mulenga L, Bucher HC, Westfall AO, Calmy A, Boulle A, Chintu N, Egger M, Chi BH; Swiss HIV Cohort Study; IeDEA Southern Africa. *Estimating loss to follow-up in HIV-infected patients on antiretroviral therapy: the effect of the competing risk of death in Zambia and Switzerland.* PLoS One. 2011;6(12):e27919. Epub 2011 Dec 19. PubMed PMID: 22205933; PubMed Central PMCID: PMC3242760.

9. Chi BH, **Mwango A**, Giganti M, Mulenga LB, Tambatamba-Chapula B, Reid SE, Bolton-Moore C, Chintu N, Mulenga PL, Stringer EM, Sheneberger R, Mwaba P, Stringer JS. Early clinical and programmatic outcomes with tenofovir-based antiretroviral therapy in Zambia. J Acquir Immune Defic Syndr. 2010 May 1;54(1):63-70. PubMed PMID: 20009765; PubMed Central PMCID: PMC2862003.

10. Chi BH, Cantrell RA, **Mwango A**, Westfall AO, Mutale W, Limbada M, Mulenga LB, Vermund SH, Stringer JS. *An empirical approach to defining loss to follow-up among patients enrolled in antiretroviral treatment programs.* Am J Epidemiol. 2010 Apr 15;171(8):924-31. Epub 2010 Mar 10. PubMed PMID: 20219765; PubMed Central PMCID: PMC2850972.

11. Chi BH, Cantrell RA, Zulu I, Mulenga LB, Levy JW, Tambatamba BC, Reid S, **Mwango A**, Mwinga A, Bulterys M, Saag MS, Stringer JS. *Adherence to first-line antiretroviral therapy affects non-virologic outcomes among patients on treatment for more than 12 months in Lusaka, Zambia.* Int J Epidemiol. 2009 Jun;38(3):746-56. Epub 2009 Feb 17. PubMed PMID: 19223334; PubMed Central PMCID: PMC2689395.

12. Morris MB, Chapula BT, Chi BH, **Mwango A**, Chi HF, Mwanza J, Manda H, Bolton C, Pankratz DS, Stringer JS, Reid SE. *Use of task-shifting to rapidly scale-up HIV treatment services: experiences from Lusaka, Zambia.* BMC Health Serv Res. 2009 Jan 9;9:5. PubMed PMID: 19134202; PubMed Central PMCID: PMC2628658.

13. Torpey KE, Kabaso ME, Mutale LN, Kamanga MK, **Mwango A**, Simpungwe J, Suzuki C, Mukadi YD. *Adherence support workers: a way to address human resource constraints in antiretroviral treatment programs in the public health setting in Zambia.* PLoS One. 2008 May 21;3(5):e2204. PubMed PMID: 18493615; PubMed Central PMCID: PMC2377331.

14. Bolton-Moore C, Mubiana-Mbewe M, Cantrell RA, Chintu N, Stringer EM, Chi BH, Sinkala M, Kankasa C, Wilson CM, Wilfert CM, **Mwango A**, Levy J, Abrams EJ, Bulterys M, Stringer JS. *Clinical outcomes and CD4 cell response in children receiving antiretroviral therapy at primary health care facilities in Zambia.* JAMA. 2007 Oct 24;298(16):1888-99. PubMed PMID: 17954540.

15. Huff-Rousselle M, Simooya O, Kabwe V, Hollander I, Handema R, **Mwango A**, Mwape E. *Pharmacovigilance and new essential drugs in Africa: Zambia draws lessons from its own experiences and beyond. Glob Public Health.* 2007;2(2):184-203. PubMed PMID: 19280399.

16. Stringer JS, Zulu I, Levy J, Stringer EM, **Mwango A**, Chi BH, Mtonga V, Reid S, Cantrell RA, Bulterys M, Saag MS, Marlink RG, Mwinga A, Ellerbrock TV, Sinkala M. *Rapid scale-up of antiretroviral therapy at primary care sites in Zambia: feasibility and early outcomes.* JAMA. 2006 Aug 16;296(7):782-93. PubMed PMID: 16905784.

CURRICULUM VITAE- DR PENELOPE KALESHA

Full name: Dr. Penelope Kalesha-Masumbu

Title: Child Health Specialist

Nationality: Zambian

Sex: Female

Date of Birth: 01st June, 1970

Residential address: Plot 19443, Shantumbu Road, Rockfield, Lusaka, Zambia

Marital Status: Married

Postal address: P.O. Box F.W 191, Lusaka

Cell: +260-95-574-0455

Emergency contact: Dr Aubrey Masumbu, +260-96-674-0455

E-Mail Address: pennykalesha@yahoo.co.uk : pennykalesha@gmail.com

Highest Academic Qualifications

Post Graduate Certificates

Child Survival Reaching the Target, Uppsala University, Sweden, March 2008-January 2009,

Post Graduate 2006 Masters in International Health, University of Copenhagen

Modules covered:

- Health and Disease September- December 2006
- Health Care Systems in Low income countries-January- March 2006
- HIV/AIDS Prevention, Control, Care & Advocacy: March- April 2006
- Thesis – Assessment of quality of care of children aged two months to five years in selected first level facilities using IMCI guidelines in Mongu District in Zambia May- July 2006

Undergraduate 1995 Bachelor's Degree Medicine, Surgery

Medicine, Surgery, Paediatrics, Obstetrics and Gynaecology, Community Medicine, Psychiatry, Forensic Medicine, Medical Subspecialties (Dermatology, STIs and Radiology) and Surgical Subspecialties (Ophthalmology, Anesthesia)

1993 Bachelor's Degree Human Biology

Anatomy, Physiology, Biochemistry, Psychology, Microbiology, Pathology, Pharmacology, Biology, Physics, Chemistry, Mathematics

1. Employment History

Management

Child Health Specialist (2004 to date)

Currently working as the Child Health Specialist at the Ministry of Health coordinating implementation of Child Survival Programs including Micronutrient Supplementation, the Expanded Program of Immunisation (EPI), Integrated Management of Childhood Illness (IMCI) and Paediatric HIV care services. The purpose of the job is to coordinate and manage child survival and child health programmes in order to reduce national child morbidity and mortality.

Chairs the National Technical Working Group for Child Survival whose membership includes bilateral and multilateral cooperating partners and Civil Society Organisations and coordinates various child health partners.

Heads the Secretariat for the Interagency Coordinating Committee for Maternal Newborn and Child Health chaired by the Minister of Health.

Provides leadership for the implementation of child survival programs at all levels through coordination of planning, supervision, partner collaboration and mobilisation of resources.

The key result areas for the job title are:

- **Policy**: regular coordination of formulation, review and implementation of policies related to child survival and child health,
- **Research**: regular coordination of research in child health in order to generate information,
- **M&E:** periodic monitoring and evaluation of child health programmes in order to implement appropriate interventions,
- **Information and management systems**: Coordination of timely and accurately updating of health information on child health data base in order to facilitate efficient storage and retrieval, Budget plans: timely prepares of draft budget plans in order to facilitate acquisition of financial resources,
- **Advisory services:** Coordination and timely provision of advisory services on Integrated Management of Childhood Illness, Expanded Program of Immunisation, Paediatric HIV, services in order to facilitate decision making in the unit,
- **Supervision:** effective supervision of human and material resources in order to facilitate the attainment of the objectives of the unit,
- **Performance management**: Coordination and development of work plans in order to monitor and evaluate performance in the unit.

Acting Provincial Health Director (Jan 2003-Jun2003)

Responsibilities included overseeing and coordinating the public health sector activities in the Province, ensuring the health service delivery conformity to national plans, provision of Technical support for identification and problem solving through to districts and hospitals in the Province.
Monitoring of health performance indicators and provision of quality service delivery. Provision of updates from the national level to districts in line with international norms and practices.
Management of human resources for districts in order to ensure service delivery.

Executive Director (2001-2004)

Executive Director for second level hospital (273 bed hospitals). Chief Executive responsible for the oversight and coordination of second level service delivery and care at the institution.
Planning, management of resources and monitoring implementation of planned activities as well as health indicators as well as identifying training needs and capacity building of staff for improved service delivery.

2. Skills and experience

Policy and Guidelines development

Provides technical advice and leadership and oversight to the development of key policy documents (Child Health Policy, School Health and Nutrition Policy, Integrated Management of Childhood Illness (IMCI) and Expanded Program of Immunisation (EPI), Strategic Planning and Policy Guidelines and National HIV/AIDS Policy.

Chairs the Child Health Technical Working Group and committees on policy and guidelines development for Child Survival (EPI and IMCI), ensures implementation and reviews.

Facilitates the updating and finalisation of national policy documents and guidelines/manuals for implementation of facility and community IMCI and EPI. Has been involved in the strengthening of programs for newborn care and linkages with paediatric ART programs.

Health Systems

Technical leadership for effective implementation of health systems for Child Survival including health management information system, Public-Private Partnerships and Health Services Planning.

Spearheads health systems strengthening for Maternal Newborn and Child Health through Child Survival partners in the country through proposal development.

Provided support and supervised surveyors in the Ministry of Health JICA supported Health facility census 2005-2006.

Immunization Related Highlights

Member of the GAVI Independent Review Committee for Monitoring responsible for evaluating country performance on Immunisation achievements (2007 to date) and also the Independent review committee for Vaccine Investment Strategy providing advise on future vaccine investment (2008).
Head of Secretariat for the Inter Agency Coordination Committee for Zambia coordinating meetings, providing technical updates on Child Health and coordinating follow up recommendations especially EPI and IMCI.

Contributions to EPI action and strategic plans, planning and reviews of Child Health Weeks and EPI campaigns, strengthening of national EPI information system for Zambia.

Presentations and session chairing at the South African Vaccination Immunisation Symposium (SAVIC), Pretoria, South Africa, 2008.

Co-ordination and leadership for the GAVI Health systems proposal writing for Strengthening Immunisation services (2006)

IMCI related highlights

Chairs the IMCI technical working Group which coordinates the implementation of IMCI and during tenure of office the country has scaled up implementation of IMCI coverage from 38 to 72 districts.

Chairs processes for and provides leadership in the adaptation processes and updating of IMCI guidelines and training materials including supporting Ministry of Health Botswana in their adaptation process of IMCI training materials.

National facilitator for IMCI (facility and community)

Coordinated and provided guidance in implementation of the 2008 Health facility Survey.

Planning, programming and reviews

Coordinates regular joint annual child survival reviews with the use of evidence for child survival to inform programming. Provides guidance to Provinces and Districts in the annual work plans. Also provided technical leadership in formulating the National Health Strategic Plan Child Health chapter 2006-2010 for Zambia.

Provided leadership in the writing of the report for the 2007 Child Health chapter for the Zambia Demographic and Health Survey.

One of key facilitators and organisors of the first Zambia Countdown towards 2015 for MNCH MDGs in 2008.

3. Meetings/ Workshops / Trainings attended

- Results Based Financing: Attended the first Trainer of Trainers for RBF in Zambia, Livingstone June 2011.
- 65th World Health Assembly, Geneva, May 2011.
- Comprehensive Multi- Year Plan and Global Annual Progress Report writing workshop, April 2011, Arusha.
- 66th WHO Regional Committee meeting for health Ministers, Equatorial Guinea, September, 2010
- Rota Symposium, Johannesburg, July 2010
- Child Health Forum, Uganda, March 2010
- Technical Advisory Group Meeting on Polio, Angola, February, 2010

- 2nd Regional Vaccinology Course, Nairobi, Kenya, 2008
- Improving Maternal and Infant Health Care Service in Zambia and Zimbabwe, Seoul, Korea, 2007
- WHO Essential Newborn Care Training Course, Lusaka, Zambia, 2007
- Emergency Triage, Assessment and Treatment Training, Lusaka, Zambia, 2007
- Marginal Budgeting for Bottlenecks Training, Kigali, Rwanda, 2006
- Training workshop for Project managers in Home Based Newborn Care, Gadchirolli, India, 2006
- National Facilitator' Training for Planning for Implementation for Family and Community Component of Integrated Management of Childhood Illness, Lusaka, Zambia, 2005
- Facilitators training In IMCI Case Management Training February 2005
- EPI Managers Meeting, Windhoek, Namibia, March 2005
- World Health Assembly, Geneva, Switzerland, May 2005
- Middle Level Management for EPI Managers, Cape Town, South Africa October 2004
- Abridged IMCI Case Management training for Physicians December 2004
- Integrated Management of Childhood Illness (IMCI) focal points meeting, Nairobi- Kenya, June 2004
- Joint Malaria and IMCI Meeting, Maputo, Mozambique September 2004
- Leadership and Management Workshop for Executive Directors Zambia, 2002
- Post Abortion Care Training Workshop for Managers, Zambia, 2002
- Management Skills Workshop for Senior Managers-, Zambia 2000
- ICASA Conference- Lusaka 1999

Papers written or contributed

- Child Health Situation Analysis Zambia 2004
- Child Health Chapter ZDHS 2007,

Computer literacy

Microsoft Windows, MS Word, Excel, EPI-info,

Community Activities and interests

Member: Marriage Enrichment Fellowship.
Gardening, music, and Sports

4. Referees

1. Dr. Victor Mukonka,
   Presidential Housing Initiative
   Lusaka,
   Tel: +260-977 844 754
   Email: vmukonka@gmail.com

2. Dr. Olusengun A. Babaniyi, WHO Representative, World Health Organisation, UN Annex Building, Cnr Andrew Mwenya/Beit Rds.
   P.O. Box 32346, Rhodespark
   Lusaka, Zambia.
   Tel: +260-211-255322/398/336
   Email: babaniyio@zm.afro.who.int

3. Dr. Bola Oyeledun, Country Director, ICAP Nigeria Office, Plot 1120 Kikuyu Close off Nairobi Street, Wuse 2 Abuja.
   Tel: +234 807 749 2515
   +234 802 291 4922
   Email: bolanle3@gmail.com

DR SIMON MUTEMBO
C/O Provincial Medical Office, P. O. Box 60206, Livingstone, Zambia

PERSONAL DETAILS
Year of birth: 12th April, 1975
Languages: English
Sex: Male
Marital status: Married
Nationality: Zambian
Profession: Medical Doctor

EDUCATION

1996-2002	Bachelor of Science in Human Biology<br>University of Zambia
2003-2005	Bachelor of Science in Human Biology<br>University of Zambia
2010-2011	HIV Policy and Prevention<br>Emory University
2011-2012	Master of Public Health (epidemiology)<br>University of Georgia

K**EY COMPETENCES**

- Practical knowledge of community health programs and outreach services
- Proficient in the clinical and program management of HIV, Tuberculosis and Sexually Transmitted Diseases.
- Experience in the management and coordination of maternal and child health programs
- Ability to independently present complex information to various levels of health care providers
- Strong ability to design research, collect research data and analyze it qualitatively and quantitatively.
- Strong experience in developing the district health annual action plans
- Proven experience to work with and coordinate the activities of various partner organizations
- Familiar with UNICEF and PEPFAR program financial management regulations
- Clinical competence in Emergency Obstetric and neonatal care.
- Conversant with Microsoft applications such as word, excel, access and power point.
- Competent in the following statistical software: SAS, STATA and Epinfo.

PROFESIONAL EXPERIENCE

08/2008 to date **Ministry of Health, Provincial Medical office, Southern Province**

Job title: Clinical Care Specialist
Job profile: supervise and coordinate the implementation of clinical care and public health services in both governmental and non-governmental institutions.

Specific duties:

- Design and develop clinical and public health programs for communicable and non-communicable diseases
- Coordinate the implementation of maternal and child health programs in 11 districts
- Monitor and Evaluate all Health care Services for Performance Improvement and quality assurance
- Analyze performance indicators for the districts, provide feedback and report to the Ministry of Health headquarters
- Provide technical support supervision to the different levels of health care guaranteeing adherence to technical guidelines
- Formulate the annual provincial action plan in line with the National Mid Term
- Expenditure Frame Work (MTEF) and the National Health Strategic Plan
- Supervise the programming and the implementation of Paediatric Antiretroviral treatment, Adult Antiretroviral treatment, male circumcision and Sexually Transmitted Diseases programs
- Take part in the clinical care of patients and provide technical support and mentorship to the district health management team staff.
- Coordinate the programing and implementation of the Center for Disease Control and prevention (CDC)/Provincial Medical office collaborative Agreement for HIV and TB prevention and treatment

Achievements

- Conducted a baseline survey for long term family planning and established 10 new family planning clinics in 5 districts
- Coordinated the scale up of comprehensive EmONC sites from 2 to 4 and basic EmONC sites from 8 to 16 in southern province
- Trained district managers in Safe Motherhood and lead the setting up of Safe Motherhood Action Groups in all the 11 districts in Southern Province
- Participated in the institutionalizing of provider initiated counseling and testing in the pediatric department at Livingstone General Hospital which has resulted in the increase of the acceptance of Counseling and testing rate of over 97.3% from less than 40% over a period of 2 years
- Provided oversight for the establishment of the Infant and Young Child Feeding programs in the 4 districts with the highest prevalence of chronic malnutrition in southern province
- Piloted the concept of making Early Infant Diagnosis part of the package of Child Health week activities in 3 districts in Southern Province

- Provided oversight and conceptualized the provision of mobile HIV services in 3 rural districts in the Southern Province of Zambia.
- Scaled up the provision of adult pediatric and adult Anti-Retroviral Treatment and was able to beat the provincial target by 5 000 in 2009.

12/2007-08/2008 **Ministry of Health, Livingstone District Health Management Team**
Job Title: District Director of Health
Job profile: Provide leadership and overall guidance on the delivery of public health service in the district including financial management and control.

Specific duties

- Provided leadership and oversight to over 250 health professionals in the district
- Coordinated and oversaw the provision of public health services for the district with a population of over 120,000 people
- Responsible for daily care of patients while maintaining compliance to national treatment guidelines
- Administered the District Health Management team finances including donor and government grants
- Prepared operational and statistical reports in review of the district clinical and public health performance indicators.
- Establish and maintain productive relationships with stakeholders including the local community and donors
- Attend clinic and provide mentorship to other health care providers

Achievements

- Established two (2) Antiretroviral Treatment clinics in order to scale up the provision of treatment and care to HIV/AIDS patients
- Implemented the Indoor Residue Spraying program and reached a 97% coverage in 2008
- Instituted the home treatment of malaria program in all the catchment areas
- Coordinated the child health week programs resulting in the district annual immunization coverage of 86% in 2008

11/2006-12/2007 **Ministry of Health, Livingstone General Hospital**

Job title: Senior Resident Medical Officer
Job Profile: Involves hands on clinical care of patients, interpreting laboratory and radiological investigations to ensure appropriate case management of patients**.**

Specific duties

- Developed emergence treatment guidelines for the department of obstetrics
- Participated in the development of Provide Initiated Counseling and Testing in the department of Pediatrics and the department of Obstetrics/Gynecology

- Clinical mentorship of medical licentiate interns, student nurses and clinical officers

04/2005-10/2006 Ministry of Health, Ndola Central Hospital

Job title: Junior Resident Medical Officer
Job Profile: Involves hands on clinical care of patients, interpreting laboratory and radiological investigations to ensure appropriate case management of patients**.** This is an internship position and the practice of is done under supervision of a consultant until after certification at the end of 18 months.

Specific duties

- Conducted clinics and ward rounds in the 4 major departments of the hospital-surgery, internal medicine, pediatrics, obstetrics and gynecology.
- Lectured a class of 30 midwifery students at the Ndola School of midwifery
- Participated as a scientific officer in the clinical assessment of the study subjects who were enrolled in the Artemisinin based Combination therapy study at the ***Tropical Diseases Research Center.***

VOLUNTEER WORK

01/2008-06/2010 Gospel Outreach Church, CHRESO Ministries Anti-Retroviral Treatment Clinic
Job title: Site Physician
Specific duties

- Provided technical expertise in compliance with the Medical Council of Zambia accreditation regulations to enable the establishment of the clinic which is part of the response by the church to HIV/AIDS
- Oversaw the clinical care of patients and enrolled over 1,600 HIV positive patients into care within a period of 2 years

06/2005-08/2006 Buseko Orphanage, Ndola
Job title: Volunteer

- Attended to orphaned children staying at the orphanage and arranged for the referral of those that needed hospital care

PUBLICATIONS

Mutanga J N, Raymond J, Towle M S, **Mutembo S**, Fubisha R C, Lule F, Muhe L. Institutionalizing provider initiated counselling and testing for children: An observational case study from Zambia. PlosONE 2012;7(4)

Mutembo S, "Health care and HIV/AIDS in Africa" (Book chapter in Africa continental complexities)

HONORS AND AWARDS

Humphrey Fellowship, 2010-2011, Emory University
Program Focus: HIV policy and Prevention
Program goals:

- To develop qualitative and quantitative research skills
- Improve on the skills and knowledge on HIV program management
- Develop behavioral change communication skills.
- Develop strategic cultural and professional relationships for future scientific collaboration and development.

05/2010 JICA exchange program- Attended a 3 weeks course in Tokyo, Japan on Health Systems with a focus on the provision of Mobile and Outreach Health Services.

04/2008 North Cape/LDHM exchange program- Attended a leadership workshop Oslo, Norway for 2 weeks and developed an agreement for an exchange program between the Livingstone district Health Management team and the North cape community in Norway. The Peace corps of Norway provide sponsorship for the program were 2 health workers from Livingstone go for practical experience in the north cape in exchange for 2 occupational therapist from Norway.

OTHER TASKS AND LEADERSHIP ROLES

2001-2002 University of Zambia Medical School Students Association Vice President and was nominated to the University of Zambia General Senate Committee as a student representative.

CERTIFICATIONS

Certificate of attendance in:

- Paediatric Anti-Retroviral Treatment
- Adult Anti-Retroviral Treatment and management of Opportunistic Infections
- Infant and Young child Feeding
- Integrated Management of Severe Malnutrition
- Clinical Training Skills
- Performance Improvement Approach
- Integrated Management of childhood illnesses

PROFESSIONAL MEMBERSHIP

Medical Council of Zambia; subscription membership number 03/4910
Zambia Medical Association

REFEREES

1. Dr Lutangu Alisheke,
Provincial Health Director-southern Province,
P.O. Box 60206,
Livingstone, Zambia. Tel:
00260955205000
Email: lalisheke@yahoo.co.uk

2. Christopher Whalen
Earnest Corn Professor of Infectious Diseases
B. S. Miller Hall Room 130
Health Sciences Campus
University of Georgia
Athens, GA 30602
Phone: 706 542 0468
Email: ccwhalen@uga.edu

3. Lawrence .H. Marum, MD, FAAP, MPH
Country Director, CDC Zambia
Mailing address (US mail):
Unit 2310 Box 0057
DPO, AE 09816-0057
Phone (Zambia): +260 977 770 958
Email: LMarum@cdc.gov

4. Dr Philip Brachman
Professor of Public Health
Rollins School of Public Health
1518 Clifton Road,
CNR Building 7th Floor
Atlanta GA 30322
Telephone: +1 404 727 0199 Email:
pbrachm@emory.edu

DR. GODFREY BIEMBA: – CURRICULUM VITAE

A. Personal information

Name: Dr. Godfrey Biemba

Sex: Male

Date of birth: 16th December 1958

Address: Zambia Centre for Applied Health Research and Development (ZCAHRD)
4649 Beit road, Rhodes Park, Box 30910, Lusaka.

Email: biemba@bu.edu
godfreybiemba@yahoo.co.uk

A. Personal Statement and Summary of Qualifications, Skills, and Experience

I am a clinical and public health research scientist by training and practice with a special focus and interest on health systems research, evaluation of health programs, malaria and HIV related research. For the past 21 years, I have been involved, at various times, in public health program monitoring and evaluation, and clinical research study design, implementation, analysis and reporting. I am currently the Principal Investigator evaluating the health impact of the Zambian Community Health Assistant (CHA) pilot program being implemented by the Zambian MOH and MCDMCH. I was part of the consulting team to appraise the Zambia National Health Strategic Plan in 2000. I was also part of the consulting team for the Joint Appraisal Mission, EU/DANIDA/DFID: Mid-Term Review of the Zambia Integrated Health Program, March/April, 2001.

In addition to my medical degree, hold a M.Sc. in Public Health in Developing Countries from London School of Hygiene and Tropical Medicine. I have been trained in Project management, research methodologies, health systems and health systems research. I have trained program officers in Monitoring and Evaluation of Programs in Mozambique. I have trained and mentored health workers in health systems research, as well as research methodologies, Good Clinical Practice (GCP) and research ethics.

I am currently a Research Assistant Professor of International Health at the Boston University School of Public Health and Country Director of Boston University's Zambia Centre for Applied Health Research and Development (ZCAHRD). Before becoming Country Director for ZCAHRD, I was Deputy Director for the USAID-funded Orphans and Vulnerable Children Comprehensive Action Research (OVC-CARE) Project, which was implemented by BU's Center for Global Health and Development (CGHD). The project covered all the original 15 PEPFAR supported countries and involved various research projects from formative to applied research, program evaluations and costing. Apart from being responsible for the management of all the research activities globally, I conducted research situation analyses on OVC in Zambia, Kenya, Namibia, Viet Nam, and Nigeria in collaboration with local research institutions in these countries. Before this I carried out extensive literature review on research done on OVC. Apart from working with colleagues on a number of research projects, I was recently the Principal (PI) of a project to evaluate the impact of OVC programs in Mozambique.

Before moving to Boston, I was Deputy Director for Epidemiology and Disease Control at the Ministry of Health Headquarters in Zambia from 2007 to 2008. During this time I was responsible for coordinating all health related research activities in Zambia, as well the development of health research policies and guidelines. Before joining the Zambian Ministry of Health, I was the Chairperson of the National Health Research Advisory Committee, responsible for providing technical support and advice on all health related research in the Country. While serving as Chairman of this important committee, I had a full time job as Executive Director of the Churches Health Association of Zambia (CHAZ), which is responsible for the provision of over 50% of health service delivery in rural Zambia.

B. Educational and Professional Qualifications

- Master's degree in Public Health in Developing Countries (London School of Hygiene and Tropical Medicine, 2003)
- Bachelor of Medicine, Bachelor of Surgery (University of Zambia, 1985)
- Bachelor of Science, Human Biology(University of Zambia, 1982)
- Diploma in Tropical Medicine and Hygiene (Liverpool, 1994)
- Certificate in Health Systems Development (Uppsala Sweden, 2000)

- Diploma in Development and Management of NGOs (Galilee College, Israel, 1999)
- Certificate in Grant Management (PACT Zambia, 1999)
- Certificate in Financial Sustainability for NGOs, SEATS Project in conjunction with INIATIVES INC. and Zambia Family Planning Services (1999)
- Certificate in Health Research Ethics and Good Clinical Practice, AMANET/University of Pretoria (2002).
- Certificate in Ethical Issues in International Health Research (Harvard University, Tufts University, University of Natal, South African Medical Research Council, 2000)
- Certificate in Malaria Research Methodologies (Johns Hopkins School of Public Health/Blair Research Institute Harare, 2001)
- Short Course in International Health, Case Western Reserve University, Ohio, USA (1992)
- Certificate of Attendance, Policy Makers Workshop (2007). South African Cochrane Center in collaboration with MRC South Africa and WHO EVIPNet initiative.

C. Summary of Experience Record

September 2008 to present:

- *Country Director, Boston University Zambia Center for Applied Health Research and Development (ZCAHRD)*– from July 1, 2010 to date
- *Research Assistant Professor at BU Center for Global Health and Development* – from September, 2008.
- *Principal Investigator: Mozambique OVC Research and Evaluation Project – 2010 to 2012.*
- *Principal Investigator: The Community Health Assistant Study-2011 to 2013.*
- *Co-Investigator: Zambia Case Study-The Diarrhoea Global Action Plan Project (DGAP)- Jan-June, 2012.*

July 2007 to August 2008:

Acting Deputy Director, Epidemiology and Disease Control/Communicable Diseases Specialist and National Research Focal Point Person, Ministry of Health Headquarters, Zambia.

Coordinating all Research activities country-wide;

- ***Achievements:***
  - Within eight months, under my leadership, the Ministry of Health has been able to develop the National Health Research Policy, develop the National Health Research Strategic Plan, more than double the budgetary allocation to Health Systems Research, Establish the National Health Research Ethics Committee, develop Guidelines for Research in Traditional Medicine, set up a Research Office, and we are now in the process of establishing a National Health Research Coordinating body.

- In charge of all communicable and Non-communicable diseases;
  - ***Achievements:***
    - Within eight months, the MOH has been able to revive the National Epidemic Preparedness Committee, chaired by the Minister of Health, and its various sub-committees; for the first time, made a budgetary provision for Non-Communicable Diseases (NCDs) and commissioned a baseline survey on the prevalence of NCDs in the country. We have also just commissioned a study on road safety, in collaboration with WHO Country office.

1998-2007

Executive Director, Churches Health Association of Zambia (CHAZ), Lusaka, Zambia.

Served as the Chief Executive Officer of the CHAZ Secretariat, the main implementing body of CHAZ. Responsible for General Strategic direction, Strategic Planning, Financial, Human Resource and General Management of the organization; in charge of resource mobilization and negotiating various contracts with Cooperating Partners as well as suppliers of goods and services;

- ***Achievements:***
  - In nine years, increased the Organization's Annual Budget from less than US$500,000.00 to over US$17million;

- Greatly improved the image of the organization, from a little known to not only nationally, but internationally acclaimed Faith Based Organization;
- Greatly scaled-up Malaria, HIV/AIDS, and TB Programs

1994-1997

Deputy Director, Macha Hospital Malaria Research Institute, Zambia.

- ***Achievements:***
  - Working with Dr. Phil Thuma, the Director of the Institute, who was then based in the USA, I was responsible for research proposal/protocol writing, application for research ethical clearance, logistics, staff training and supervision, budgeting, reporting and write up of research findings for publication.
  - Among other studies, I coordinated and supervised a multi-centre clinical trial of Arte-ether versus Quinine in children with cerebral malaria at the University Teaching Hospital, Lusaka and Macha Hospital in Choma, Zambia. I also designed and supervised an Ethnographic study of malaria in Macha with Dr. T. Milimo, an Anthropologist.
  - Contributed to build the image of the Institute, which has now grown from a small entity operating under the Macha hospital infrastructure, to state of the art Research Infrastructure, conducting high quality malaria research, under a collaborative arrangement with the Johns Hopkins University.

1991 - 1994

Project Manager, Malaria Research, Macha Hospital, Choma Zambia

- Managed the externally funded malaria research project. This included managing personnel, budgeting, logistics, financial and progress reports, etc. And working with the Prof. Phil Thuma and Prof. Victor Gordeuk from USA, wrote project proposals, prepared research protocols, and was in charge of general management of all malaria research activities at Macha hospital in Choma, Zambia.

1994-1997

Deputy Director, The Malaria Institute at Macha

Managing the day to day operations of the newly formed Institute.

1999 to 2007

Research Consultant/Senior Research Associate, The Malaria Institute at Macha.

Assisting the Research Institute in capacity building of research personnel;

- ***Achievements:***
  - Managed to build the research capacity at Macha and a cohesive team of research assistants at the Malaria Institute at Macha.

2004 to 2007

Chairperson, National Health Research Advisory Committee, Ministry of Health, Zambia.

- Leading a team of researchers, Academic Professors, Health experts and Scientists in a team that gives the Ministry of Health Technical Advice on all health related research in Zambia, from policy formulation to research capacity building and research implementation;
  - ***Achievements (As both Chair and Member):***
    - Developed a National Health Research Agenda,
    - Published an Annotated Bibliography of Research done in Zambia,
    - Successfully organized Research Dissemination Conferences every two years,
    - Developed the first Draft of the National Health Research Policy.

1995 to 2008

Member, University of Zambia Research Ethics Committee.

- Reviewing Various Research Proposals for Scientific Rigor and compliance with International Ethical Standards;

2006 to 2008

Part-time lecturer, National Institute of Public Administration (NIPA).

- Lecturing to District Health Managers on "Inequalities and Inequities in Health," as well as "Legal Aspects and Ethical Issues in Medicine,"

1997-1998.

Executive Director, Livingstone Hospital Board of Management. Livingstone, Zambia.

- Chief Executive Officer of the institution, responsible for overall management of a 500 bed second referral (Provincial/General) hospital; in charge of overall financial, human and material resource management as well as overall Strategic Planning and Budgeting.

1992-1994

AIDS Coordinator, Macha Hospital, Choma, Zambia.

- **Achievements:**
    - Managed a hospital and community outreach AIDS program, which included coordinating counseling and health education services at the hospital and the community.
    - Set the foundation for the establishment of a large Community Based HIV/AIDS/OVC Program, now operating at Macha.

1991-1992

Chairperson, AIDS ADVISORY Committee, Churches Medical Association of Zambia (now CHAZ)

- **Achievements:**
    - Led a multi-disciplinary team to give technical advice to the CHAZ Board and Secretariat on all issues regarding HIV and AIDS Programming;

1995-1997

Medical Officer in Charge, Macha Mission Hospital

- In charge of all the Clinical operations of the hospital and part of the management team for the general management of the institution;

1988 -1990.

Co-Director Sentay Medical Services, Livingstone, Zambia.

- Managed a private practice serving almost the whole Livingstone town, Southern Zambia and all the major companies in the area. Managed personnel, general administration, purchasing of all supplies, contracts, etc.

1986-1987.

Senior House Officer, Kitwe Central Hospital, Zambia.

General duty doctor.

D. Health Consultancies

- ***RBM lead Consultant*** for the Kenya Round 8 Malaria Global Fund Proposal:30th April to

30th June 2008

- ***Lead Consultant***, Global Fund Rolling Continuation Channel (RCC), Zambia CCM September to November 2008
- ***Lead Consultant*** for the development of the Global Fund Round 7 Proposal, Zambia CCM-Malaria Component: March-June 2007
- ***Lead Consultant*** - Review of University of Zambia Health Services, University of Zambia, 2000.
- ***Co-Consultant,*** Mid-Term Review of the Zambia Integrated Health Program; USAID ZAMBIA: Part of a consulting team to review a USAID Health Program of support to Zambia: March/April, 2001
- ***Research Consultant*** - NIH sponsored study: "Severe Anaemia and Altered Immune Response," Working with Dr. Phil Thuma, the Director; Responsible for general administrative, logistical and technical conduct of a National Institutes of Health (NIH, USA) funded 5 year research program, including training of research staff from Feb/March, 2001 to 2005
- ***Co-Consultant*** - Joint Appraisal Mission, EU/DANIDA/DFID: Part of a consulting team to appraise the Zambia National Health Strategic Plan: 2000.
- ***Research Study Monitor,*** Malarone study (London School and Macha Collaborative study)

E. Research /Scientific Publications and Works

<u>Selected Peer Reviewed Publications</u>

Malcolm Bryant, Jennifer Beard, Lora Sabin, Mohamad I. Brooks, Nancy Scott, Bruce A. Larson, Godfrey Biemba, Candace Miller, and Jonathon Simon
PEPFAR's Support For Orphans And Vulnerable Children: Some Beneficial Effects, But Too Little Data, And Programs Spread Thin. HEALTH AFFAIRS 31, NO. 7 (2012): –
©2012.

Jennifer Beard, **Godfrey Biemba**, **Mohamad I Brooks, et al.**

Children of female sex workers and drug users: a review of vulnerability, resilience and family centred models of care. Journal of the International AIDS Society 2010, **13**(Suppl 2)**:**S6

Biemba et al. *Prolonged Macrophage Activation and Persistent Anaemia in children with complicated malaria*. Tropical Medicine and International Health, Vol.3 No.1 pp60-65, January, 1998.

Biemba et al. *Severe Anaemia in Zambian Children with Plasmodium falciparum malaria",* Tropical Medicine and International Health, Jan, 2000; 5(1): 9-16

Biemba et al. *Markers of Inflammation in children with severe malarial anaemia*. Tropical Medicine and International Health, April, 2000; 5(4): 256-262

Marjolein Dieleman, Godfrey Biemba, et al. (2006). *We are also dying like any other people, we are also people': perceptions of the impact of HIV/AIDS on health workers in two districts in Zambia*. Health Policy and Planning 2007 22(3):139-148

V Gordeuk, P Thuma, G Brittenham, C McLaren, D Parry, A Backenstose, G Biemba, R Msiska, L Holmes, E McKinley, and et al.
Effect of iron chelation therapy on recovery from deep coma in children with cerebral malaria New England Journal of Medicine (Nov 19, 1992*) Vol* 327:1473-1477, No. 21.

Victor R. Gordeuk, Philip E. Thuma, Gary M. Brittenham, Godfrey Biemba, et al.
Iron Chelation as a chemotherapeutic strategy for falciparum malaria. American Journal of Tropical Medicine and Hygiene 48(2), 1993, pp193-197.

Mabeza GF, Moyo VM, Thuma PE, Biemba G. et al. *Predictors of the Severity of Illness on presentation in children with cerebral malaria*. Annals of Tropical Medicine and Parasitology, Vol.89, No.3, 221-228 (1995).

George F. Mabeza, Godfrey Biemba, Victor R. Gordeuk. *Clinical Studies of Iron Chelators in malaria*. Acta Haematologica, 1996; 95:78-86.

Gordeuk VR, Thuma PE, McLaren CE, Biemba G. et al. *Transferrin Saturation and Recovery from coma in Cerebral malaria*. Blood, Vol.85, No.11, 1995:pp3297-3304.

PE Thuma, GJ Bhat, GF Mabeza, C Osborne, G Biemba et al. *A Randomized Controlled Trial of Artemotil (Beta Arte-ether) in Zambian children with Cerebral malaria*. Am. J. Trop. Med. Hyg., 62(4), 2000, pp. 524-529

Other Unpublished Works

Biemba et al. *Kenya Research Situation Analysis on Orphans and Vulnerable Children, Final Report and Country Brief,* submitted to USAID, 2009 by **Center for Global Health and Development in collaboration with University of Nairobi, Institute for Development Studies**

Biemba et al. *Zambia Research Situation Analysis on Orphans and Vulnerable Children, Final Report and Country Brief,* submitted to USAID, 2009 by **Center for Global Health and Development in collaboration with University of Zambia, Institute for Economic and Social Research**

Biemba et al. *Namibia Research Situation Analysis on Orphans and Vulnerable Children, Final Report and Country Brief,* submitted to USAID, 2009 by **Center for Global Health and Development in collaboration with PharmAccess, Namibia**

Biemba et al. *Nigeria Research Situation Analysis on Orphans and Vulnerable Children, Final Report and Country Brief,* submitted to USAID, 2009 by **Center for Global Health and Development in collaboration with Initiative for Integrated Community Welfare in Nigeria**

Biemba et al. *Vietnam Research Situation Analysis on Orphans and Vulnerable Children, Final Report and Country Brief,* submitted to USAID, 2009 by **BU Center for Global Health and Development in collaboration with Hanoi School of Public Health**

Biemba G. *Added and Invisibility of Religious Health Assets,* **ARHAP Concept Paper, 2007**

Biemba G. *HIV/AIDS Interventions and Strategic approaches in Africa:-*The Zambian Strategy (Paper presented at the Interaction Forum and Washington Week, Washington DC, USA; 2002)

Biemba G. *Multi-sectoral response to HIV/AIDS: the Zambian Example* (Paper presented to M. Sc. Public Health students at the London School of Hygiene and Tropical Medicine, 2003; also earlier presented at the Interaction Forum, Washington DC, USA, 2002)

F. Memberships and Affiliations

i. Member of the Medical Research Council (UK) Data Safety Monitoring Board for the CHAP (Co-trimoxazole study) and CHAPAS studies, up to now
ii. Board Member, The Malaria Institute at Macha (MIAM): 2008 to date
iii. Board Member, ERES Converge, IRB: 2008

iv. Member of the Zambia Forum For Health Research, since 2006 to date
v. Member of the Zambia Health Sector Advisory Group (SAG): 2005-2007
vi. Member, Zambia National Health Research Advisory Committee, 1999 to 2007
vii. Chairperson, Zambia National Health Research Advisory Committee, 2004-2007
viii. Member, University of Zambia School of Medicine, Research and Ethics Committee, 1995 to 2007
ix. Member, Medical Council of Zambia, till 2007
x. Vice-Chairperson of the University of Zambia Health Services Advisory Committee; 2006 to 2007.
xi. Chairperson, Debt Swap for HIV/AIDS Proposal Development (NGOs/Civil Society Group), 1999/2000.
xii. Member, World Bank HIV/AIDS Proposal Development Working Group, 2000/2001
xiii. Member, Poverty Reduction Strategy Paper Health Working Group, 2001
xiv. Member, Advocacy Liaison Group, Debt Relief for HIV/AIDS.
xv. Member, Catholic Commission for Justice and Peace Parliamentary Liaison Program Steering Committee, Debt Relief for HIV/AIDS (representing Civil Society), 2002
xvi. Member, Central Board of Health 1998 to 2004
xvii. Member, National AIDS Council till 2003
xviii. Member, Checke Cultural Writers' Association

Referees

i. Professor Jonathan Simon, Chair, Department of International Health and Director, Center for Global Health and Development, Boston University, 801 Mass Ave, Crosstown Centre, MA02118, USA. Tel: 1 617 414 1262; Email: jonsimon@bu.edu
ii. Professor Chifumbe Chintu, Paediatrician/Haematologist/Oncologist, University of Zambia School of Medicine, P.O. Box 5110, Lusaka.
Mobile: +260 977 883261
Email: cchintu@zamnet.zm
iii. Prof. Phil Thuma, Assistant Professor of Paediatrics, Director, The Malaria Institute at Macha, Choma, Zambia.
Mobile: +260 977 721599
Email: pthuma@machamalaria.org or phil.thuma@macha.org.zm

BIOGRAPHICAL SKETCH

NAME	POSITION TITLE
Hamer, Davidson Howes	Professor of International Health & Medicine<br>Boston U. Schools of Public Health and Medicine<br>Adjunct Professor of Nutrition<br>Tufts University Friedman School of Nutrition Science and Policy
eRA COMMONS USER NAME<br>DHAMER@BU.EDU	

EDUCATION/TRAINING *(Begin with baccalaureate or other initial professional education, such as nursing, and include postdoctoral training.)*

INSTITUTION AND LOCATION	DEGREE *(if applicable)*	YEAR(s)	FIELD OF STUDY
Amherst College Amherst, MA	B.A.	1981	Biology
University of Vermont College of Medicine Burlington, VT	M.D.	1987	Medicine

A. Personal Statement

During the last twenty years, I have been engaged in field research on the epidemiology of and interventions to reduce neonatal and child morbidity and mortality. During this period, I have supervised and provided technical support to >50 studies in developing countries that evaluated interventions for improving neonatal survival as well as the treatment and prevention of malaria, pneumonia, diarrhea, HIV/AIDS, and micronutrient deficiencies. In collaboration with colleagues at the Boston University (BU) Center for Global Health and Development, I have worked closely with scientists at the Zambian Ministry of Health, National Malaria Control Centre, Tropical Diseases Research Centre, and several different NGOs on policy-relevant, applied research that has resulted in important modifications in national malaria treatment guidelines and control policies. As a result of my longstanding commitment to improving the health of Zambian women and children, I moved to Lusaka, Zambia in August 2011. In my current position as the Director of Research and Evaluation for the Zambia Centre for Applied Health Research and Development (ZCAHRD), I work closely with the Ministry of Health at many levels of the health system. My major current projects in Zambia include a large neonatal survival study in Southern Province (the Zambia chlorhexidine application trial); evaluation of a package of community-based interventions designed to reduce neonatal and under 5 child morbidity from common diseases (Lufwanyama Integrated Neonatal and Child Health project); evaluation of a pilot program of community health assistants; and the Saving Mothers Giving Lives/Zambia project. Clinically I have extensive knowledge of vaccines as a result of having directed two different travel clinics in the USA over the last two decades. My combined neonatal health research and in depth knowledge of vaccine immune responses and safety provide me with the background necessary to play a helpful technical role as an Other Significant Contributor for Project Safe Start, which will consist of participating on the Scientific Advisory Group.

B. Positions and Honors

<u>Positions and Employment</u>

1987 - 1990 Internship and Residency Internal Medicine Washington Hospital Center, Washington, D.C. 1990 - 1994 Infectious Diseases Fellowship, Tufts-New England Medical Center (TNEMC)

1994 - 2003 Assistant Professor of Medicine, Tufts University School of Medicine (TUSM)
1994 - 2004 Director, Traveler's Health Service, TNEMC
1996 – 2000 Project Scientist, Applied Research on Child Health Project, Harvard Institute for International Development (HIID)
2001 – 2004 Assistant Professor of Nutrition (secondary appointment), Tufts University Friedman School of Nutrition Science and Policy (FSNSP)
2001 - 2004 Project Scientist, Applied Research on Child Health Project, Center for International Health, Boston University School of Public Health (BUSPH)
2001 - 2004 Adjunct Assistant Professor of International Health, BUSPH
2003 - 2004 Associate Professor of Medicine, TUSM
2004 - 2010 Adjunct Associate Professor of Nutrition, FSNSP
2004 - 2010 Associate Professor of International Health, BUSPH
2005 - 2011 Director, Travel Clinic, Boston Medical Center
2006 - Adjunct Scientist, Nutritional Immunology Dept., Human Nutrition Research Center on Aging
2007 - 2010 Associate Professor of Medicine, Boston University School of Medicine
1/10 - Faculty member, Division of Graduate Medical Sciences, Program in Medical Nutrition Sciences, BUSM
5/10 - Professor of International Health and Medicine, BUSPH and BUSM
6/10 - Adjunct Professor of Nutrition, Tufts University FSNSP
8/11 - Director of Research and Evaluation, ZCAHRD

Additional Professional Experience

2001 - 2009 Member, World Health Organization Young Infant Study Steering Committee
2006 - 2012 Member, Board of Directors of the Nevin Scrimshaw International Nutrition Foundation
2008 - Member, UNICEF/WHO Community Case Management Operational Research Group
2008 - Member, Advisory Committee, Thrasher Research Fund
2011 - President, Clinical Group, American Society of Tropical Medicine and Hygiene
2010 - Chair, Research and Travel Awards Committee, International Society of Travel Medicine
2012 - Member, Intermittent preventive treatment of malaria in pregnancy evaluation review group (IPTp ERG), Global Malaria Program, WHO

Professional Memberships

1994 - Member, Infectious Diseases Society of America
1995 - Member, American Society of Tropical Medicine and Hygiene
1992 - Member, International Society of Travel Medicine
2000 - Fellow, American College of Physicians
2001 - Member, American Academy of HIV Medicine
2007 - Fellow, Infectious Diseases Society of America

Honors

1992 Edward Kass Award for Clinical Excellence in Infectious Diseases Massachusetts Infectious Diseases Society
2001 Merrimack Valley Consumer Advisory Board AIDS Provider Award
2002 University of Vermont College of Medicine Most Recent Alumnus Award
2005, 2007 BUSPH teaching award—spring semester for IH 805 Eradication of Infectious Diseases

C. Selected peer-reviewed publications (Selected from >85 peer-reviewed publications)

Most relevant to the current application (in chronological order)

- **Hamer DH**, Mwanakasale V, MacLeod WB, Chalwe V, Mukwamataba D, Champo D, Mwanayanda L, Chilengi R, Mubikayi L, Mulele CK, Mulenga M, Thea DM, Gill CJ. Two dose versus monthly intermittent preventive treatment of malaria with sulfadoxine-pyrimethamine in HIV-seropositive pregnant Zambian women. J Infect Dis 2007;196:1585-94.
- Gill CJ, MacLeod WB, Mwanakasale V, Chalwe V, Mwanayanda L, Champo D, Mukwamataba D, Chilengi R, Thea DM, **Hamer DH**. Inferiority of single dose sulfadoxine-pyrimethamine intermittent preventive therapy during pregnancy among HIV-positive Zambian women. J Infect Dis 2007;196:1577-84.
- Sipilanyambe N, Simon JL, Chanda P, Olumese P, Snow RW, **Hamer DH**. Changing and implementing new national antimalarial drug policy in Zambia: from chloroquine to artemether-lumefantrine. Malaria J 2008;7:25.
- Yeboah-Antwi K, Pilingana P, Macleod WB, Semrau K, Siazeele K, Kalesha P, Hamainza B, Seidenberg P, Mazimba A, Sabin L, Kamholz K, Thea DM, **Hamer DH**. Community case management of fever due to malaria and pneumonia in children under-five in Zambia: A cluster randomized controlled trial. PLoS Medicine 2010;7:e100340.
- Gill CJ, Phiri-Mazala G, Guerina NG, Kasimba J, Mulenga M, MacLeod WB, Waitolo N, Knapp AB, Mirochnick M, Mazimba A, Fox MP, Sabin LL, Seidenberg P, Simon JL, **Hamer DH**. The Lufwanyama Neonatal Survival Project (LUNESP): a randomized, controlled study of using trained traditional birth attendants to reduce neonatal mortality. Brit Med J 2011;342:d346.

Additional recent publications of importance to the field (in chronological order)

- Zurovac D, Ndhlovu M, Rowe AK, **Hamer DH**, Thea DM, Snow RW. Treatment of paediatric malaria during a period of drug transition to artemether-lumefantrine in Zambia: cross-sectional study. Brit Med J 2005;331:734-7.
- **Hamer DH**, Ndhlovu M, Zurovac D, Fox M, Yeboah-Antwi K, Chanda P, Sipilinyambe N, Simon JL, Snow RW. Does improving coverage of parasitological diagnostic tests change malaria treatment practices? An operational cross-sectional study in Zambia. JAMA 2007;297:2227-2231.
- The Young Infants Clinical Signs Study Group. Clinical signs predicting severe illness in young infants: a multicentre study. Lancet 2008;371:135-42.
- Gill CJ, Mwanakasale V, Fox MP, Chilengi R, Tembo M, Nsofwa M, Chalwe V, Mwananyanda L, Mukwamataba D, Malilwe B, Champo D, Macleod WB, Thea DM, **Hamer DH**. Effect of presumptive cotrimoxazole prophylaxis on pneumococcal colonization rates, seroepidemiology, and antibiotic resistance among Zambian infants. Bull WHO 2008;86:929-38.
- Krezanoski PJ, Comfort AB, **Hamer DH**. Effect of incentives on insecticide-treated bednet use in sub-Saharan Africa: a cluster randomized trial in Madagascar. Malaria J 2010;9:186.
- Zurovac D, Sudoi RK, Akhwale WS, Ndiritu M, **Hamer DH**, Rowe AK, Snow RW. Mobile phone text-message reminders as a tool to improve adherence to malaria guidelines: a cluster randomized trial. Lancet 2011; DOI:10.1016/S0140-6736(11)60783-6.
- Gill CJ, Guerina NG, Knapp AB, **Hamer DH**. Training Zambian traditional birth attendants to reduce neonatal mortality in the Lufwanyama Neonatal Survival Project (LUNESP). Int J Gyn Obstetrics 2012, doi:10.1016/j.ijgo.2012.02.012.
- Sabin LL, Knapp AB, MacLeod WB, Phiri-Mazala G, Kasimba J, **Hamer DH**, Gill CJ. Costs

and cost-effectiveness of training traditional birth attendants to reduce neonatal mortality in the Lufwanyama Neonatal Survival Study (LUNESP). PLoS One 2012;7:e35560.

- **Hamer DH**, Brooks ET, Semrau K, Pilingana P, MacLeod WB, Siazeele K, Sabin LL, Thea DM, Yeboah-Antwi K. Quality and safety of integrated community case management of malaria using rapid diagnostic tests and pneumonia by community health workers. Pathogens & Global Health 2012;106;32-39.
- De Sousa A, Tiedje KE, Recht J, Bjelic I, Marsh DR, **Hamer DH**. Policy and implementation status of community case management of childhood illnesses in the countdown priority countries. Bulletin of the WHO 2012;90:183-90.

D. Research Support

<u>Ongoing Research</u>

Bill and Melinda Gates Foundation Hamer (PI) 11/15/09 – 11/15/13
Zambia Chlorhexidine Application Trial
The goal of this cluster, randomized controlled trial is to evaluate the impact of 4% chlorhexidine cord washes on neonatal mortality and omphalitis in comparison to dry cord care in the Southern Province of Zambia.

Bill and Melinda Gates Foundation Hamer (co-PI)
8/15/12 – 8/14/14
Cohort harmonization (supplement to ZamCAT)
This is a series of supplemental epidemiological studies on maternal and neonatal health that will be conducted in collaboration with 9 other sites in South Asia and Africa.

Centers for Disease Control and Prevention 1U2GPS001418-0 Thea (PI)
10/1/11 – 5/31/13
Saving Mothers Giving Life/Zambia
Boston University (BU), through its affiliated Zambian partner, the Zambia Centre for Applied Health Research and Development (ZCAHRD), will implement with the Southern Province Medical Office (SPMO) a package of interventions and health facility enhancements in Kalomo District, Zambia that will improve the access and quality of maternal antenatal, intrapartum and postnatal care services as well as the community demand for these services that will result in a measureable decrease in maternal mortality.
Role: Co-investigator

NIAID R01 HD057941-01 Fawzi (PI) 11/1/09 – 10/31/14
Malaria in pregnancy: nutrition and immunology effects
The goal of this randomized, controlled trial is to evaluate the protective efficacy of zinc and/or vitamin A for reducing the burden of placental malaria in pregnant women in Tanzania.
Role: Co-investigator

USAID-M-OAA-GH-09-298 Yeboah-Antwi (PI) 10/1/09 – 3/31/12
Lufwanyama Integrated Neonatal and Child Survival Project
FY 2009 Child Survival and Health Program Innovation Award cooperative agreement (Save the Children)
The goal of this project is to support operational research around the scaling up of an integrated community-based program designed to reduce neonatal and child mortality in rural Zambia.
Role: Co-investigator on subcontract to BU from Save the Children

Past Research Support

Centers for Diseases Control and Prevention Barnett/Hamer (co-PI) 10/1/07 – 9/30/11
Boston Area Travel Medicine Network
The goal of this multi-site project is focused on evaluating the knowledge, attitudes, and practices of high-risk travelers and improving our understanding of travel medicine practices of general practitioners.

Thrasher Research Foundation Sempértegui (PI)
8/1/07 – 7/31/10
Effects of zinc as an adjunct to the treatment of pneumonia in young children in Ecuador
The major goals of this project were to evaluate the impact of short-term zinc supplements on pneumonia-related outcomes and the differential impact of zinc on specific pneumonia pathogens.
Role: Co-PI

Child and Family Applied Research Project Simon (PI)
10/1/03-9/30/09
United States Agency for International Development
Role: Co-PI
Within this cooperative agreement were the following studies:

1) "Burden of disease due to malaria in pregnancy in east India (Jharkhand)" 10/1/05-3/31/07
   The major goals of this project were to assess the prevalence of malaria among pregnant women and their associated birth outcomes in Gumla District in Jharkhand State, India.
   Role: PI
2) "Reducing neonatal mortality in resource-constrained environments" 3/1/05-10/31/08
   The major goal of this project was to study the feasibility, effectiveness, and cost-effectiveness of a package of interventions in reducing neonatal mortality in rural Zambia.
   Role: PI
3) Community-based management of fever due to malaria and pneumonia"
   10/1/05 – 9/30/08
   This was a cluster randomized, community-based study of the management of fever due to malaria and pneumonia by community health workers in Zambia.
   Role: Co-investigator (PI Yeboah-Antwi)

NICHD R03 HD52167-01 (part of the Indo-U.S. Collaborative Network) Hamer (PI)
9/1/06 – 8/31/08
"Burden of disease due to malaria in pregnancy in Chhattisgarh, India"
The major goals of this project were to assess the burden of disease due to malaria among pregnant women and their associated birth outcomes in Chhattisgarh State, India.

Elizabeth A. McCarthy
P.O. Box 51071, Ridgeway +260 979 381 709
Lusaka, Zambia emccarthy@clintonhealthaccess.org

EDUCATION:

Johns Hopkins Bloomberg School of Public Health, Master of Public Health, May 2002.

Tufts University, Bachelor of Arts, International Relations and Economics, May 1995.

- **Tufts-in-Paris**, study abroad including courses at Institut d'Etudes Politiques and Sorbonne.

EXPERIENCE:

CLINTON HEALTH ACCESS INITIATIVE, Lusaka, Zambia

Director, Applied Analytics Team **2011-present**

- Develop and implement a business and operational strategy for the Applied Analytics Team
- Catalyze organization-wide use of evidence to guide global health program and policy decisions
- Supervise team of Senior Research Associates, Research Associates, and Volunteers and ensure quality of team's work
- Grow team's portfolio of projects and available funding to accomplish its goals and objectives
- Provide technical expertise in support to projects with potential for transformational change

Operations Research Lead, Applied Analytics Team **2010-2011**

- Design decision making tools that use scientific evidence to influence policy and programming
- Analyze cost and cost-effectiveness of critical activities geared towards the prevention of mother to child transmission (PMTCT) and pediatric HIV care and treatment

CLINTON HEALTH ACCESS INITIATIVE, Boston, Massachusetts

Operations Research Lead, Center for Strategic Health Operations Research (CSHOR) **2009-2010**

- Develop and apply complex mathematical models to global health challenges.
- Lead teams conducting quantitative analysis to inform policy and program decision-making.
- Liaise with managers of country and global teams to set operations research agenda.

Director of Policy Analysis, Center for Strategic Health Operations Research (CSHOR) **2006-2009**

- Conducted quantitative operations research analyses related to HIV treatment programs and health systems strengthening in resource-limited settings.
- Disseminated cross-cutting policy analyses based on operations research.
- Contributed to CSHOR strategic direction, planning, and management.
- Managed CSHOR policy team.

Senior Research Associate, Center for Strategic Health Operations Research (CSHOR) **2005-2006**

- Conducted and disseminated quantitative analyses to inform new and ongoing Clinton Foundation initiatives.

BURMA BORDER PROJECTS (BBP), Mae Sot, Thailand

Consultant **2004**

- Evaluated the effectiveness of BBP's mental health program along the Thai-Burma border.
- Developed interview guides, trained a local team to conduct interviews, supervised data collection, coded and analyzed translated transcripts, and disseminated findings.

CENTERS FOR DISEASE CONTROL AND PREVENTION (CDC), GLOBAL AIDS PROGRAM, Atlanta, GA

Consultant, Most-at-Risk Populations Team **2003-2004**

- Designed International Rapid Assessment, Response and Evaluation (I-RARE) Methods training curriculum used to qualitatively assess and analyze HIV risk among drug users.

Research Fellow, Modeling and Reinforcement to Combat HIV/AIDS (MARCH) Team **2002-2003**

- Collaborated with partners in Botswana, Ethiopia and Zimbabwe to implement national radio serial dramas designed to model realistic HIV behavior change.
- Analyzed quantitative behavioral data measuring impact of radio serial drama on HIV behavior change in Ethiopia.
- Designed and piloted training curriculum to launch community activities in Zimbabwe to reinforce behavior change messages communicated in radio serial dramas.

JOHNS HOPKINS BLOOMBERG SCHOOL OF PUBLIC HEALTH, Baltimore, MD
Research Assistant **2001-2002**

- Coded and synthesized intervention design and results data from literature on HIV/AIDS behavioral interventions in developing countries as part of the World Health Organization's Synthesizing Intervention Effectiveness Project.

PEACE CORPS, MADAGASCAR
Health Program Specialist **1999-2000**

- Trained 29 Peace Corps Volunteers in technical skills necessary for 2-year service in the health sector during their 12 week intensive pre-service training.
- Supervised and provided technical assistance, guidance and ongoing training to 45 health volunteers.

DEPARTMENT OF COMMERCE, COMMERCIAL LAW DEVELOPMENT PROGRAM, Washington, DC
International Program Assistant **1998-1999**

- Managed content, design and execution of commercial law development technical assistance activities between partners in the United States and Egypt.

PEACE CORPS, MADAGASCAR
Peace Corps Health Volunteer **1995-1997**

- Conducted maternal and child health survey, analyzed data, and disseminated results.
- Communicated health messages to target communities using non-formal education techniques.
- Facilitated an income-generating bee-keeping project with 67 Malagasy women from 7 women's groups that incorporated bee-keeping, small business management, and micro-credit skills.
- Taught teachers innovative and creative methods of including health messages in the classroom.
- Organized and led a regional girls' camp designed to encourage leadership and strengthen English and health knowledge among participants.
- Negotiated national distribution of 1 million matchboxes encouraging childhood immunization.

PUBLICATIONS:

Tjoa A, Kapihya M, Libetwa M, Schroder K, Scott C, Lee J, **McCarthy E**. Meeting human resources for health staffing goals by 2018: a quantitative analysis of policy options in Zambia. *Human Resources for Health* 2010, 8:15.

Tjoa A, Kapihya M, Libetwa M, Lee J, Pattinson C, **McCarthy E**, Schroder K. Estimating the Feasibility and Costs of Doubling Health Training Institution Graduate Output in Zambia: Planning

through Individual School Assessments. *Human Resources for Health* (accepted in principle, under review by the journal's editorial team).

McCarthy E, O'Brien M, Rodriguez W. Training and HIV-treatment scale-up: establishing an implementation research agenda. *PLoS Med* 2006;3(7):e304.

O'Brien M, **McCarthy E**. HIV Global Epidemiology. In: Libman H, Makadon HJ, eds. HIV, Third Edition. Philadelphia: American College of Physicians, 2007.

McCarthy E, Sliney A, O'Brien M. Popular Training Methodologies and Applications. In: Marlink R, Teitelman S, eds. From the Ground Up: A Guide to Building Comprehensive HIV/AIDS Care Programs in Resource-Limited Settings. Washington, DC: Elizabeth Glaser Pediatric AIDS Foundation, 2008.

PRESENTATIONS:

McCarthy E, Shelley K, Romano S. Achieving elimination of new HIV infections among children by 2015 and keeping their mothers alive in Zambia and globally requires dramatically improving receipt of all critical services for pregnant women and their infants, from the beginning of pregnanc. : 19th International AIDS Conference: Abstract no. WEPE658.

McCarthy E, Ipuge Y, Silaa R, Njau P, Ramadhani A, Koehler E, Sickler J, Essajee S. Money spent on prevention of mother-to-child transmission - a good investment for Tanzania. 6th International AIDS Society Conference: HIV Pathogenesis, Treatment and Prevention (Rome), 2011. Poster MOPE434.

McCarthy E, Ipuge Y, Silaa R, Essajee S. What will it cost to move from the 2006 to 2010 WHO pediatric antiretroviral guidelines in Tanzania? 6th International AIDS Society Conference: HIV Pathogenesis, Treatment and Prevention (Rome), 2011. Poster MOPE458.

McCarthy E, Bweupe M, Worku Y, Michaelis A, Romano S. Modeling for decision-making to achieve virtual elimination of mother-to-child transmission of HIV in Zambia. 6th International AIDS Society Conference: HIV Pathogenesis, Treatment and Prevention (Rome), 2011. Poster MOPE434.

McCarthy E, Ipuge Y, Silaa R, Njau P, Ramadhani A, Essajee S. Cost-effectiveness of Options A and B of the 2010 WHO PMTCT Antiretroviral Guidelines in Tanzania. 6th International AIDS Society Conference: HIV Pathogenesis, Treatment and Prevention (Rome), 2011. Poster TUPE419.

McCarthy E. Tjoa A, Yarrow J, Romano S, Campbell J. Cost and impact of adopting the new WHO guidelines to prevent mother-to-child transmission in Malawi. XVIII International AIDS Conference (Vienna), 2010. Abstract TUPE0930.

Tjoa A, **McCarthy E**, Ellis M. Evaluating task-shifting strategies for PMTCT services in rural Malawi. XVIII International AIDS Conference (Vienna), 2010. Abstract 7507.

Walsh F, Aung M, Lowe J, Clarke M, **McCarthy E**. Maximizing utilization of existing staff time at two HIV treatment sites in Jamaica. XVIII International AIDS Conference (Vienna), 2010. Abstract 10888.

Ellis M, Chapotera G, Wayda B, Tjoa A, Scott C, **McCarthy E**. Scaling up PMTCT services in Malawi: ensuring adequate human resources to meet the demand. 5th IAS Conference on HIV Pathogenesis, Treatment and Prevention (Cape Town), 2009. Abstract TUPED117.

Tjoa A, Kapihya M, Libetwa M, Schroder K, Scott C, Pattinson C, Lee J, **McCarthy E.** Expanding the human resources for health (HRH) workforce in Zambia. 5th IAS Conference on HIV Pathogenesis, Treatment and Prevention (Cape Town), 2009. Abstract TUPED130.

LANGUAGES:

- Proficient in oral and written French.
- Conversational in oral and written Spanish and Malagasy.

BWALYA MOSES KANGWA

Mobile: +260 966/55 880467, E-mail: bwalya03@yahoo.com
Postal Box 50946, Kabwata Baptist Church, Lusaka

EDUCATION

University of Manchester Metropolitan — ***UK***
MSc Strategic Business Management — Studying

Chartered Institute of Management Accountants (CIMA) — ***UK***
CIMA qualified — May 2011

Zambia Institute of Chartered Accountants — ***Zambia***
National Accounting Technician (Natech) — December 2003

EXPERIENCE

CLINTON HEALTH ACCESS INITIATIVE (CHAI) — ***Sept. 2012-Present***
Program Manager (3DE)
Demand-Driven Evaluation for Decisions (3DE) is a DFID threes years funded program that uses impact evaluation to generate evidence which can be useful to drive decision making in the health sector in Zambia. Some of my key responsibilities are;

- Ensuring the project meets its objectives
- Budgeting and financial management of the project
- Using analytics, mathematical modeling, cost effectiveness and analyses of literature
- Managing the process of identifying the questions suitable for the 3DE approach
- Liaising with other organizations or academic institutions that specialize in evaluations
- Offer some technical assistance to the relevant stakeholders in the ministry of health(Zambia)
- Communicating the findings of the 3DEs to relevant stakeholders in the Ministry of Health
- Dissemination of 3DE findings to stakeholders and the global evidence

Zambia AIDSLaw Research and Advocacy Network (ZARAN) — ***Aug.2007- Aug.2012***
Finance & Administration Manager [Senior Level]
I was responsible in managing all the financial and administrative responsibilities of the organization with the assistance of the finance assistant and the Administrative Assistant while reporting to the Executive Director. Some of the key roles involved;

- Managing multiple grants from different funders
- Budget formulation, implementation, monitoring and analysis
- Ensuring full compliance with International Accounting Standards and internal policies
- Preparing various reports including annual financial reports for audit
- Reviewing and analysing reports prepared by the finance assistant
- Payroll administration and ensuring statutory compliance
- Payment reviews and monitoring budget spending
- Managing fixed assets

- Managing IT, procurement, medical scheme and primary liaison with the bank and donors on financial issues

Action International ***June 2005- July 2007***
Accountant [Middle Level]
While reporting to the Finance Manager, my roles involved report preparations, budgeting and variance analysis, bank reconciliations, financial record keeping and a number of administrative roles.

Flying Mission Zambia ***Dec. 2006-June 2007***
***Part-Time Accountant [Middle Level*]**
I was mainly engaged to straighten the financial management and setting out effective systems for the management of financial resources.

COMPUTER SKILLS

▪ Pastel Accounting Package	Advanced
▪ Pastel Payroll Software	Advanced
▪ MS Outlook & Express	Advanced
▪ Dove payroll Software	Advanced
▪ Quick Books	Intermediate
▪ Power Point	Intermediate
▪ MS excel and word	Intermediate
▪ SAP	Basic

OTHER TRAININGS ATTENDED

- 2010 Budgeting and Strategic Planning
- 2010 Funding Proposal writing
- 2008 Cash Forecasting
- 2008 Effective Internal Controls
- 2007 Book Keeping

MEMBERSHIPS

- Full member of the Chartered Global Management Accountant (**CGMA**)
- Associate member of Chartered Institute of Management Accountants (**CIMA**)
- Member of the Zambia Institute of Chartered Accountants (**ZICA**)

4.7 Appendix 7: References

[i] Interview Mr. Clement Phiri, Laboratory Services, Zambian Ministry of Health. November 29th, 2012.
[ii] UNGASS Zambia Country Report, Monitoring the Declaration of Commitment on HIV and AIDS and the Universal Access, Biennial Report. Submitted March 31st, 2012
[iii] GSG Mid-Term Review Meeting Report, Early Infant Diagnosis IATT Laboratory & Child Survival Working Group, EMTCT-IATT, UNICEF. December 6-7, 2012.
[iv] Together we will end AIDS. UNAIDS. 2012.
[v] UNAIDS Progress Report Summary. 2011. [EID coverage rates at the end of 2011 were not reported in the more recent UNAID Progress Report, 2012]
[vi] UNGASS Zambia Country Report
[vii] Newell, ML et al. Mortality of infected and unifected infants born to HIV-infected mothers in Africa: a pooled analysis. Lancet. Oct 2-8;264(9441):1236-43. 2004.
[viii] UNAIDS 2008 Report on the global AIDS epidemic.
[ix] Countdown to Zero, Elimination of New HIV Infections Among Children by 2015 and Keeping Their Mothers Alive, Zambia. UNICEF. < http://www.unicef.org/aids/files/hiv_pmtctfactsheetZambia.pdf>
[x] Interview, Clement Phiri, Laboratory Services, MoH November 29th, 2012
[xi] Interview, Clement Phiri.
[xii] Zambia: WHO and UNICEF estimates of immunization coverage: 2011 revision. PDF file.
[xiii] Use of Antiretroviral Drugs for Treating Pregnant Women and Preventing HIV Infections in Infants. World Health Organization. April 2012. PDF file.
[xiv] Guidelines on Co-trimoxazole Prophylaxis for HIV-related Infections among Children, Adolescents and Adults. World Health Organization. 2006. PDF file.
[xv] Experiences Integrating Delivery of Maternal and Child Health Services with Childhood Immunization Programs: Systematic Review Update http://jid.oxfordjournals.org/content/205/suppl_1/S6.full.pdf+htm
[xvi] http://jid.oxfordjournals.org/content/205/suppl_1/S20.full.pdf+html
[xvii] N. Rollins et al. Universal HIV testing of infants at immunization Health facilities: an acceptable and feasible approach for early infant diagnosis in high HIV prevalence settings. *AIDS*, 23(14):1851-7, 2009.
[xviii] M.Sinunu, et al. Evaluating the impact of prevention of Mother-to-Child Transmission of HIV (PMTCT) in Malawi through immunization clinic-based surveillance. : 19th International AIDS Conference: Abstract no. TUPE277
[xix] R. Weigel et al. Effect of provider-initiated testing and counselling and integration of ART services on access to HIV diagnosis and treatment for children in Lilongwe, Malawi: a pre- post comparison. *BMC Pediatrics*, 9:80, 2009.
[xx] S. Topp et al. Opt-out provider-initiated HIV testing and counselling in primary care outpatient Health facilities in Zambia. *Bulletin: World Health Organization*, 89:328-335, 2011.
[xxi] Violari et al. Early antiretroviral therapy and mortality among HIV-infected infants. New England Journal of Medicine. Nov 20;359(21):2233-44. 2008.
[xxii] Newell, ML et al. (2004)
[xxiii] National Protocol Guidelines, Integrated Prevention of Mother-to-Child Transmission of HIV/AID in Zambia, 2008 Page 8.
