# Supplementary material for: A Cluster Randomised Trial on the Impact of Integrating Early Infant HIV Diagnosis with the Expanded Programme on Immunization on Immunization and HIV Testing Rates in Rural Health Facilities in Southern Zambia
Source: PLoS One. 2015 Oct 29;10(10):e0141455. doi: 10.1371/journal.pone.0141455 (PMC4626083; doi:10.1371/journal.pone.0141455)
Supplement: S2 Protocol — (DOCX) [file pone.0141455.s004.docx]

**Measuring the impacts of health facility reinforcement and EID and EPI service integration on testing and immunization services in Southern Province, Zambia**

**CONFIDENTIAL**

**EVALUATION PROTOCOL SUBMISSION FOR ETHICAL REVIEW**

**Date Revised: June** 14, 2013

**Principal Investigator:** Paul Wang, Founding Partner, IDinsight – Zambia

**Co-Principal Investigator:** Dr. Godfrey Biemba, Boston University/ZCAHRD

**Co-Investigators:**

- Dr. Max Bweupe, Deputy Director Public Health and Research, Zambia Ministry of Health
- Dr. Pascalina Chanda-Kapata, Principal Surveillance and Research Officer, Zambia Ministry of Health
- Dr. Albert Mwango, National ART Coordinator, Zambia Ministry of Health
- Dr. Penelope Kalesha, Child Health Specialist, Zambia Ministry of Community Development, Mother and Child Health
- Dr. Simon Mutembo, Clinical Care Officer, Provincial Health Office, Southern Province, Zambia Ministry of Health
- Dr. Godfrey Biemba, Country Director, Zambia Centre for Applied Health Research and Development
- Dr. Davidson Hamer, Director of Research and Evaluation, Zambia Centre for Applied Health Research and Development
- Elizabeth McCarthy, Director - Applied Analytics Team, Clinton Health Access Initiative
- Bwalya Kangwa, Program Manager – Demand Driven Evaluations for Decisions, Clinton Health Access Initiative

**Table of Contents**

[Table of Acronyms 3](#_Toc351131234)

[Background and Introduction 4](#_Toc351131235)

[Rationale for Study and Context 5](#_Toc351131236)

[Literature Review 6](#_Toc351131237)

[Study Significance 8](#_Toc351131238)

[Summary of Interventions 8](#_Toc351131239)

[Study Aims 9](#_Toc351131240)

[Specific study objectives 9](#_Toc351131241)

[Study Design and Methods 10](#_Toc351131242)

[Data Sources and Validation 16](#_Toc351131243)

[Study Period 17](#_Toc351131244)

[Outcomes and Expected Results 17](#_Toc351131245)

[Ethical Issues 17](#_Toc351131246)

[Discussion 19](#_Toc351131247)

[Limitations 19](#_Toc351131248)

[Dissemination of Findings 20](#_Toc351131249)

[Budget 20](#_Toc351131250)

[Appendix 1: Information Sheet 22](#_Toc351131251)

[Appendix 2: Informed Consent Form 22](#_Toc351131252)

[Appendix 3: Authorization Letter to Work in Study Sites 25](#_Toc351131253)

[Appendix 4: Study Sample Health Facilities 32](#_Toc351131254)

[Appendix 5: Surveys 32](#_Toc351131255)

[Appendix 6: Investigator CVs 43](#_Toc351131256)

[Appendix 7: References 96](#_Toc351131257)

Table of Acronyms

ANC – Antenatal Care

ART – Antiretroviral Treatment

CHAI – Clinton Health Access Initiative

CHW – Community Health Worker

DBS – Dried Blood Spot

DHO – District Health Office

DMO – District Medical Office

DNA PCR Test – DNA Polymerase Chain Reaction Test for HIV

DPT – Diphtheria, Pertussis (Whooping Cough), and Tetanus Vaccine

EID – Early Infant Diagnosis

EPI – Expanded Programme on Immunization

eMTCT – Eliminating Mother to Child Transmission of HIV

MCH – Mother and Child Health

MoCDMCH – Ministry of Community Development, Mother and Child Health

MoH – Ministry of Health

OPV – Oral Polio Vaccine

PITC – Provider Initiated Testing and Counseling

PMTCT – Preventing Mother to Child Transmission of HIV

PNC – Postnatal Care

SMAG – Safe Motherhood Action Group

U5 – Under-5 Children’s Health Appointments

ZCAHRD – Zambia Center for Applied Health Research and Development

3DE – Demand Driven Evaluations for Decisions InitiativeExecutive Summary
Zambia has made significant progress in expanding HIV testing services and treatment within the mother-child “cascade” which includes identifying and treating HIV-positive mothers, HIV-exposed infants and HIV-positive infants. Early infant HIV diagnosis (EID) services have been implemented in over 800 health facilities nationwide and three DNA PCR laboratories have been established in Ndola and Lusaka. However, despite recent progress, there still exist gaps in the identification of HIV-exposed and HIV-infected infants and linkage to care. Zambia’s Ministry of Health (MoH) guidelines dictate that known HIV-exposed babies should be tested twice for HIV status after birth – once at six weeks and again at six months using a DNA PCR test. However, although an estimated 80,000-90,000 HIV-positive women give birth in Zambia each year, only 45,000 – 48,000 infant DNA PCR tests (out of the recommended 160,000-180,000 tests) are conducted each year.^[[1]](#endnote-1)^^[[2]](#endnote-2)^ Thus, improving EID testing rates represents a critical step in improving Zambia’s performance along the mother-child HIV testing and treatment cascade.

Senior officials from Zambia’s MoH and Ministry of Community Development, Mother and Child Health (MoCDMCH) have requested this evaluation to assess two interventions designed to improve EID testing rates:

1. The **“Simple Intervention”** targets two potential causes of low EID rates: supply stock outs and poor understanding of testing requirements and guidelines. Health facilities receiving the Simple Intervention will benefit from 1) a guaranteed supply of antibody and dried blood spot (DBS) DNA PCR testing materials and 2) a short workshop from district MoCDMCH staff to review and emphasize existing MoH EID guidelines.
2. The **“Comprehensive Intervention”** includes the supply and information components of the Simple Intervention, and also introduces 1) an intentional operational optimization and integration of EID testing into routine six-week immunization visits, and 2) an additional component of opt-out rapid HIV testing for all mothers with previously negative or unknown HIV status in order to identify previously unrecognized HIV-exposed infants.

A cluster randomized evaluation design – randomized at the health facility level – will be utilized to identify the impact of the Simple and Comprehensive Interventions on HIV and immunization indicators. Relevant baseline and outcome data on the number of HIV antibody tests, DNA PCR tests and immunizations will be collected from the facility registers by trained enumerators. Additionally, exit interviews will be conducted for a sub-sample of women in evaluation health facilities. A secondary outcome of the Comprehensive Intervention is that it will provide an estimate of the incidence of HIV among women who previously tested negative for HIV during pregnancy.

This combination of quantitative and qualitative investigations is designed to provide insight for policy makers into the impact of these activities as well as general attitudes of respondents on the interventions.

While this study is designed primarily to inform Zambia’s EID policies and guidelines to improve health and well-being throughout Zambia, it will also contribute to the global knowledge base regarding the integration of HIV services with other popular services and related topics.

Background and Introduction

Although progress has been made across Sub-Saharan Africa in increasing access to “prevention of mother to child transmission of HIV” (PMTCT) services, significant challenges remain. In 2011, an estimated 330,000 infants were born with HIV worldwide.^[[3]](#endnote-3)^ For 21 priority countries in Sub-Saharan Africa, PMTCT service coverage rates were only 61% before pregnancy and 28% during breastfeeding.^[[4]](#endnote-4)^ Similarly, only 28% of HIV exposed infants received an HIV test within the first two months of life.^[[5]](#endnote-5)^ Zambia is no exception, with only 27.4% of HIV-exposed infants tested within the first two months of life.^[[6]](#endnote-6)^

Early identification of HIV-positive infants is critical in improving their chances of survival. Without identification and treatment, it is estimated that over 50% will not survive through their second year.^[[7]](#endnote-7)^ For HIV-positive infants who are identified early and start treatment before the 12-week mark, up to 75% of these deaths can be avoided.^[[8]](#endnote-8)^

Zambia has made strong progress in expanding PMTCT services over the last three years, with 94% of women undergoing HIV testing at Antenatal Care (ANC) in 2010 and 86% of HIV-positive women receiving efficacious antiretroviral for PMTCT in 2011.^[[9]](#endnote-9)^ Unfortunately, these antenatal PMTCT successes have not carried over into post-natal EID rates. Current MoH guidelines stipulate that each HIV-exposed infant should be tested twice using the DNA PCR test, first at 6 weeks after birth and again at 6 months after birth. Because an estimated 80,000-90,000 HIV-positive women give birth in Zambia each year, 160,000-180,000 DNA PCR tests should be processed annually if MoH guidelines are followed. However, in 2011, only 45,000 DNA PCR tests were processed –25% of the national target.^[[10]](#endnote-10)^^[[11]](#endnote-11)^

In Zambia, uptake of routine under-five immunizations is high, with immunization coverage for all vaccines estimated to exceed 80%.^[[12]](#endnote-12)^ Routine immunization, following the recommended World Health Organization (WHO) Expanded Programme on Immunizations (EPI) guidelines, is scheduled at birth, six weeks, ten weeks, fourteen weeks and nine months, with different vaccines administered at each time point. The high coverage of DTP1 vaccinations, estimated at 87% in 2011, suggests that the 6-week visit is particularly well attended by mother-baby pairs. These high immunization rates have prompted Zambian health officials to explore the possibility of utilizing routine immunization as an opportunity to boost Zambia’s EID rates.

Zambia’s MoH and international partners have invested in improving the infrastructure and systems necessary to increase EID testing services in recent years. EID is now available in over 800 health facilities in all 10 provinces. There are currently two DNA PCR labs in Lusaka, one in Ndola, and additional labs scheduled to open in Chipata and Livingstone. The Zambia Centre for Applied Health Research and Development (ZCAHRD), the implementing partner for this evaluation, is also scaling up an SMS-based test results reporting platform that halves the time required to return EID test results to health facilities. Once operational, these additional DNA PCR labs and SMS services will reduce the transport burden for time-sensitive lab results and increase the nation’s overall HIV testing capacity. Given Zambia’s increasing HIV laboratory infrastructure, it is an appropriate moment to explore opportunities to increase the nation’s overall EID testing rates.

Study Objectives and Context

The primary objectives of the study are to assess the impact of:

1. Study interventions on EID testing rates of HIV-exposed infants
2. Study interventions on the number of women identified to be HIV-positive
3. The Comprehensive Intervention on infant immunization uptake

Secondary objective include:

1. Estimating the incidence of HIV among women who previously tested negative for HIV during pregnancy
2. Evaluating the cost-effectiveness of the Simple Intervention versus the Comprehensive Intervention.

The primary goal of this study is to evaluate different measures that might improve EID testing rates and identification of HIV-positive mothers and babies without negatively affecting uptake of immunization services. Identifying such measures has recently become highly relevant due to the Zambian government’s recent announcement to support a strategy of universal HIV testing and implementation of Option B+ nationwide as a strategy to eliminate mother-to-child transmission of HIV.^[[13]](#footnote-1)^ ^[[14]](#endnote-13)^ Critical to the success of Option B+ is the need to make sure that a high percentage of women are tested for HIV and placed on treatment if discovered to be HIV-positive. For this reason, the study intervention is also designed to increase the number of new mothers that are tested for HIV.

MoH officials have identified routine under-five immunizations in health facilities as an opportunity to boost EID activities. The routine immunization schedule – where infants are scheduled to attend health facilities at six weeks, ten weeks, fourteen weeks, and nine months – aligns well with the DBS testing schedule. Ideally, DBS tests should be conducted at six weeks after birth. Then the results should be transported to the laboratory, analyzed, and returned to the health facility three to six weeks later. This time frame allows subsequent immunization visits to be used as times to communicate test results and refer for treatment if necessary.

Finally, WHO guidelines recommend that all HIV-exposed infants should receive cotrimoxazole prophylaxis, an antimicrobial agent, 4-6 weeks after birth, and continue that treatment until HIV infection can be excluded.^[[15]](#endnote-14)^ As part of Zambia’s minimum HIV-exposed infant care and treatment package, providers are trained to initiate HIV-exposed infants on cotrimoxazole prophylaxis from 6 weeks of age onwards. However, Zambia’s MoH 2010 PMTCT report stated that only 40% of HIV-exposed infants were being properly initiated on cotrimoxazole prophylaxis. Possible reasons for this include: poor recording and documentation of the prophylaxis, lack of proper identification of HIV-exposed infants, and frequent stock outs of the drug. Integrating HIV services more closely with routine immunizations represents an additional opportunity to identify many more HIV-exposed infants and initiate them on cotrimoxazole prophylaxis.^2^

Literature Review
Research indicates that integrating universal opt-out mother and infant HIV testing with routine immunizations could be an effective approach to improve mother and infant diagnosis. However, this specific intervention has never been evaluated using an experimental design, and could fill a number of gaps remaining in the global literature. Thus, the proposed evaluation has the potential to make important contributions to both Zambia’s policy landscape and the global knowledge base on HIV testing and health service integration at the point of service delivery.

Below is a discussion on related research on routine HIV testing and health service integration.

*Integrating Health Services with Immunizations*

Wallace et al (2012A) conducted a systematic literature review on the integration of immunization services with other maternal and child health services. They found that integration had a positive impact on the uptake of all newly integrated services, although the coverage rates of non-immunization services did not achieve the same high rate of coverage as immunization. The study identified the use of immunization visits as a possible venue for HIV testing to evaluate the performance of PMTCT services. Important barriers to integration mentioned included adding additional time to the visit length for each mother.^[[16]](#endnote-15)^

Partapuri et al (2012) conducted a literature review on the possibility of integrating additional maternal and child services into immunization outreach campaigns. Interventions included in the review were: ANC, deworming, growth monitoring, bed nets, Integrated Management of Childhood Illness (IMCI), nutrition, and hygiene. No HIV related interventions were included in the review. The reviewers found that integration is most effective when interventions can be feasibly integrated at the outreach level, coordination can be conducted at all program levels, joint training and supervision of health workers and programs is conducted, community based organizations are engaged, and monitoring and evaluation systems provide timely feedback. The researchers focused on the importance in this setting of strong engagement with community health volunteers in promoting the benefits of integration. ^[[17]](#endnote-16)^

*HIV Testing at Time of Immunizations*

Rollins et al. (2009) ran a study at three health facilities in KwaZulu Natal, South Africa, where all mothers who brought infants in for immunizations were also offered HIV testing of infants. Infants were first tested for the presence of HIV antibodies to determine HIV exposure, and if antibodies were present, then they were tested for HIV DNA to determine HIV infection. They found that universal HIV infant testing at immunization health facilities was feasible to identify and refer HIV-infected infants. Of the 646 mothers who brought their infants for immunizations, 90.4% agreed to the HIV testing and 56.8% of those mothers returned for results.^[[18]](#endnote-17)^

Sinunu et al. (2011) ran a study in Malawi to determine the mother-to-child transmission rate in the country ahead of the adoption of Option B+. The research team utilized a random selection process to select a sample of mother-infant pairs attending immunizations to test for HIV-exposure. They found that 9.8% of mother’s tested positive who had never tested before, 6.9% of women who tested negative before pregnancy were now HIV-positive, and 4% of women who tested negative since pregnancy were now HIV-positive. The overall prevalence rate for mothers was 14.4% with 8.4% of positive mothers passing HIV onto their infant.^[[19]](#endnote-18)^

*Provider Initiated Testing and Counseling (PITC)*

A study in Lilongwe, Malawi, used a pre- post comparison to find that implementation of provider-initiated HIV testing and counseling, and integration of ART services in the pediatric ward, was associated with an increase in uptake of testing services and number of patients initiated on ART. The proportion of children and adults initiating ART each quarter increased from 26% to 53%, and 20% to 52%, respectively.^[[20]](#endnote-19)^

In Zambia, a study at primary care outpatient health facilities in Lusaka, Zambia, found that introducing routine PITC significantly increased the uptake and acceptability of HIV testing. After the addition of PITC, the nine health facilities in the sample group tested over twice as many patients as before. Over time, the percentage of individuals who accepted testing rose, indicating that introducing PITC helped to decrease the stigma surrounding HIV testing.^[[21]](#endnote-20)^

*Medical Benefits of Early Identification and Treatments of HIV-Positive Children*A randomized trial conducted by the National Institutes of Health found benefits to early infant initiation of ART, instead of waiting to initiate treatment until disease symptoms are seen. This is especially important since HIV disease progression occurs more rapidly in infants than in adults. In the study, early identification of HIV status and early initiation of antiretroviral therapy reduced early infant mortality by 76%.^^[[22]](#endnote-21)^^

A study of infected and uninfected infants born to HIV-positive mothers found that babies that were identified early and introduced to treatment had significantly better mortality outcomes than those who did not immediately go on treatment. At 1 year of age, 35.2% of infected infants died without treatment and 52.5% had died by 2 years of age without treatment.^[[23]](#endnote-22)^

*Contribution of Evaluation to Literature*

This proposed study will contribute to the literature in several important ways. First, it will assess the extent to which integration of universal HIV testing with routine under-five immunizations, similar to current practice with ANC, can boost HIV testing rates without negatively impacting uptake of immunization. Secondly, it would inform Zambia and other high HIV prevalence countries whether either intervention could be a cost effective approach to identifying new HIV positive infants and mothers. Finally, this study will provide insight into the feasibility of integrating HIV into other public health services.

Study Significance
This evaluation will be the second evaluation launched under the Demand Driven Evaluations for Decisions (3DE) initiative, a three-year partnership between the Clinton Health Access Initiative (CHAI), IDinsight, Zambia’s MoH, and Zambia’s MoCDMCH. By using rigorous impact evaluations in a demand-driven and efficient manner, 3DE seeks to generate reliable impact evidence to catalyze at-scale implementation of cost-effective policies.

The evaluation will help Zambia’s MoH and MoCDMCH determine if the Simple Intervention or Comprehensive Intervention can cost-effectively improve HIV testing rates and the identification of HIV-positive and HIV-exposed mothers and babies without harming under-five immunization uptake. Furthermore, the evaluation will illuminate the operational requirements of these activities, including requirements for funding, staff, training, communication, equipment and logistics. The results of this evaluation will inform the potential scale up of either intervention as well as broad policy decisions on the possibility of integrating HIV services more closely with other existing patient touch points.

Finally, the results will contribute new findings to a limited global evidence base. There are few studies that have measured the impact on immunizations caused by the integration of HIV services despite the fact that several countries are moving towards this approach. Likewise, there are few studies that have examined the benefits of another round of universal HIV testing of infants to identify more HIV+ mothers and HIV-exposed babies.

Description of Interventions

This section describes the two interventions that will be tested in this evaluation, hereafter referred to as the “Simple Intervention” and the “Comprehensive Intervention.”

The **Simple Intervention** targets two potential causes of low EID rates: (1) supply stock outs and (2) poor understanding of existing MoH testing requirements and guidelines. This intervention will have two components:

1. **Supply Reinforcement** – The research team will work with facility level staff to ensure that orders for necessary HIV testing supplies are placed on time and in sufficient quantity. In the event of a generalized stock out of HIV testing supplies at the province or district level, the evaluation team will provide facilities with an additional outside supply to allow them to continue testing operations.
2. **Guidelines Reinforcement** – The research team will arrange facility visits by district health office officials to meet with relevant health staff, remind them of exiting MoH HIV testing guidelines, and remind them that improving EID testing is a Ministry priority.

The **Comprehensive Intervention** integrates universal, opt-out HIV screening and Early Infant Diagnosis of HIV (EID) services with Expanded Program on Immunization (EPI) Services in Zambia. This intervention will include:

1. Simultaneous provision of services to a mother-infant pair by health care workers without interruption between services
2. Opt out testing of mothers with unknown or prior negative status

In addition, the Comprehensive Intervention will—in an identical manner to the Simple Intervention—reinforce the supply of HIV testing equipment at facility level and reinforce guidelines among health professionals.

Study Aims

The aims of the study are described below:

1. Estimate the impact of both the Simple Intervention and Comprehensive Intervention on the following HIV-related outcomes:
   1. Percentage of mothers with unknown or previously negative HIV status tested for HIV-infection (antibody test)^[[24]](#footnote-2)^
   2. Percentage of HIV-positive women newly identified^[[25]](#footnote-3)^
   3. Percentage of HIV-exposed infants tested for HIV status (DNA PCR test)^[[26]](#footnote-4)^
2. Estimate the impacts of the Comprehensive Intervention on the following immunization-related outcomes:
   1. Percentage^[[27]](#footnote-5)^ of infants receiving first-visit immunizations
   2. Percentage of infants receiving second-visit immunizations^[[28]](#footnote-6)^
3. Assess the cost, staffing and testing requirements of the Simple Intervention and Comprehensive Intervention.

Specific Study Objectives

| **Specific research question** | **Key activities** |
| --- | --- |
| What is the impact of the Simple and Comprehensive Interventions on HIV testing parameters? | Compare program and comparison sites on the following measures:   - Total number of mother-infant pairs tested for HIV-infection (antibody test) - Total number of infants tested for HIV status (DNA PCR test) - Total number of HIV-positive women identified |
| What is the impact of the intervention on the percentage of infants receiving first-visit and second-visit EPI immunizations? | Compare program and comparison sites on the percentage of infants receiving DTP1, OPV1, DPT2 and OPV2 |
| What is the cost of implementing the Simple and Comprehensive Interventions?  What staff capacity is required at the facility level for the Simple and Comprehensive Interventions?  What HIV testing capacity is required to scale the Simple and Comprehensive Interventions across Zambia? | Build a costing model to examine the cost-benefit of implementing both interventions relative to improved morbidity and mortality  Build a model to assess the staff and HIV test capacity required to implement the intervention nation-wide |

Study Design and Procedures

Design

This is a cluster randomized controlled trial; each cluster being a health facility. The clusters will be randomly sorted into the following three arms:

1. Simple Intervention: Reinforcement of supplies and supply chain management for HIV testing; reinforcement of HIV testing guidelines.
2. Comprehensive Intervention: All components of the simple intervention plus;

- Universal opt-out HIV screening for all previously negative or status unknown mothers attending their infant’s first immunization
- Operational integration of Early Infant Diagnosis (EID) and Expanded Program of Immunization (EPI)
- Training and orientation of health facility staff to deliver intervention messages to mothers of children under five years

1. Control: Usual standard of care.

We will then evaluate the two interventions in comparison to the control group, as well compare the two intervention groups to each other, in terms of specific outcomes (See the power calculations section for specific outcome variables) by a review of clinic records collected over the period of the study.

We will supplement and triangulate the data obtained from the clinic records through four primary data collection tools:

- Focus Group #1: Mothers who have recently attended Under-5 clinics
- Focus Group #2: Health facility workers in sampled health facilities Interview guide for exit interviews for mothers done at health facilities
- Interview guide for data verification for mothers done in local communities

Random sampling will be done at the health facility level to separate health facilities in the sample frame into 4 groups – 1) Comparison 2) Simple Intervention 3) Comprehensive Intervention and 4) Not in the study sample. Sampling will be conducted using STATA IC Version 12 with a pre-set seeds to allow for post-randomization verification of selection methods. There are 77 clinics in the sample frame spread across three districts in Southern Province in Zambia. The sample will be stratified by urban/rural divide and by district. Please see the table below for information on the stratification within the sample frame and sample respectively.

**Table 1: Sample Stratification**

|  | Total Facilities | Total Urban | Total Rural | Sample Total | Sample Urban | Sample Rural |
| --- | --- | --- | --- | --- | --- | --- |
| Choma | 37 | 6 | 31 | 30 | 6 | 24 |
| Livingstone | 15 | 11 | 4 | 9 | 9 | 0 |
| Monze | 25 | 3 | 22 | 21 | 3 | 18 |
| Total | 77 | 20 | 57 | 60 | 18 | 42 |

Sampling of clinics will not be weighted by population; therefore, individuals who live in facilities with smaller catchment areas have slightly higher probability of being included in the study than those that live in the catchment area of larger facilities. Within each strata, each facility has an equal chance of being allocated into each of the three evaluation arms. Randomization will not occur on site but will be conducted before the interventions begin. Please note, at the time of submission to IRB, the final sample has not been run in STATA.

**Fig.1: Schematic diagram of the study design**

**Simple Intervention**

- Testing kits supply reinforcement

- Guidelines reinforcement

**Intervention activities**

**Control Group**

-Usual standard of care

**Comprehensive Intervention**

-Supply reinforcement

-Guidelines reinforcement

-Clinic flow optimization

-Changes to testing algorithm

- Review of health facility client records, supplemented by review laboratory data for verification
- Village surveys
- Focus Group Discussions with mothers who have recently attended Under-5 clinics
- Focus Group Discussions with health facility workers participating in the evaluation
- Exit Interviews with mothers of children under five years done at health facilities
- Data Verification interviews with mothers done in local communities

**Evaluation activities**

**Procedures**

There are two sets of activities for carrying out this study. First we describe the interventions that will be put in place (intervention activities). Secondly, we will then describe how we will evaluate the effect of those interventions on various outcome measures (evaluation activities).

Intervention Activities

The two intervention arms (third is control) will receive the following:

*Simple Intervention implementation*

1) Supply Reinforcement – The research team will procure separate supplies of HIV test kits to cover all expected additional tests that will be done as a result of this evaluation. The research team will meet with facility staff, provide their contact information, and instruct them to call the researchers if there is a stockout of testing supplies that cannot be filled by the district health office.

2) Guidelines Reinforcement – The research team will arrange meetings between district health staff and selected health facilities that reminds current clinic staff of existing guidelines for testing at 6 weeks and reiterates that following these guidelines is a ministry priority. This meeting will take place at the district health office and will be run by the district medical officer and his/her staff. The presentation will include a review of all relevant guidelines as well as emphasis that improvement in these areas is a national, provincial, and district priority.

*Comprehensive Intervention Implementation*

All components of the Simple Intervention will also be implemented in the Comprehensive Intervention health facilities with the following additions:

1) Clinic Flow Optimization – The research team will conduct an assessment of existing clinic flow in all comprehensive intervention facilities. After site selection, the research team will meet with each district medical officer and district mother and child health coordinator to review plans for changing clinic flows in each selected facility. Next, the research team will organize a meeting with each facility in-charge to discuss the intervention and receive feedback on plans. The research team will then conduct a 1 day workshop with each facility on methods to improve clinic flow at under-5 clinics. This approach will be piloted for 1-2 weeks under supervision of research team staff or until the research staff is confident that changes have been properly implemented at each health facility.

2) Changes to Testing Algorithm – The research team will conduct a short training on changes to the HIV testing Algorithm that has been approved by Zambia’s Ministry of Health for this evaluation. This training will be a part of the clinic flow optimization workshop mentioned above. Currently, only mothers who have not been tested in the last 3 months are supposed to be tested at under-5 clinics, although adherence to this guideline is poor. Under the study design, all mother-infant pairs will be tested for HIV at their first under-5 visit (at 6 weeks) unless they have previously tested HIV-positive.

Evaluation Activities:

We shall randomly select 60 health facilities from a total of 77 in Southern province of Zambia (see sample size section). These health facilities will be randomly allocated into three study arms. All facilities will receive basic training on proper patient documentation across the different registers used to record EID, EPI, and under-five year old children visit information.

*1.0 Review of health facility client records, supplemented by review laboratory data for verification*

A field team of no more than 6 field officers will visit the 60 health facilities once every 2 weeks. Administrative data, meaning aggregated patient data, will be collected from health facility tally sheets and registers and inputted in aggregate form into mobile phones using google.org’s Open Data Kit application. Different data points will be drawn from different registers:

- Immunization data will be collected from all health facility tally sheets
- HIV Rapid Test Data will be collected from Tally Sheets and the BU Integrated PMTCT Register
- HIV Dried Blood Spot (DBS) testing data will be collected from the DBS tracking register and confirmed by data in laboratory data base at the University Teaching Hospital in Lusaka.

*2.0 Focus Group Discussions (FGDs) with mothers who have recently attended Under-5 clinics*

We will randomly select 15 health facilities from the list of the sampled 60 facilities; five from each of the three arms of the study. From the catchment population around each of these facilities, we will conduct FGDs with mothers who have attended under-5 clinic at that facility in the last 6 months. Similar to the data verification study, we will randomly sample villages and work with village leaders to select the participants. These focus groups are designed to provide complimentary qualitative evidence on perceptions of HIV testing, particularly their effect on immunizations at under-5 clinics, which could inform any potential scale-up of the interventions studied. Each focus group will have 6-10 participants. Focus group scripts are attached in Annex 1.

*3.0 Focus Group Discussions with health facility workers participating in the evaluation*

Fifteen health facilities will be randomly selected from the list of 60 sampled facilities to hold focus group discussions with the health facility staff. These focus groups will last no more than an hour and will be conducted in English. These focus groups are designed to provide complimentary qualitative evidence on perceptions of health facility workers on the implementation of the intervention, particularly on issues of testing kit supply and staff time, which could inform any potential scale-up of the interventions studied. Each focus group will have 6-10 participants. Focus group scripts are attached in Annex 2.

*4.0 Exit Interviews with mothers of children under five years done at health facilities*

The research team will conduct exit interviews for mothers at under-5 visits three times per facility during the data collection period. Mothers will be randomly asked to participate in interviews as they exit facilities such that 5% of the study population of mothers will be interviewed. Study enumerators will interview every second mother for small facilities and every third mother for large health facilities. Interviews will take no more than 30 minutes. An interview guide has been prepared as Annex 3.

*5.0 Data Verification interviews with mothers done in local communities*

The research team will conduct data verification interviews for mothers in communities within the catchment areas of sample health facilities. The research team will collect a list of villages from each sampled health facility and randomly select 5% of the villages to conduct data verification interviews in. The research team will work with the facility staff to be put in contact with local village headmen and work with them to arrange a community meeting for all women who have given birth and attended under-5 clinic in the last 6-months. The research team will collect information from the mother/infant under-5 card and record it using mobile data collection. Separate members of the research team will then be assigned to collect the same data from health facility registers. Finally, the research associate will compare the two sets of records. Interview will take no more than 30 minutes. An interview guide has been prepared as Annex 4.

*Outcomes and Expected Results*

The primary outcomes of the research questions are:

1. *HIV service delivery rates:* the difference in the following indicators will be compared between the comparison and program groups
   1. Percentage of Mother-infant pairs tested for HIV exposure (antibody test)
   2. Percentage of infants tested for HIV status (DNA PCR test)
   3. Percentage of HIV-positive women identified
2. *Immunization rates:* the difference in the percentage of infants receiving first-visit and second-visit immunizations will be compared between the Comprehensive Intervention groups and the other two evaluation groups.
3. Operational considerations
   1. Assess the cost, staffing and testing requirements of the Simple and Comprehensive Interventions per additional HIV-positive infant and mother identified
   2. Project at-scale cost of the interventions that show a positive impact

*Study Period*

This study will take place between June 2013 and February 2014. All data collected for this analysis will come from this study period. Relevant MoH and MoCDMCH officials will be updated on a monthly basis by the research team. A Gantt Chart is attached in Annex 5.

**Sample Size and Justification**

*Sample frame*

This study will take place in the Southern Province of Zambia in health facilities supported by ZCAHRD that are providing PMTCT and EID services at facility level. The sample frame comprises of 77 health facilities in Choma, Livingstone, and Monze districts. These districts were selected out the 10 districts in Southern Province based on leadership, geographic dispersion, urban / rural characteristics, current HIV prevalence rates, and absence of conflicting research projects. Hospitals and facilities without adequate skilled staff were excluded from the sample frame. The selection of Southern Province was made in conjunction with MoH officials who communicated that lessons learned from an evaluation in Southern Province could be generalized to other provinces in Zambia for purposes of scale up.

*Sample:* We will enroll a total of 11,910 participants into the study.

*Justification*

It has been determined that a sample of 60 facilities (20 facilities for each evaluation group) – selected out of the sample frame of 77 facilities – is required for the study.  The sample will be stratified by an urban/rural divide and district, with a third of the sites randomly chosen as comparison facilities, a third randomly chosen as Simple Intervention facilities, and the final third randomly chosen as Comprehensive Intervention facilities. Thus, the unit of randomization will be the health facility and all infants attending the given health facility for immunizations during the period of the evaluation will be the cluster.  Random assignment and post randomization checks will ensure that on average, observable and unobservable characteristics of the program and comparison groups are statistically similar. Observable characteristics that will be checked for balance across evaluation arms include baseline HIV testing rates, baseline immunization rates, number of ART sites, and catchment population sizes. In doing so, any difference in outcomes between program and comparison group can be causally attributed to the intervention. In total, we will have 60 clinics in the study, each of which see on average around 260 infants for their first immunization each year. To calculate the number of infants we expect to see, in our study, we divide 260 in half (we will only collect data for 6 months) and then we will take 75% of this remaining number (to account for around 25% of immunizations which will be done on outreach). This yields around 98 babies per facility multiplied by 60 facilities for a total of 5,880 infants. If we count mothers and infants as separate entities, we get a total of 11,760 participants. Up to 5% of the mothers/care givers in the study sample will be selected for data verification and a separate 5% of mothers will be selected for exit interviews, which is 294 mothers for each group. Additionally, up to 30 focus groups will be held, 15 for recent mothers and 15 with facility staff, with each focus group including no more than 10 individuals. The 150 mothers participating in the focus group are already included in the 11,760 participants. The 150 health facility staff participating in the focus groups are all new participants and must be added to the 11,760 participant number for a total of 11,910.

This study is designed to measure the impact of the Simple Intervention and Comprehensive Intervention on the number of women and infants tested and identified as HIV-positive as well as the percentage uptake of certain immunizations. Statistical power calculations are based on the immunization uptake outcome variable, as this is the indicator with the smallest effect size required to be policy-relevant. The study will test the following null hypotheses:

- - 1. Infants are not less likely to attend immunization clinics for DPT1, OPV1, DPT2 and OPV2 immunizations in Comprehensive Intervention health facilities versus the combination of the Simple Intervention and Comparison health facilities
    2. Rapid HIV antibody tests are not more likely to be administered in:
       1. Simple Intervention facilities versus Comparison facilities
       2. Comprehensive Intervention facilities versus Simple Intervention facilities
    3. DNA PCR tests are not more likely to be administered in:
       1. Simple Intervention facilities versus Comparison facilities
       2. Comprehensive Intervention facilities versus Simple Intervention facilities
    4. Mothers are not more likely to be identified as HIV-positive in:
       1. Simple Intervention facilities versus Comparison facilities
       2. Comprehensive Intervention facilities versus Simple Intervention facilities

Below are the power calculation for each of the outcomes we intend to measure:

*Effect of Comprehensive Intervention on immunization rates*

- **Level of outcome:** DPT 1 immunization
- **Sample:**
  - 20 Comprehensive Intervention facilities compared to 40 Simple Intervention and Comparison facilities
  - 98 new infants per facility
- **Detectable percentage point decrease in immunization uptake:** 10%
- **95% plausibility interval:** Expected probability that an infant receives DPT1 immunization is 87% with bounds around this estimate from 55 – 95%
- **Significance level:** 5%
- **Power:** 86%^[[29]](#footnote-7)^

*Effect of Simple Intervention on rapid HIV antibody testing rates (Simple versus Comparison facilities)*

- **Level of outcome:** Rapid HIV antibody tests administered
- **Sample:**
  - 20 Simple Intervention facilities compared to 20 Comparison facilities
  - 98 new infants per facility
- **Detectable percentage point increase in antibody tests:** 5%
- **95% plausibility interval:** Expected probability that a mother/infant pair receives a rapid HIV antibody test is 5% with bounds around this estimate from 1 - 10%
- **Significance level:** 5%
- **Power:** 87%

*Effect of Comprehensive Intervention on rapid HIV antibody testing rates (Comprehensive versus Simple facilities)*

- **Level of outcome:** Rapid HIV antibody tests administered
- **Sample:**
  - 20 Comprehensive Intervention facilities compared to 20 Simple Intervention facilities.
  - 98 new infants per facility
- **Detectable percentage point increase in antibody tests:** 20%
- **95% plausibility interval:** Expected probability that a mother/infant pair receives a rapid HIV antibody test is 15% with bounds around this estimate from 1 - 30%^[[30]](#footnote-8)^
- **Significance level:** 5%
- **Power:** 93%

*Effect of Simple Intervention on DBS PCR testing rates (Simple versus Comparison facilities)*

- **Level of outcome:** DBS PCR tests administered
- **Sample:**
  - 20 Simple Intervention facilities compared to 20 Comparison facilities
  - 98 new infants per facility
- **Detectable percentage point increase in DBS PCR tests:** 4%
- **95% plausibility interval:** Expected probability that an infant pair receives a DBS PCR test is 5%^[[31]](#footnote-9)^ with bounds around this estimate from 1 - 10%
- **Significance level:** 5%
- **Power:** 75%^[[32]](#footnote-10)^

*Effect of Comprehensive Intervention on DBS PCR test rates (Comprehensive versus Simple facilities)*

- **Level of outcome:** DBS PCR tests administered
- **Sample:**
  - 20 Comprehensive Intervention facilities compared to 20 Simple Intervention facilities
  - 98 new infants per facility
- **Detectable percentage point increase in DBS PCR tests:** 4%
- **95% plausibility interval:** Expected probability that a mother/infant pair receives a DBS PCR test is 9% with bounds around this estimate from 5 - 14%^[[33]](#footnote-11)^
- **Significance level:** 5%
- **Power:** 82%

*Effect of Simple Intervention on percentage of new mothers identified as HIV-positive (Simple versus Comparison facilities)*

- **Level of outcome:** HIV-positive mothers identified
- **Sample:**
  - 20 Simple Intervention facilities compared to 20 Comparison facilities.
  - 98 mother / infants pairs per facility
- **Detectable percentage point increase in HIV-positive mothers identified:** 0.8%
- **95% plausibility interval:** Expected probability that a mother is identified as HIV-positive is 0.2% with bounds around this estimate from 0.1 – 1.0%
- **Significance level:** 5%
- **Power:** 76%

*Effect of Comprehensive Intervention on percentage of new mothers identified as HIV-positive (Comprehensive versus Simple facilities)*

- **Level of outcome:** HIV-positive mothers identified
- **Sample:**
  - 20 Comprehensive Intervention facilities compared to 20 Simple Intervention facilities
  - 98 mother/infant pairs per facility
- **Detectable percentage point increase in HIV-positive mothers identified:** 1.5%
- **95% plausibility interval:** Expected probability that a mother is identified as HIV-positive is 1.0% with bounds around this estimate from 0.5% – 2.0%
- **Significance level:** 5%
- **Power:** 87%

These power calculations represent lower bounds, as covariate analysis will further increase the actual power of the evaluation. We can assume that because all research questions are sufficiently powered to detect effects between either the comprehensive and simple facilities or the simple facilities and the comparison facilities, all research questions are sufficiently powered to evaluate effects between the comprehensive and comparison clinics in the case where the simple clinics show no effect.

**Data Analysis**

Impacts of the Simple and Comprehensive Interventions on EID testing rates, HIV-positive mothers and infants identified, and immunization data will be estimated by fitting multilevel linear probability and logit[[1]](https://inspir.bu.edu/Study_App.jsp?FORM_MODE=EDIT&tab=section&s=1371040947028#_ftn1)  regressions that compare outcomes between program and comparison facilities while controlling for important covariates, such as historical EID and EPI rates, district, catchment population, facility staff, geography and patient characteristics. Data analysis will be conducted using Stata (version 12). The at-scale costs of both inventions will be modeled using external inputs as well as cost estimates from the evaluation intervention.  Moreover, operational best practices of this intervention will be assessed to determine the feasibility of scaling up either set of intervention nationwide.The primary analysis will be the logit regression.  These analyses relate to the primary study purpose as it will measure the change in EPI and EID uptake in a standard way while controlling for important covariates.  The qualitative focus groups will be used to examine the causal mechanisms of any impacts on outcome indicators but will not be used in the primary analysis.

**Technical study risks and potential biases**

The discussion below describes the approach towards potential research design effects that could pose threats to the integrity of the results of this evaluation.

Important steps will be taken to minimize the risk of Hawthorne effects, the process by which a subject of a study changes their behavior due to the knowledge that they are being surveyed and measured. For this evaluation, this is principally a problem for the EID and HIV testing outcomes of interest. Health facility staff may put extra effort into HIV testing in response to this project in addition to the intervention. To reduce this risk, the research team will work through Ministry of Health channels and District health officials as much as possible to more closely mimic typical managerial attention. This will better approximate Ministry oversight which will continue after the evaluation as compared to a pure research project. Analysis will also be done to see how consistent testing rates are across the duration of the experiment. The outcomes relevant to immunization uptake should not be affected by Hawthorne effects as they center on patient, rather than facility staff, behavior.

The study analysis will account for the risk of spillovers affecting the results of immunization uptake outcome variable between program and comparison facilities. Only one-third of facilities in the evaluation will be Comprehensive Intervention facilities which serve as the program group for the immunization uptake variable whereas the remaining two-thirds of the facilities will serve as a comparison group for this variable. There is a risk that, if the communication for the EID/EPI integration intervention is not properly targeted, mothers in comparison catchment areas may also receive the message. To minimize this risk, all messages will be highly associated to specific facilities via ANC, SMAGs, in-facility delivery, and PNC touch points, which are all facility-specific channels. However, the potential for word of mouth spread of information and communication spillovers remains. Therefore, the research team will analyze the immunization rates in the comparison sites against historical immunization data for these sites for significant changes over time. Finally, exit interviews will be conducted with comparison and program facility officials to check for observed changes in immunization attendance or rumors they may have heard from community members during the evaluation.

An additional spillover risk exists whereby mothers that ordinarily attend Comprehensive Intervention health facilities switch to other health facilities as a result of the Comprehensive Intervention. The study may collect immunization data from a subset of patients from health facilities neighboring Comprehensive Intervention facilities to estimate the magnitude of such potential shifts. Study sampling that maximizes the distance between study facilities will reduce the likelihood that comparison facility immunization rates are artificially increased due to new patients. Comprehensive Intervention facilities may still experience artificially low immunization rates due to transferring patients. Because estimating the proportion of patients that switch facilities that would remain at their typical catchment facility if the Comprehensive Intervention were universally implemented at every health facility would be extremely difficult, it is most appropriate to consider low immunization rates due to transferring patients as an impact of the Comprehensive Intervention although this could overestimate the effect the Comprehensive Intervention would have in a scaled-up environment.

There is a low risk of John Henry effect, the process in an evaluation by which the comparison group realizes that it is a comparison group and changes its behavior in response. Patients are unlikely to realize they are in comparison catchment areas and change their behavior in any way other than outlined in the spillover section. There exists a risk that comparison facilities could take steps to improve EID services that they would not have done in the absence of the evaluation. The research team will attempt to measure this by comparing EID testing rates from before the intervention with testing rates during the evaluation.

There is a low risk of any survey effects for this evaluation. No surveys with durations of over 30 minutes at a time are expected. Because the main outcomes are directly observed, not asked of the respondents, any survey effects are likely to be minimal.

*Site Attrition*

All sites included in the randomization will have appropriate infrastructure and staffing levels to participate in the study. Thus, it is unlikely that facilities will drop out from the study due to inability to implement the intervention. Because unexpected changes to staffing levels may still lead to attrition, additional facilities have been included in the sample size calculations to ensure adequate statistical power in the event of such occurrence.

*Patient attrition*

Patient-level attrition could manifest itself due to incorrect facility register recordings. This will be minimized through training and regular monitoring of facility health staff on proper register data collection. Respondent attrition is possible for those who do not consent to exit interviews. However, these activities are not central to the core analysis.

Bounds analysis will be used to account for any attrition in the ultimate analysis.

*Impact heterogeneity*

Facility registers provide important baseline data on HIV testing, EID, and EPI services and other patient characteristics. Additional baseline information on facility staff, equipment and buildings will be collected. This information will allow the study team to calculate whether the intervention had a differential impact across important variables.

Ethical Issues

We will seek approval from the ERES Converge Ethics Review Board in Lusaka, Zambia and the Boston University IRB. Study staff will comply with the relevant proposal submission policies of ERES Converge in order to gain full ethical approval to conduct the proposed study. After ethical clearance, the proposal will also be submitted to MoH for final permission to conduct the study.

*Risks to participants*

The proposed evaluation is likely to expose individuals to a minimal level of risk. This section will split risks considered into three sections: risks involving patient attendance, risks involving patient’s health facility experience, and risks involving the management of personal data.

Risks involving patient attendance to under-five health facilities are restricted to the Comprehensive Intervention evaluation group. This evaluation will be measuring whether fewer mothers will attend immunization services due to stigma issues surrounding HIV-testing or inconvenience caused by longer appointment times. This represents one of the primary outcomes measured in the study and thus will be monitored very closely. To minimize any potential adverse effects to uptake of routine immunizations, the Comprehensive Intervention will be discontinued if at any time after two months of data collection Comprehensive Intervention facilities are confirmed to have an immunization rate more than 20 percentage points lower than comparison and Simple Intervention Facilities.^[[34]](#footnote-12)^

For Comprehensive Intervention facilities, the patient experience for mothers will change as a result of the administration of the rapid HIV antibody test for mothers not already confirmed to be HIV-positive. This change in testing procedure will be communicated to mothers during the group counseling session so they are fully informed if they would like to opt-out from the HIV testing. Health facility staff will conduct standard counseling protocols if a mother does allow the rapid HIV antibody test to be conducted. The counseling protocol will be done in private, and contain information about the implications of the test and further options. In this way, mothers will be fully informed throughout of the HIV testing procedures and possibilities during the facility visit.

Risks involving data management and patient privacy apply to all study facilities. All test strips and dried blood samples will only be identified with an identification number, ensuring the privacy of all patients. All Ministry of Health HIV testing guidelines will be complied with, and all data will be collected by trained enumerators using paper surveys or electronic devices. Hard copy surveys will be kept in a locked storage room accessible only by study staff. All electronic data will be split into two separate files. Names and all other identifying information will be in one file and remaining data with unique individual identifications numbers will be in the second file. These files will be stored on two locked computers with password protected hard drives to ensure the confidentiality of the data. All data collected via electronic device by the enumerators will be transferred as soon as logistics permit to the research manager’s hard drive and subsequently erased from the enumerator’s digital device. Only 3DE staff conducting the study will have access to identifiable data. The Principal Investigator, Paul Wang, and Research manager, Ben Brockman, will use STATA (version 12) to analyze the data. Primary data analysis will include quality control checks, such as checks for missing data and data entry checks. Data analysis will take place in Zambia.

*Potential benefits of the proposed evaluation*

The evaluation will directly benefit mother-infant pairs who are newly identified as HIV-positive or HIV-exposed during the study period and referred for treatment. If the interventions evaluated are found to be cost-effective, at-scale implementation will increase the number of mothers and infants identified as HIV-positive and the number of mothers and infants referred for treatment. This would be expected to have a positive impact on infant mortality and life expectancy. An increased identification of HIV-positive mothers and infant exposure risk will allow for more women to be put on lifesaving antiretroviral treatment and will contribute to the elimination of mother-to-child transmission of HIV. Selection of health facilities for the program and comparison groups will be done in an equitable, randomized manner. An additional likely outcome is that HIV-related stigmas could be reduced since HIV-positive mothers will no longer be specifically selected for extra activities.

*Informed Consent*

Informed consent will be sought from all study participants before any survey is implemented for study purposes. For any such activity, health facility and study staff will read aloud to participants the attached informed consent statement, which is in English and has been translated into the relevant local languages. The consent statement introduces the broad concept being studied, assures that confidentiality will be maintained, and makes clear that participants have a choice about whether to participate and that they may withdraw at any time. Informed consent will not be sought to copy routinely collected patient data recorded on health facility registers or for additional testing during under-five facility visits. In line with the 2008 Zambia Ministry of Health Guidelines on PMTCT, women who are offered opt-out HIV testing do not need to sign an informed consent form; they are only required to be fully informed of the test.^[[35]](#endnote-23)^

Discussion

Zambia’s Ministry of Health (MoH) leadership has expressed strong interest in increasing EID testing rates and in evaluating the integration of HIV services more closely with U5 services. As previously noted, several countries in Sub-Saharan Africa have formally or informally moved to integrate EID testing in under five health facility visits. Additionally, similar approaches have been the subject of research projects in Tanzania and South Africa; however, neither study was randomized to rigorously evaluate the effect on immunization rates or other important outcomes. This evaluation will fill a significant gap in knowledge in Zambia and in the broader literature on an important topic.

This randomized controlled trial will assess the impact of the Simple Intervention and Comprehensive Intervention on HIV testing rates, HIV-positive identification rates, and immunization uptake rates. The evaluation will provide MoH and MoCDMCH leadership with rigorous evidence to decide whether either set of interventions is worth scaling up and will provide additional policy-relevant insights in several related fields: 1) the feasibility of more closely integrating HIV services with other health services, 2) best practices and resources required to integrate HIV services with other health services and 3) the current efficacy of the PMTCT cascade in a high-prevalence rate of the country. The research questions and design of this study have been developed through consultation with key stakeholders in MoCDMCH, MoH, CHAI, IDinsight, and BU/ZCAHRD to ensure the highest level of rigor and policy relevance. All relevant partners will be informed of study results.

Limitations

It is unlikely that this study’s population will be perfectly statistically representative of the potential scale-up population. This evaluation focuses on rural, peri-urban, and urban areas of Choma, Livingstone, and Monze districts in the Southern Province of Zambia. Statistical power calculations have been conducted to determine effect sizes across the overall sample. Therefore, the research team may only be able to draw indicative results on differences in some of the outcomes variables in urban areas as compared to rural areas. The study will be conducted in medium to high HIV prevalence areas that may differ from low prevalence areas.

The study will only test one approach to reinforcing testing guidelines and one approach to integrating services more closely. It is possible that other approaches to these broad objectives could have different impacts on the outcome variables of interest.

This study may estimate spillover effects due to patients transferring from Comprehensive Intervention to comparison facilities but will not attempt to estimate the proportion of transferred patients that would have remained at their original facility if the Comprehensive Intervention were universally implemented in all facilities. As a result, impacts on immunization rates are likely to be overestimated, and impacts on HIV-related indicators likely to be underestimated. Both of these biases are in the conservative direction. This is deemed acceptable given the policy-focus of this evaluation and that any study conclusions should account for the worst possible policy scenario – which the approach to spillovers should enable.

Finally, due to logistical considerations, the study does not attempt to integrate EID services with outreach EPI services, where health staff members travel to communities to administer routine under-five immunizations. Likewise, this will shade the impacts on immunization rates and HIV-related indicators conservatively, which is ideal to draw policy implications from the study.

Dissemination of Findings

The outcomes and analysis, along with a final recommendation, will be compiled into a study report and policy brief for the MoH and MoCDMCH. If this intervention is scaled up across the country, CHAI and IDinsight will continue to work with the partners to monitor the project’s status. Any findings from this longer-term monitoring period will be added to the policy brief. Contingent on MoH and MoCDMCH approval, findings will be shared with the Zambian and global health community to learn how EID testing rates can be increased and how HIV services can be better integrated with immunization services.

Budget

**Items**

**Amount**

**Field Staff & transport**

Field Staff salary & per diem

278,000

Transport

180,000

**Field Staff & transport subtotal**

**458,000**

**Intervention**

HIV testing backup supply

46,000

Training / engaging health facilities

88,000

**Intervention subtotal**

**134,000**

**Research**

Field staff training

10,000

ERES ethics review

2,625

Data collection instruments

9,500

**Research subtotal**

**22,125**

**GRAND TOTAL (KR)**

**614,125**

**Evaluation Budget (KR)**

References

1. Interview Mr. Clement Phiri, Laboratory Services, Zambian Ministry of Health. November 29^th^, 2012. [↑](#endnote-ref-1)
2. UNGASS Zambia Country Report, Monitoring the Declaration of Commitment on HIV and AIDS and the Universal Access, Biennial Report. Submitted March 31st, 2012 [↑](#endnote-ref-2)
3. GSG Mid-Term Review Meeting Report, Early Infant Diagnosis IATT Laboratory & Child Survival Working Group, EMTCT-IATT, UNICEF. December 6-7, 2012. [↑](#endnote-ref-3)
4. Together we will end AIDS. UNAIDS. 2012. [↑](#endnote-ref-4)
5. UNAIDS Progress Report Summary. 2011. [EID coverage rates at the end of 2011 were not reported in the more recent UNAID Progress Report, 2012] [↑](#endnote-ref-5)
6. UNGASS Zambia Country Report [↑](#endnote-ref-6)
7. Newell, ML et al. Mortality of infected and unifected infants born to HIV-infected mothers in Africa: a pooled analysis. Lancet. Oct 2-8;264(9441):1236-43. 2004. [↑](#endnote-ref-7)
8. UNAIDS 2008 Report on the global AIDS epidemic. [↑](#endnote-ref-8)
9. Countdown to Zero, Elimination of New HIV Infections Among Children by 2015 and Keeping Their Mothers Alive, Zambia. UNICEF. < http://www.unicef.org/aids/files/hiv_pmtctfactsheetZambia.pdf> [↑](#endnote-ref-9)
10. Interview, Clement Phiri, Laboratory Services, MoH November 29^th^, 2012 [↑](#endnote-ref-10)
11. Interview, Clement Phiri. [↑](#endnote-ref-11)
12. Zambia: WHO and UNICEF estimates of immunization coverage: 2011 revision. PDF file. [↑](#endnote-ref-12)
13. Option B+ is a single, universal ART regimen given to all HIV-infected pregnant or breastfeeding women for life, in hopes of simplifying service delivery and reducing mother-to-child transmission of HIV in the current and future pregnancies. [↑](#footnote-ref-1)
14. Use of Antiretroviral Drugs for Treating Pregnant Women and Preventing HIV Infections in Infants. World Health Organization. April 2012. PDF file. [↑](#endnote-ref-13)
15. Guidelines on Co-trimoxazole Prophylaxis for HIV-related Infections among Children, Adolescents and Adults. World Health Organization. 2006. PDF file. [↑](#endnote-ref-14)
16. Experiences Integrating Delivery of Maternal and Child Health Services with Childhood Immunization Programs: Systematic Review Update http://jid.oxfordjournals.org/content/205/suppl_1/S6.full.pdf+htm [↑](#endnote-ref-15)
17. http://jid.oxfordjournals.org/content/205/suppl_1/S20.full.pdf+html [↑](#endnote-ref-16)
18. N. Rollins et al. Universal HIV testing of infants at immunization Health facilities: an acceptable and feasible approach for early infant diagnosis in high HIV prevalence settings. *AIDS*, 23(14):1851-7, 2009. [↑](#endnote-ref-17)
19. M.Sinunu, et al. Evaluating the impact of prevention of Mother-to-Child Transmission of HIV (PMTCT) in Malawi through immunization clinic-based surveillance. : 19th International AIDS Conference: Abstract no. TUPE277 [↑](#endnote-ref-18)
20. R. Weigel et al. Effect of provider-initiated testing and counselling and integration of ART services on access to HIV diagnosis and treatment for children in Lilongwe, Malawi: a pre- post comparison. *BMC Pediatrics*, 9:80, 2009. [↑](#endnote-ref-19)
21. S. Topp et al. Opt-out provider-initiated HIV testing and counselling in primary care outpatient Health facilities in Zambia. *Bulletin: World Health Organization*, 89:328-335, 2011. [↑](#endnote-ref-20)
22. Violari et al. Early antiretroviral therapy and mortality among HIV-infected infants. New England Journal of Medicine. Nov 20;359(21):2233-44. 2008. [↑](#endnote-ref-21)
23. Newell, ML et al. (2004) [↑](#endnote-ref-22)
24. Denominator is the number of mothers with unknown or prior negative HIV status presenting at the health facility for their infant’s routine six week immunizations [↑](#footnote-ref-2)
25. Denominator is the number of women presenting at the health facility for their infant’s routine six week immunizations [↑](#footnote-ref-3)
26. Denominator is the number of known HIV-exposed infants presenting at the health facility for routine six week immunizations [↑](#footnote-ref-4)
27. For each health facility, the denominator for the percentage calculation will be # of pregnant women attending ANC. This figure can subsequently be converted to an estimate of # of women giving birth in a given health facilities catchment area. Importantly, the primary indicator is the *percentage change* of the indicator – thus, this metric will be usable even in the rare / unlikely case that the # infants immunized exceed the # infants presenting at least once for ANC. [↑](#footnote-ref-5)
28. First immunizations refer to the 6-week visit for under-five health facility where children receive the following vaccinations: OPV1 and DPT1-HepB-HiB. The Second immunization refers to the 10-week visit to under-five health facility for OPV2 and DPT2 vaccines. [↑](#footnote-ref-6)
29. Analysis will include the use of historical immunization data as covariates which will increase power to a level greater than 80%. For the sample frame, at least 60 to 70% of the variation in immunization rates on a month to month basis can be explained by the previous year of data. [↑](#footnote-ref-7)
30. This assumes that the simple intervention will have the effect assumed in the previous power calculation because this calculation compares comprehensive clinics to simple clinics. [↑](#footnote-ref-8)
31. This assumes that of the approximately 45,000 babies tested currently for a DNA PCR, around two-thirds are first test PCR (done at around the 6-week time frame) and the remaining 1/3 are retests. This means that 30,000 out 600,000 babies born each year receive a PCR test at 6 weeks - around 5%. [↑](#footnote-ref-9)
32. As in the case of the immunization data, covariates will be used to increase power for this outcome [↑](#footnote-ref-10)
33. This assumes that the simple intervention will have the effect assumed in the previous power calculation because this calculation compares comprehensive clinics to simple clinics. [↑](#footnote-ref-11)
34. A linear regression measuring the effect of the Comprehensive Intervention on immunization uptake will be used to assess any decrease in immunizations. “Confirmed” indicates a drop of 20 percentage points is observed at a 95% confidence level. [↑](#footnote-ref-12)
35. National Protocol Guidelines, Integrated Prevention of Mother-to-Child Transmission of HIV/AID in Zambia, 2008 Page 8. [↑](#endnote-ref-23)
